# Zinc(II) Complexes with Triplet Charge-Transfer Excited States Enabling Energy-Transfer Catalysis, Photoinduced Electron Transfer, and Upconversion

**DOI:** 10.1021/jacsau.2c00442

**Published:** 2022-10-11

**Authors:** Jasmin A. Kübler, Björn Pfund, Oliver S. Wenger

**Affiliations:** Department of Chemistry, University of Basel, St. Johanns-Ring 19, 4056 Basel, Switzerland

**Keywords:** first-row transition-metal complexes, earth-abundant metals, coordination chemistry, photocatalysis, spectroscopy

## Abstract

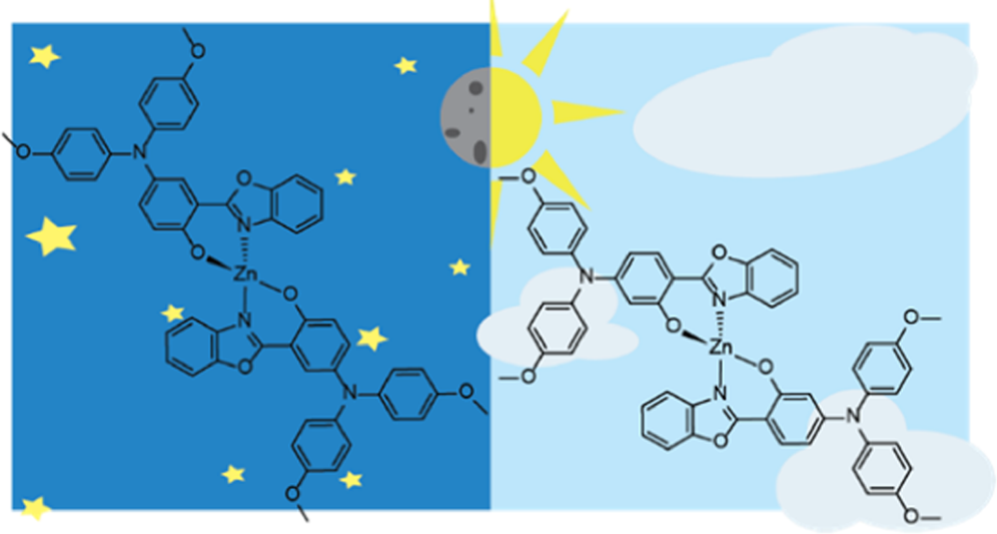

Many Cu^I^ complexes have luminescent triplet charge-transfer excited states with diverse applications in photophysics and photochemistry, but for isoelectronic Zn^II^ compounds, this behavior is much less common, and they typically only show ligand-based fluorescence from singlet π–π* states. We report two closely related tetrahedral Zn^II^ compounds, in which intersystem crossing occurs with appreciable quantum yields and leads to the population of triplet excited states with intraligand charge-transfer (ILCT) character. In addition to showing fluorescence from their initially excited ^1^ILCT states, these new compounds therefore undergo triplet–triplet energy transfer (TTET) from their ^3^ILCT states and consequently can act as sensitizers for photo-isomerization reactions and triplet–triplet annihilation upconversion from the blue to the ultraviolet spectral range. The photoactive ^3^ILCT state furthermore facilitates photoinduced electron transfer. Collectively, our findings demonstrate that mononuclear Zn^II^ compounds with photophysical and photochemical properties reminiscent of well-known Cu^I^ complexes are accessible with suitable ligands and that they are potentially amenable to many different applications. Our insights seem relevant in the greater context of obtaining photoactive compounds based on abundant transition metals, complementing well-known precious-metal-based luminophores and photosensitizers.

## Introduction

Tetrahedral copper(I) complexes with luminescent and redox-active metal-to-ligand charge-transfer (MLCT) excited states have long been known^1,2^ and nowadays represent a well-developed compound class with applications in light-emitting devices,^3−7^ dye-sensitized solar cells,^8^ and photocatalysis.^9−16^ In the 3d^10^ valence electron configuration of Cu^I^, there are no metal-centered (MC) excited states that can depopulate the photoactive ^3^MLCT states,^17,18^ unlike for analogous 3d^6^ (Co^III^, Fe^II^, Mn^I^, Cr^0^)^19−25^ or 3d^8^ (Ni^II^) compounds,^26,27^ in which nonradiative MLCT deactivation by MC states can be undesirably fast.^28^ Consequently, complexes with the 3d^10^ configuration are predisposed for obtaining long-lived and strongly emissive excited states, as illustrated for example by recently reported linear two-coordinate Cu^I^ compounds with exceptionally strongly luminescent ligand-to-ligand charge-transfer (LLCT) excited states.^29−33^

Isoelectronic Zn^II^ compounds have received less attention, presumably for several reasons. The higher oxidation state of Zn^II^ relative to Cu^I^ shifts MLCT excited states to higher energies and usually leads to situations, in which ligand-based (or other) states are energetically lower and therefore dominate the photophysical and photochemical properties.^34^ Moreover, Zn^II^ complexes have a tendency to form polynuclear compounds,^35,36^ many of which are labile and for which proper characterization and speciation in solution are not trivial because multiple species can exist in dynamic equilibrium with each other.^37,38^ Thus, the lack of ligand field stabilization energy in the 3d^10^ valence electron configuration can make it more challenging to obtain easily characterizable and substitution-inert mononuclear complexes compared to the above-mentioned 3d^6^ or 3d^8^ configurations.

The most frequently observed emission type in Zn^II^ complexes until now is ligand-based fluorescence, for example, in many dipyrrin-based compounds,^39−45^ complexes with benzothiazole ligands,^46−48^ as well as with other nitrogen and oxygen donor ligands,^49,50^ or phosphines.^51^ Possible applications of fluorescent Zn^II^ compounds in electroluminescence,^46,52−54^ including organic light-emitting diodes (OLEDs),^55−57^ circularly polarized luminescence,^58^ sensing,^59^ or cell imaging, have attracted interest.^60^ Similarly, aggregation-induced emission^61−65^ as well as thermally activated delayed fluorescence (TADF) received substantial attention with Zn^II^ complexes.^66−69^

One of the most recent fundamental developments concerning photoactive Zn^II^ compounds pursues the idea to introduce other emission types than ligand-based fluorescence, for example, states with triplet and/or charge-transfer character in halide complexes.^70−72^ To date, this specific research thrust seems to concentrate largely on the solid state, presumably because phosphorescence and charge-transfer emission are trickier to obtain in fluid solution at room temperature. The solution phase is however very relevant for photoactive triplet and charge-transfer excited states, due to possible applications in triplet energy-transfer catalysis,^73^ triplet–triplet annihilation upconversion,^74^ photoinduced electron transfer, and photoredox catalysis,^75^ including perspectives for solar energy conversion.^76^ In the course of our research program on photoactive complexes based on abundant transition metals,^28,77^ we therefore aimed to explore the possibility of obtaining Zn^II^ compounds with triplet charge-transfer excited states and furthermore wished to assess their potential for usage in energy-transfer catalysis, upconversion, and photoinduced electron transfer.

**Figure 1:**
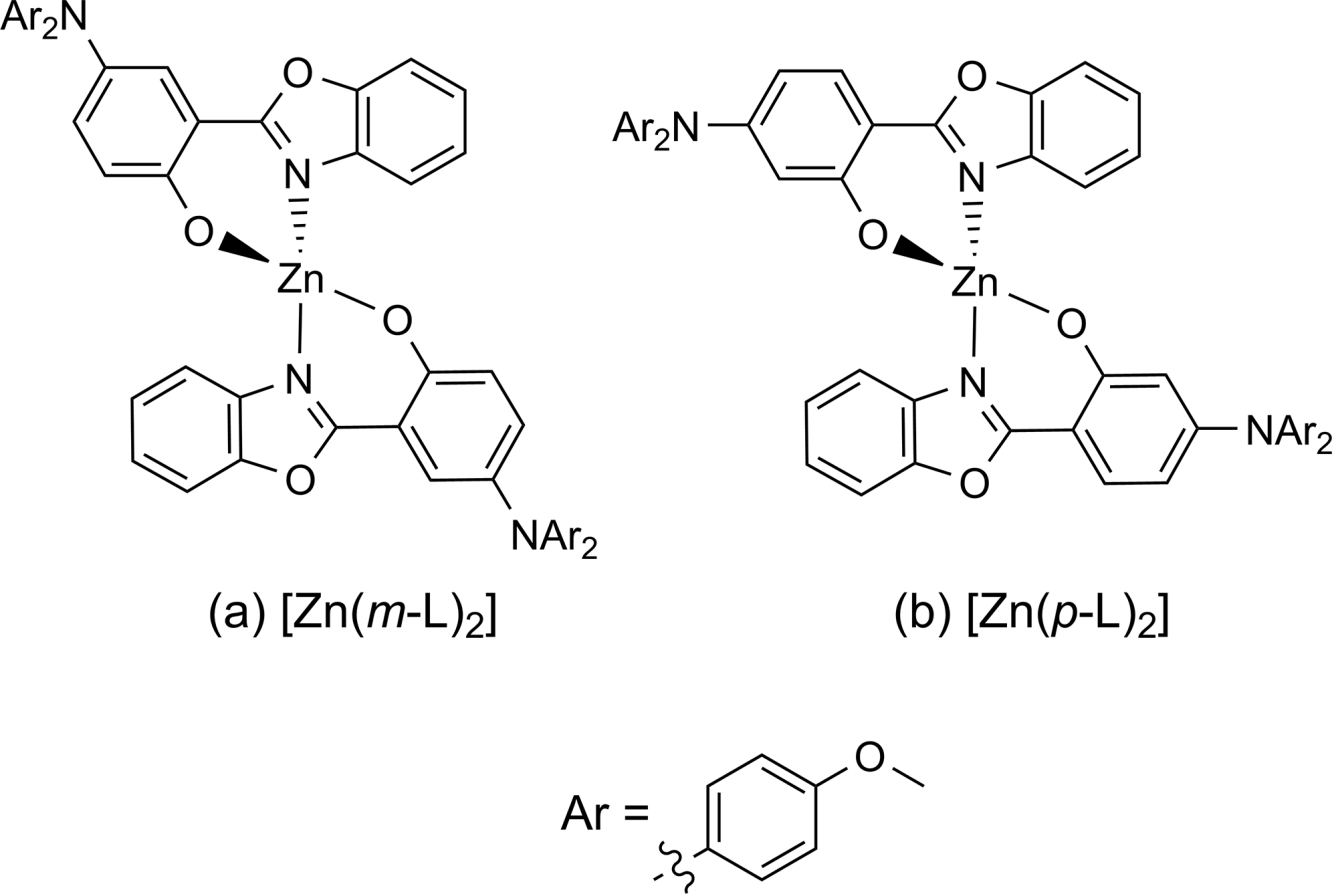
Molecular structures of the two complexes synthesized and investigated herein. Only one of two possible stereoisomers is displayed for each complex (the individual enantiomers are shown schematically in Figure S1).

Our molecular design was inspired by an earlier study of Zn^II^-based TADF emitters with (2-hydroxyphenyl)benzoxazole ligands,^66^ to which we attached di(anisyl)amino substituents in an attempt to enhance the charge-transfer character between the electron-rich phenolate ligand moiety and the electron-deficient benzoxazole subunit (Figure 1). We anticipated that this design introduces photoactive intraligand charge-transfer (ILCT) excited states and that the metal center will contribute to mediating intersystem crossing. The anticipated ^3^ILCT excited state was further expected to be less distorted than the MLCT excited state of four-coordinate Cu^I^ complexes, in which Jahn–Teller distortions leading to nonradiative deactivation can typically occur.^3,17,78,79^

## Results and Discussion

### Synthesis and Characterization

Two new ligands with di(anisyl)amino substituents either in *meta*- (*m*-LH) or in *para*-position (*p*-LH) to the benzoxazole unit were synthesized and fully characterized, as described in detail in the Supporting Information (SI, pages S3–S14). The electronic coupling between the tertiary amine donor and the benzoxazole acceptor subunits is expected to be different in *m*-LH and *p*-LH, which in turn should lead to distinct properties of the anticipated ILCT excited states in [Zn(*m*-L)_2_] and [Zn(*p*-L)_2_]. Complexation was achieved by refluxing two equivalents of *m*-LH or *p*-LH with Zn(OAc)_2_ in tetrahydrofuran (THF) or toluene overnight, following a previously published protocol for somewhat related Zn^II^ compounds.^66^ In our case, this led to products with ill-defined ^1^H-NMR spectra, and MALDI-TOF-MS furthermore indicated the formation of dinuclear [Zn_2_(*m*-L)_3_] or [Zn_2_(*p*-L)_3_] complexes with *m*/*z* = 1443, in addition to the anticipated signals for [Zn(*m*-L)_2_] or [Zn(*p*-L)_2_] at *m*/*z* = 938. Evidently, product mixtures were present, and therefore the yellow powders were heated to 220 °C in a sublimation apparatus under vacuum overnight, which resulted in the deposition of free *m*-LH or *p*-LH ligand on the cooling finger. The remaining powders in the sublimation flask gave well-defined NMR signals for pure [Zn(*m*-L)_2_] and [Zn(*p*-L)_2_], and the MALDI-TOF-MS signals for the dinuclear [Zn_2_(*m*-L)_3_] or [Zn_2_(*p*-L)_3_] complexes were no longer present. The identity and purity of the mononuclear target complexes were furthermore confirmed by elemental analysis and HR-ESI-MS.

### Photophysical Properties

**Figure 2:**
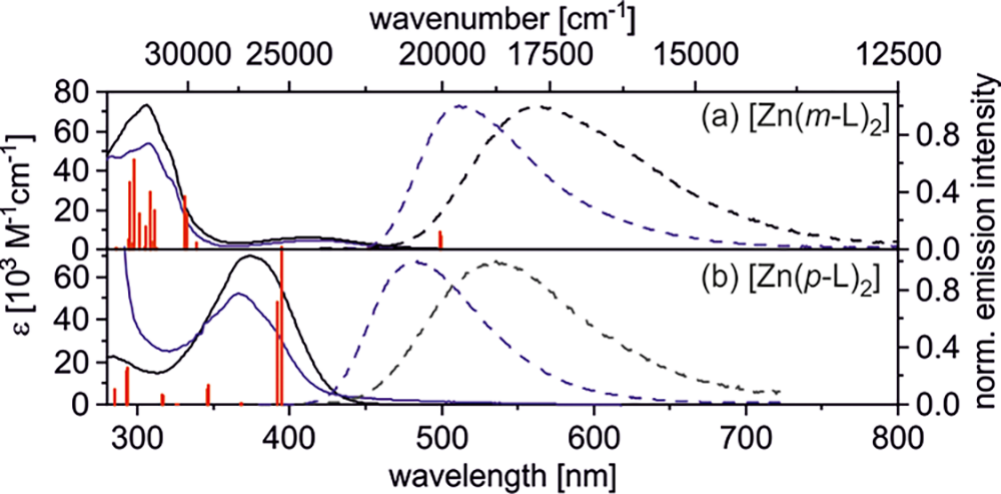
UV–vis absorption (solid lines) and normalized emission spectra (dashed lines) of (a) [Zn(*m*-L)_2_] (excited at 419 nm) and (b) [Zn(*p*-L)_2_] (excited at 370 nm) in deaerated toluene (blue) and deaerated CH_2_Cl_2_ (black) at 293 K. The relative oscillator strengths of the first 60 vertical transitions (S_1_–S_60_) were calculated by TD-DFT. Vertical transitions with energy below 4.43 eV (corresponding to 280 nm) are displayed as red vertical lines.

The UV–vis absorption spectra of [Zn(*m*-L)_2_] and [Zn(*p*-L)_2_] in toluene and dichloromethane (solid blue and black traces in Figure 2) are very similar to the spectra of the respective free ligands measured under identical conditions (Figures S2 and S3). Wavelength differences of absorption band maxima between complexes and respective free ligands are minimal, but the molar extinction coefficients are roughly twice as high for the complexes, due to the presence of two ligands per complex.

Strong emission solvatochromism is observed for both the complexes and the free ligands, manifesting in a redshift of about 2000 cm^–1^ when going from toluene to CH_2_Cl_2_. This observation suggests that the electronic transition responsible for the emission has substantial charge-transfer character, as expected. When comparing the absorption and emission properties of the two complexes, the key differences are that [Zn(*m*-L)_2_] has a distinct and comparatively weak absorption band in the visible region (with ε = 4550 M^–1^ cm^–1^ at the maximum at 417 nm in toluene), while the lowest absorption band maximum of [Zn(*p*-L)_2_] is in the UV (366 nm in toluene) and features a substantially higher molar extinction coefficient at this wavelength (ε = 52,000 M^–1^ cm^–1^). The emission band maximum of [Zn(*m*-L)_2_] is red-shifted compared to [Zn(*p*-L)_2_] (28 nm in toluene and 27 nm in CH_2_Cl_2_). DFT calculations (SI, pages S55–S66) reproduce the experimentally observable main differences in UV–vis absorption spectroscopy. Specifically, distinct transitions at wavelengths longer than 400 nm for [Zn(*m*-L)_2_] are obtained (red vertical lines in Figure 2a), whereas for [Zn(*p*-L)_2_], no such long-wavelength absorption transitions are calculated (red vertical lines in Figure 2b).

**Figure 3:**
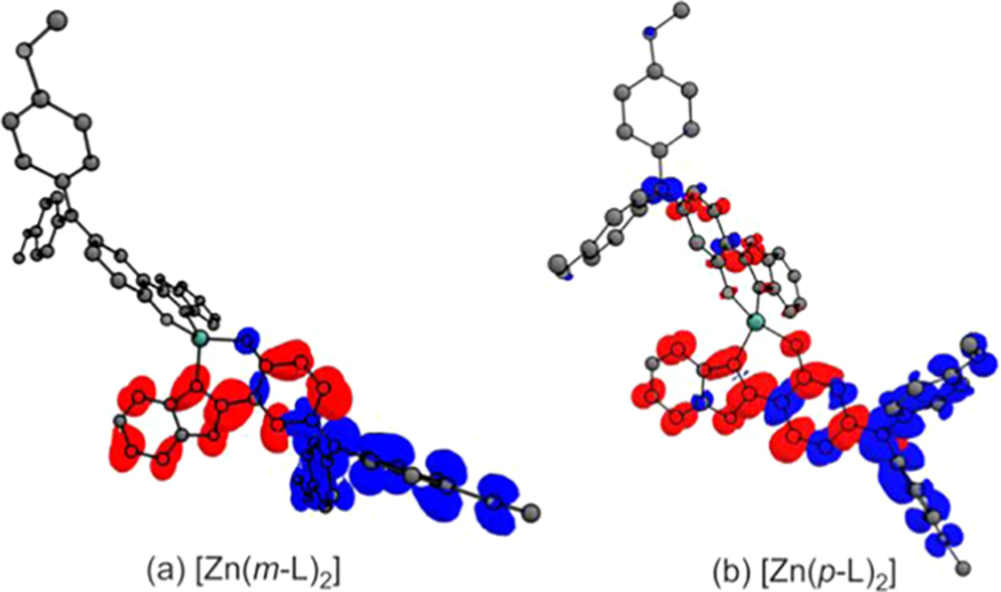
Highest occupied natural transition orbital (HONTO, blue) and lowest unoccupied natural transition orbital (LUNTO, red) of the first vertical transition (S_0_ → S_1_) of (a) [Zn(*m*-L)_2_] and (b) [Zn(*p*-L)_2_]. The Zn^II^ cation is colored in green.

**Figure 4:**
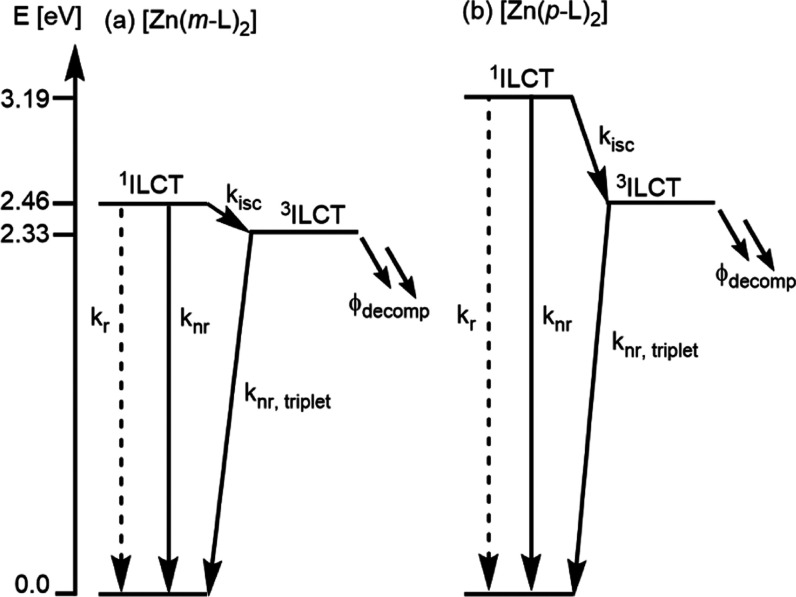
Energy-level diagrams with relative energies of the energetically lowest ^1^ILCT and ^3^ILCT excited states of [Zn(*m*-L)_2_] and [Zn(*p*-L)_2_], as calculated by TD-DFT (PBE1PBE/def2SV(P), PCM(CH_2_Cl_2_); for details, see SI page S55). The lowest ^1^ILCT state of [Zn(*m*-L)_2_] and the lowest ^3^ILCT state of [Zn(*p*-L)_2_] coincidentally have the same energy (2.46 eV). Rate constants (*k*) for radiative (r) and nonradiative (nr) relaxation processes as well as intersystem crossing (isc) were determined experimentally and are summarized in Table 1. Φ_decomp_ represents the quantum yield for a chemical decomposition process.

The DFT calculations furthermore confirm the anticipated intraligand charge-transfer (ILCT) character of the lowest-energy transitions in the two Zn^II^ complexes (Figure 3). Specifically, the S_0_ → S_1_ transition of both complexes involves a depletion of electron density from the tertiary amine units (marked blue in Figure 3) and a corresponding increase in the phenoxazole moieties (colored in red), with very little metal involvement in both cases. In [Zn(*m*-L)_2_], the HOMO is almost exclusively located on the bis(4-methoxyphenyl)amine units (Figures 3a and S44), whereas in [Zn(*p*-L)_2_], the HOMO is partly distributed over the 2-phenylbenzoxazole unit (Figure 3b, S45, and S46). The LUMOs of both complexes are distributed over the remaining ligand backbone without the bis(4-methoxyphenyl)amine. Based on the experimental UV–vis absorption and emission spectra, the ^1^ILCT energies are 2.65 eV (467 nm) for [Zn(*m*-L)_2_] and 2.89 eV (429 nm) for [Zn(*p*-L)_2_], whereas the calculations yield 2.46 eV for [Zn(*m*-L)_2_] and 3.19 eV for [Zn(*p*-L)_2_]. The experimental values for the ^1^ILCT energies were estimated by normalizing the lowest-energy absorption bands and the emission bands of the [Zn(*m*-L)_2_] and [Zn(*p*-L)_2_] complexes, and by determining the intersection of the corresponding absorption and emission bands (Figure S4). Attempts to determine the respective ^3^ILCT energies by recording 77 K emission spectra for [Zn(*m*-L)_2_] and [Zn(*p*-L)_2_] were unsuccessful because no phosphorescence was detectable. Instead, the 77 K emission spectra resembled those recorded at room temperature, indicating that the ^1^ILCT remains the only emissive excited state even at 77 K. Consequently, the ^3^ILCT energies were estimated from the TD-DFT calculations, and the energy-level scheme in Figure 4 is therefore entirely based on calculated state energies for internal consistency reasons.

The calculated energy difference between the lowest ^1^ILCT and ^3^ILCT excited states (Δ*E*_ST_) is substantially smaller for [Zn(*m*-L)_2_] (0.13 eV) than for [Zn(*p*-L)_2_] (0.73 eV). This can be qualitatively rationalized on the basis of the clearer spatial separation between HOMO and LUMO in [Zn(*m*-L)_2_] in comparison to [Zn(*p*-L)_2_] (due to decreased electron–electron interaction), as discussed above (Figure 3).

**Figure 5:**
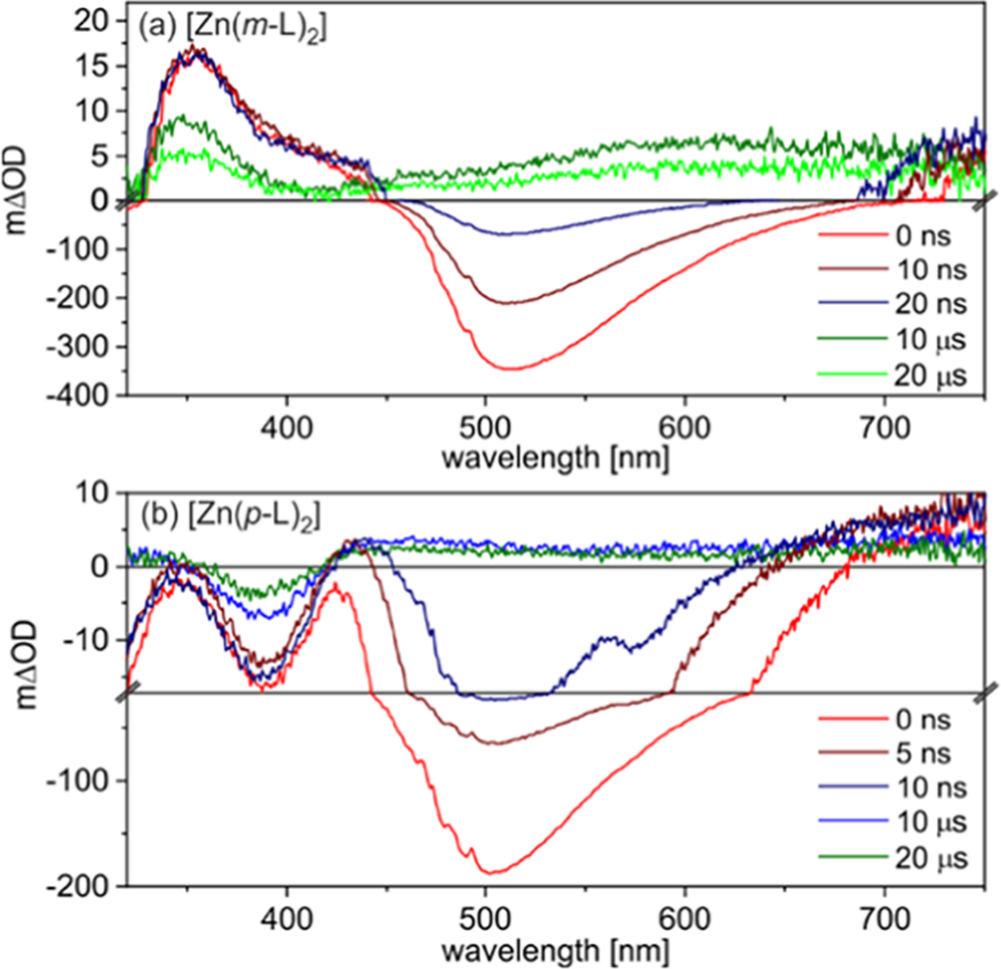
Transient absorption spectra of (a) [Zn(*m*-L)_2_] and (b) [Zn(*p*-L)_2_] in deaerated toluene at 293 K, recorded at different time delays as indicated by the legends. [Zn(*m*-L)_2_] was excited at 424 nm with laser pulse energies of 11 mJ, whereas [Zn(*p*-L)_2_] was excited at 355 nm using laser pulses with 35 mJ energy. Note the scale changes at mΔOD = 0 in (a) and at mΔOD = −17 in (b). All spectra were recorded with an integration time of 200 ns after the indicated delay times.

The UV–vis transient absorption spectra recorded at short delay times (<20 ns) after the excitation pulses are dominated by negative signals centered around 500 nm (Figure 5), which coincide with the fluorescence bands in Figure 2. On longer time scales (microseconds), the transient absorption spectrum of [Zn(*m*-L)_2_] in deaerated toluene (Figure 5a) is dominated by an excited state absorption (ESA) band at 350 nm and a somewhat weaker ESA band centered around 610 nm. The transient difference absorption spectrum of [Zn(*p*-L)_2_] at long delay times (>20 ns) shows a very broad ESA band with a maximum at 450 nm along with a ground-state bleach at 390 nm. Evidently, [Zn(*m*-L)_2_] and [Zn(*p*-L)_2_] both have a fluorescent singlet excited state (^1^ILCT) and a much longer-lived (nonemissive) triplet excited state (^3^ILCT), which causes the above-mentioned ESA bands. In aerated solutions of [Zn(*m*-L)_2_] and [Zn(*p*-L)_2_], the ^3^ILCT excited states are efficiently quenched by oxygen (Figures 5 and S6), in line with their triplet character. Based on time-resolved fluorescence experiments (Figures S7b and S8b), the ^1^ILCT lifetimes (τ_singlet_) of [Zn(*m*-L)_2_] and [Zn(*p*-L)_2_] are 25 and 5.5 ns, respectively, in deaerated toluene at 293 K. The ^1^ILCT fluorescence decay of [Zn(*m*-L)_2_] is biexponential in both toluene and CH_2_Cl_2_; see SI page S23 for details. We speculate that the biexponential nature of the excited-state decay for this compound stems from different conformers that are present in solution. The τ_singlet_ values reported for [Zn(*m*-L)_2_] in Table 1 therefore represents a weighted average (SI page S23).

**Table 1:** Experimental Values of ^1^ILCT (τ_singlet_) and ^3^ILCT Lifetimes (τ_triplet_), Fluorescence and Intersystem Crossing Quantum Yields (Φ_fluo_ and Φ_isc_) in Deaerated Toluene at 293 K^a^^b^

	τ_singlet_ [ns]	τ_triplet_ [μs]	Φ_fluo_ [%]	Φ_isc_ [%]	*k*_tot_ [s^–1^]	*k*_r_ [s^–1^]	*k*_nr_ [s^–1^]	*k*_isc_ [s^–1^]	*k*_nr,triplet_ [s^–1^]
*m*-LH	24		22		4.2 × 10^7^	9.2 × 10^6^	3.3 × 10^7^		
[Zn(*m*-L)_2_]	25^b^	38	50	13	4.0 × 10^7^	2.0 × 10^7^	1.5 × 10^7^	5.2 × 10^6^	2.6 × 10^4^
*p*-LH	5.5		94		1.8 × 10^8^	1.7 × 10^8^	1.0 × 10^7^		
[Zn(*p*-L)_2_]	5.5	62	86	7.2	1.8 × 10^8^	1.5 × 10^8^	1.2 × 10^7^	1.3 × 10^7^	1.6 × 10^4^

a The radiative (*k*_r_) and nonradiative rate constants (*k*_nr_) for ^1^ILCT decay, as well as the rate constant for nonradiative ^3^ILCT decay (*k*_nr,triplet_) as illustrated in Figure 4 are as indicated.

b Lifetimes of biexponential decays were averaged in a weighted manner (see SI page S22 for details).

Expectedly, the fluorescence lifetimes of the free ligands and corresponding complexes are very similar to each other. In toluene, the ^1^ILCT lifetime of [Zn(*m*-L)_2_] is longer (25 ns) than in CH_2_Cl_2_ (9 ns), in line with the energy gap law, though for [Zn(*p*-L)_2_] such a difference is not observed and the ^1^ILCT lifetime is 5.5 ns in both solvents (Table S3). The fluorescence quantum yield (Φ_fluo_) of [Zn(*p*-L)_2_] (86% in deaerated toluene at 293 K, Table 1) is substantially higher than that of [Zn(*m*-L)_2_] under identical conditions (50%). With ^1^ILCT lifetimes and luminescence quantum yields at hand, the rate constants for radiative (*k*_r_) and nonradiative excited-state relaxation (*k*_nr_) were calculated following eqs S5–S9. The intense fluorescence signals and rapid intersystem crossing complicate the detection of the spectral signatures of the ^1^ILCT states by transient absorption, but it seems plausible that the ^1^ILCT and ^3^ILCT states have in fact similar spectroscopic signatures (Figures 5 and S6).^80^ Based on their very different decay kinetics, the distinction between these two states is however clear-cut.

**Figure 6:**
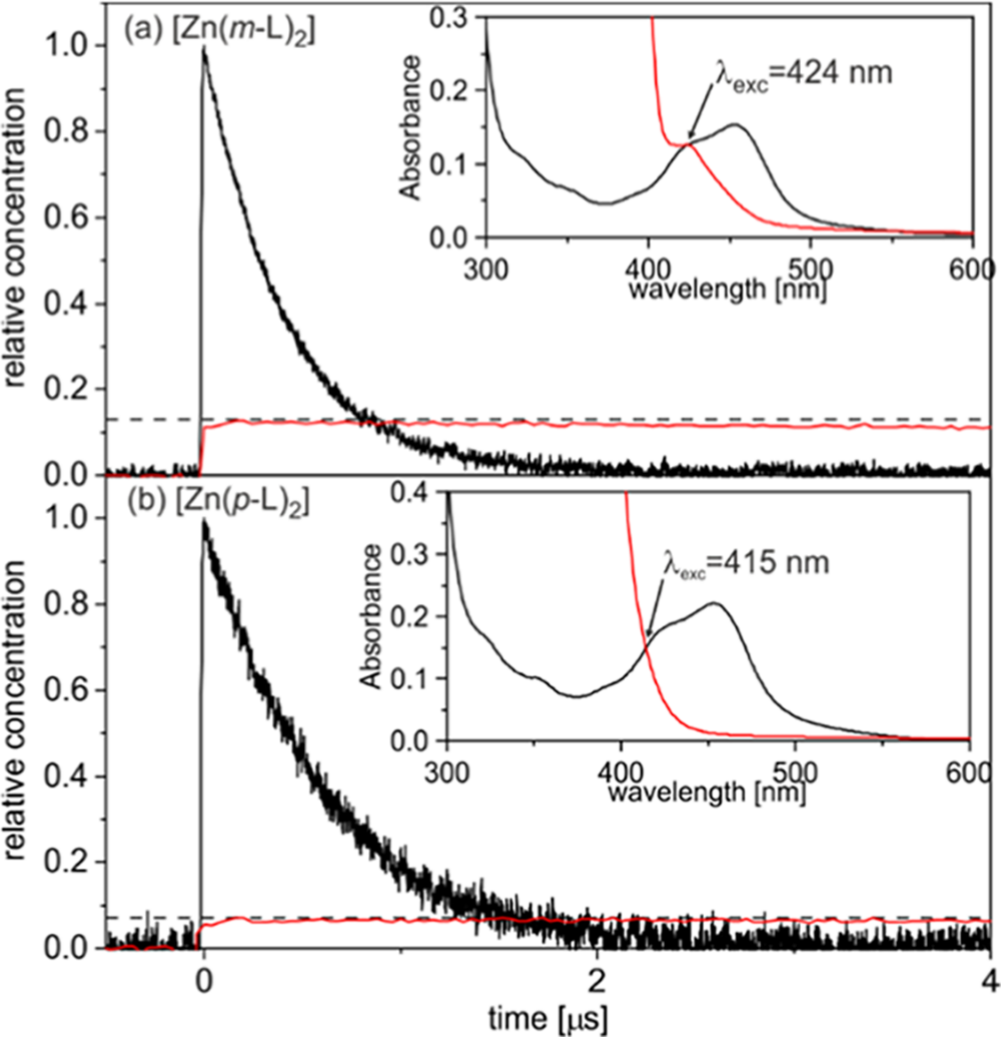
Determination of the intersystem crossing quantum yield (Φ_isc_) by monitoring the formation of triplet-excited anthracene following excitation of [Zn(*m*-L)_2_] and [Zn(*p*-L)_2_] in the presence of 10 mM anthracene. Solutions of [Ru(bpy)_3_]^2+^ in deaerated H_2_O at 293 K with identical absorbance at the excitation wavelengths (λ_exc_ = 424/415 nm, insets) were used to determine the number of photons absorbed by the individual solutions. The black trace in both main plots is the ^3^MLCT excited state decay of [Ru(bpy)_3_]^2+^ monitored at 455 nm, where Δε = −10,100 M^–1^ cm^–1^ upon ^3^MLCT excitation.^82^ The red traces in both main plots represent the relative amounts of triplet-excited anthracene following excitation of (a) 27 μM [Zn(*m*-L)_2_] at 424 nm and (b) 8.5 μM [Zn(*p*-L)_2_] at 415 nm in deaerated toluene at 293 K, both in the presence of 10 mM anthracene (determined by monitoring the diagnostic triplet anthracene absorption band at 423 nm with ε = 53,000 M^–1^ cm^–1^).^81^ Assuming that triplet–triplet energy transfer from the ^3^ILCT excited state of the Zn^II^ complexes is quantitative under these conditions, the intersystem crossing quantum yields can be estimated from the transient absorption signals at *t* = 0 (red vs black decay traces). The insets show UV–vis (ground state) absorption spectra of all used solutions, demonstrating that [Ru(bpy)_3_]^2+^ (black) and the solutions of the Zn^II^ complexes absorbed equal amounts of light at the used excitation wavelengths (λ_exc_).

To obtain quantitative information concerning the intersystem crossing (ISC) from the initially excited ^1^ILCT to the (dark) ^3^ILCT state, relative actinometry experiments were performed. For this purpose, the molar quantities of triplet-excited anthracene formed after selective excitation of [Zn(*m*-L)_2_] and [Zn(*p*-L)_2_] in the presence of excess anthracene were determined by transient UV–vis absorption spectroscopy (Figure 6). Assuming that triplet–triplet energy transfer from the Zn^II^ complexes to anthracene is quantitative (which seems reasonable given its high driving force) and given the known molar extinction coefficient of triplet-excited anthracene at 423 nm (53,000 M^–1^ cm^–1^),^81^ the concentration of triplet-excited [Zn(*m*-L)_2_] and [Zn(*p*-L)_2_] can be estimated in this manner. In a separate experiment, an aqueous solution of [Ru(bpy)_3_]^2+^ with the same absorbance at the respective excitation wavelength was irradiated using identical instrument settings. By monitoring the bleach of the corresponding MLCT absorption band at 455 nm with its known change in molar extinction coefficient (Δε = −10,100 M^–1^ cm^–1^),^82^ the number of photons absorbed by the individual solutions was determined; see SI page S20 for full details. The intersystem crossing quantum yield (Φ_isc_) was then approximated as the number of triplet-excited Zn^II^ complexes divided by the number of absorbed photons.

For [Zn(*m*-L)_2_], we obtain Φ_isc_ = 13%, whereas for [Zn(*p*-L)_2_], Φ_isc_ is substantially lower with merely 7.2%. This finding is compatible with the lower fluorescence quantum yield found for [Zn(*m*-L)_2_] (50%) relative to [Zn(*p*-L)_2_] (86%), and furthermore is in qualitative accordance with the smaller ^1^ILCT–^3^ILCT energy gap (Δ*E*_ST_) in [Zn(*m*-L)_2_] (0.13 vs 0.73 eV, Figure 4), as *k*_isc_ is often inversely proportional to Δ*E*_ST_.^83^

By monitoring the decay of the excited-state absorption band at 350 nm in Figure 5a (Figure S9) and the bleach recovery at 390 nm in Figure 5b (Figure S10), the lifetimes of the photoactive (but dark) ^3^ILCT excited states (τ_triplet_) of [Zn(*m*-L)_2_] and of [Zn(*p*-L)_2_] were determined. We find τ_triplet_ = 38 μs for [Zn(*m*-L)_2_] and τ_triplet_ = 62 μs for [Zn(*p*-L)_2_] in deaerated toluene at 293 K.

### Triplet Energy-Transfer Catalysis

Since both [Zn(*m*-L)_2_] and [Zn(*p*-L)_2_] have appreciable intersystem crossing quantum efficiencies (Table 1 and Figure 6), we decided to explore to what extent these compounds can sensitize organic triplet excited-state reactions, such as photo-isomerizations and a photo-fragmentation. We used a 415 nm LED as an excitation source for these purposes. For the first experiment, we adjusted the [Zn(*m*-L)_2_] and [Zn(*p*-L)_2_] concentrations such that an optical density of 0.06 resulted at that wavelength, which implied substantially different complex concentrations (1.3 vs 0.6 mol % relative to the substrates) due to their different molar extinction coefficients at 415 nm (Figure 2). This procedure was chosen to ensure that the different reaction mixtures absorb equal amounts of excitation light in a given irradiation period.

Under these conditions, the photo-isomerization of *trans*-stilbene reached the photostationary state after about 60 min (Figure S22). With [Zn(*m*-L)_2_] as photosensitizer, that photostationary state was composed of 81% *cis*-stilbene, whereas the [Zn(*p*-L)_2_] complex yielded a photostationary state with only 49% of *cis*-stilbene (Table 2, entry 1). This experimental observation can be understood on the basis of the different triplet energies of the two photosensitizers (Figure 4) and the different triplet energies of *trans*-stilbene (2.14 eV) and *cis*-stilbene (2.35 eV) (Figure S18).^84^ The ^3^ILCT energy of [Zn(*m*-L)_2_] (2.33 eV) lies between the triplet energies of *trans*- and *cis*-stilbene, and consequently this complex sensitizes the *trans*-to-*cis* photo-isomerization of stilbene far more efficiently than the reverse reaction, resulting in the accumulation of the *cis*-isomer over time (Table 2, entry 1). By contrast, the photoactive ^3^ILCT state of [Zn(*p*-L)_2_] (2.46 eV) is energetically above the lowest triplet states of both *trans*- and *cis*-stilbene. Consequently, [Zn(*p*-L)_2_] sensitizes the *cis*-to-*trans* and the *trans*-to-*cis* photo-isomerization of this specific substrate equally well, resulting in a photostationary state composed of roughly 1:1 *cis*- and *trans*-products (Table 2, entry 1, last column). Though photo-isomerizations of stilbene compounds have been reported after direct excitation of their singlet excited states,^85−87^ we have found no evidence that the fluorescent ^1^ILCT excited states of our zinc(II) complexes sensitize this reaction.

**Table 2:**
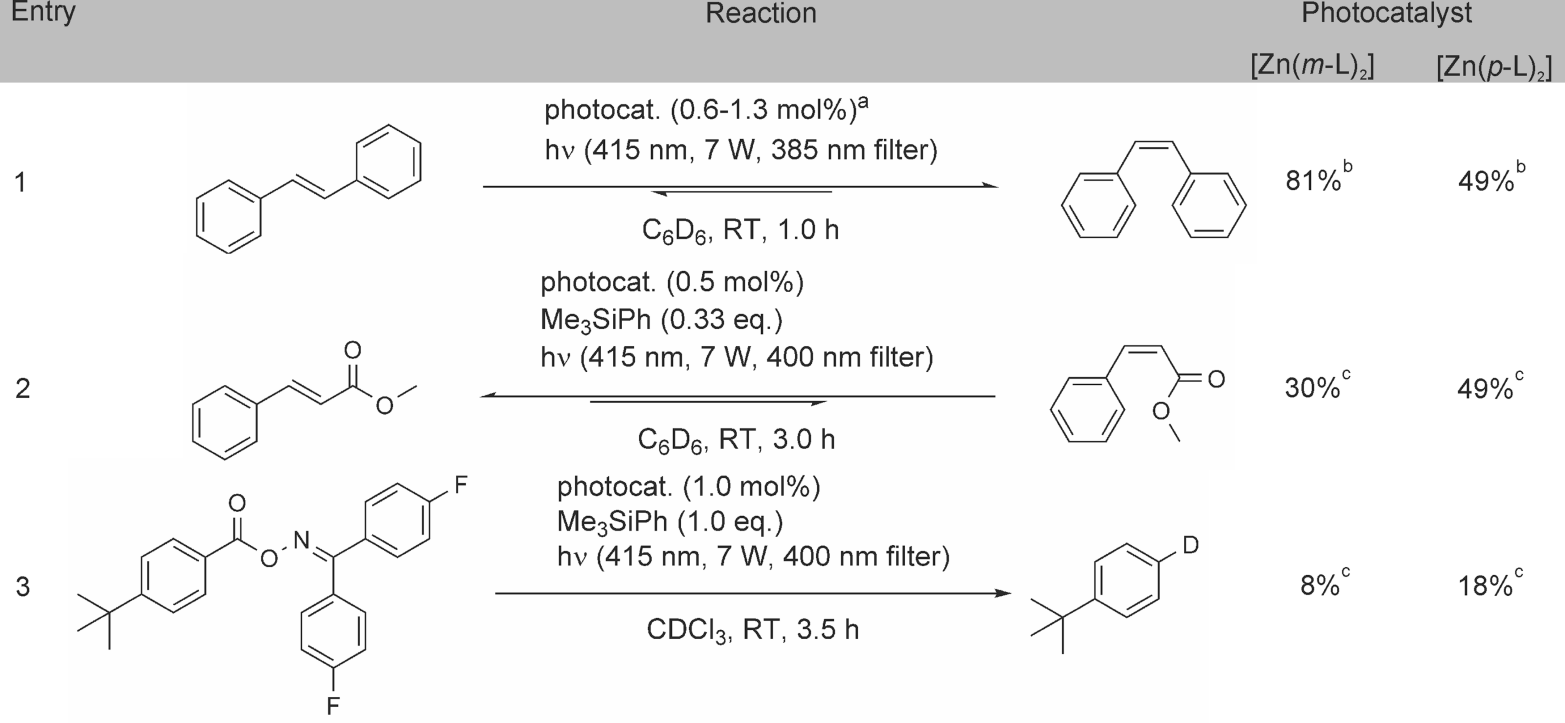
Sensitized Triplet–Triplet Energy-Transfer (TTET) Reactions

a Optical densities adjusted to the same value of about 0.06 at the excitation wavelength of 415 nm for both complexes, resulting in 1.3 mol % [Zn(*m*-L)_2_] and 0.6 mol % [Zn(*p*-L)_2_].

b Yields were determined by comparing ^1^H-NMR integrals of the reactant and the product.

c Yields were calculated relative to the internal standard Me_3_SiPh using ^1^H-NMR spectroscopy.

For the methyl cinnamate substrate (Table 2, entry 2), the [Zn(*m*-L)_2_] photosensitizer leads to a photostationary state containing only 30% of the *cis*-isomer, which is considerably lower than what is obtained in the case of stilbene (81%). This observation can be understood on the basis of the higher triplet energy of *trans*-methyl cinnamate (2.37 eV)^84^ compared to *trans*-stilbene (2.14 eV).^84^ Consequently, [Zn(*m*-L)_2_] with its ^3^ILCT energy of 2.33 eV becomes a less efficient sensitizer for the *cis*-to-*trans* isomerization of methyl cinnamate. When using [Zn(*p*-L)_2_] as a sensitizer for the methyl cinnamate substrate, a substantially higher *cis* photoproduct yield of 49% is reached, due to the higher ^3^ILCT energy of this complex in comparison to [Zn(*m*-L)_2_]. For the methyl cinnamate photo-isomerization reactions, we used an identical photosensitizer loading of 0.5 mol % in both experiments. When reducing the catalyst load to 0.05 mol %, we determined a turnover number (TON) of 550 for [Zn(*m*-L)_2_] and 940 for [Zn(*p*-L)_2_].

To explore organic triplet reactivity beyond photo-isomerizations, we focused on a decarboxylation reaction (Table 2, entry 3), which was previously sensitized by an iridium complex with a triplet energy of 2.64 eV.^88^ Following excitation to its lowest-lying triplet state, this substrate is known to undergo a photo-fragmentation reaction that is triggered by the release of CO_2_, forming an aryl radical intermediate. The latter can be intercepted by CDCl_3_ under uptake of a deuterium atom, which makes the respective fragmentation product (Table 2, entry 3) easily detectable by NMR spectroscopy. Unfortunately, in CDCl_3_ the solubility of [Zn(*m*-L)_2_] and [Zn(*p*-L)_2_] is limited and furthermore both complexes suffer from lower stability in CDCl_3_ compared to less polar solvents such as benzene or toluene. Conversely, the substrate is not sufficiently well soluble in benzene-*d*_6_ and toluene-*d*_8_. Consequently, only relatively modest decarboxylation yields of 8% when using [Zn(*m*-L)_2_] and 18% in the case of [Zn(*p*-L)_2_] were obtained (Table 2, entry 3; Figures S27–S30). Upon irradiation of a solution of the substrate without Zn^II^ photosensitizer, no decarboxylation product can be observed (Figures S31 and S32), which confirms the triplet sensitization of the substrate by the [Zn(*m*-L)_2_] and [Zn(*p*-L)_2_] complexes. Compared to the previously used iridium complex, the Zn^II^ sensitizers employed here have substantially lower triplet energies (2.33 and 2.46 vs 2.64 eV), which might help explain the lower photo-fragmentation yields observed here.^88^ Moreover, iminyl-radicals formed in the photo-fragmentation reaction could potentially poison the catalyst because the Zn^II^ complexes are expected to be considerably more substitution-labile than cyclometalated Ir^III^ compounds.

### Photoinduced Electron Transfer Reactivity

Given the charge-transfer character of the photoactive excited states of [Zn(*m*-L)_2_] and [Zn(*p*-L)_2_], it seemed meaningful to explore their ability to undergo photoinduced electron transfer reactions. Toward this end, we identified 1,2,4,5-tetracyanobenzene (TCB) as a suitable reaction partner because this is a fairly strong electron acceptor featuring a reduction potential of −0.74 V vs SCE in MeCN,^89^ combined with a characteristic spectroscopic signature of its one-electron reduced form (TCB^•–^), which is readily identifiable by transient UV–vis absorption spectroscopy.

**Figure 7:**
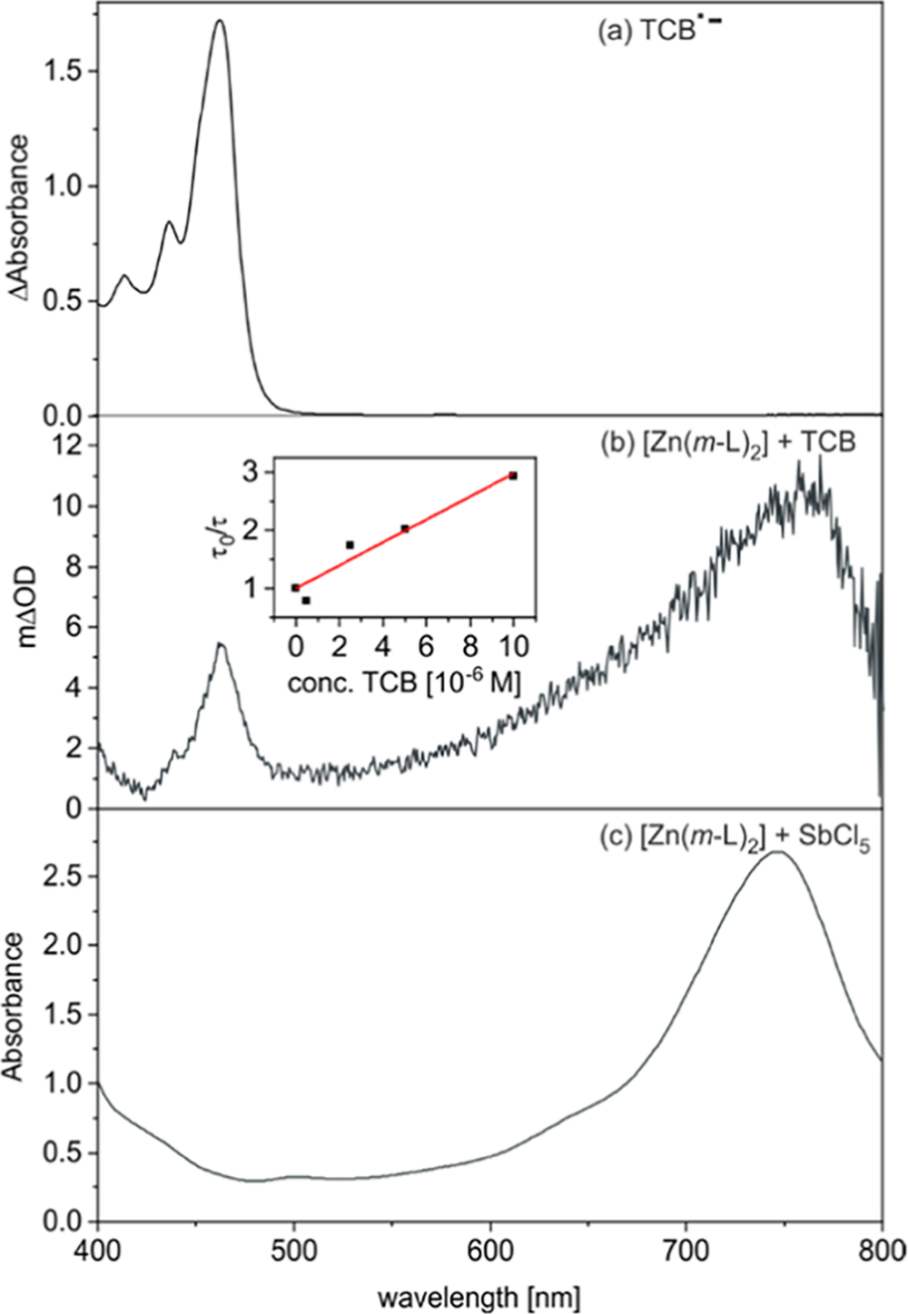
(a) UV–vis difference spectrum obtained upon electrochemical reduction of 1,2,4,5-tetracyanobenzene (TCB, 2.5 mM) in deaerated MeCN in the presence of N(*n*Bu)_4_PF_6_ (0.1 M) as supporting electrolyte, obtained by applying a voltage sufficient to induce one-electron reduction of TCB. (b) Transient absorption difference spectrum of 45 μM [Zn(*m*-L)_2_] and 2.5 mM TCB in deaerated toluene at 293 K, recorded 1 μs after excitation at 420 nm with laser pulses with an energy of 12 mJ. Inset: Stern–Volmer-type plot for the oxidative excited-state quenching of [Zn(*m*-L)_2_] by TCB in deaerated toluene, obtained by monitoring an excited state absorption band at 350 nm (*k*_q_ = 5.3 × 10^9^ M^–1^ s^–1^, Figure S31). (c) Absorption spectrum of [Zn(*m*-L)_2_]^+^ in deaerated CH_2_Cl_2_ at 293 K, obtained after chemical oxidation of a [Zn(*m*-L)_2_] solution (140 μM) with SbCl_5_ (1 equiv).

After selective excitation of [Zn(*m*-L)_2_] at 420 nm in the presence of excess TCB, the resulting UV–vis transient absorption difference spectrum (Figure 7b) is essentially a 1:1 superposition of the spectral signatures of TCB^•–^ (Figure 7a)^90,91^ and [Zn(*m*-L)_2_]^+^ (Figure 7c). The latter resembles very closely the typical spectral signatures of triarylammonium radical cations,^92^ in line with the fact that the primary oxidation of [Zn(*m*-L)_2_] occurs on the triarylamine unit of the chelate ligand. For [Zn(*m*-L)_2_]^+^ the respective absorption band is observed near 750 nm (Figure 7b), whereas for [Zn(*p*-L)_2_]^+^, the analogous absorption band is shifted to near 800 nm (Figure S36c).

In time-correlated single photon counting (TCSPC) experiments, no significant quenching of the ^1^ILCT fluorescence of [Zn(*m*-L)_2_] and [Zn(*p*-L)_2_] by 2.5 mM TCB was observed, suggesting that photoinduced electron transfer to TCB occurs from the long-lived ^3^ILCT states of the Zn^II^ complexes, not from the fluorescent ^1^ILCT states. Indeed, in laser flash photolysis experiments, quenching of the ^3^ILCT states of [Zn(*m*-L)_2_] and [Zn(*p*-L)_2_] by TCB is detectable by monitoring the characteristic absorption bands and ground-state bleaches of the ^3^ILCT excited Zn^II^ complexes at 350 and 390 nm (Figure 5), respectively. By monitoring the ^3^ILCT decays as a function of the TCB concentration (Figures S34 and S35), the Stern–Volmer-type plots in the insets of Figures 7b and S33b were obtained. Linear regression fits to these data gave rate constants of 5.3 × 10^9^ and 6.3 × 10^9^ M^–1^ s^–1^ for photoinduced electron transfer from [Zn(*m*-L)_2_] and [Zn(*p*-L)_2_] to TCB, respectively. These values are relatively close to the diffusion limit in toluene at 293 K (1.1 × 10^10^ M^–1^ s^–1^),^84^ in line with a high driving-force reaction. In cyclic voltammetry, the first oxidation wave appears at 0.32 V vs SCE for [Zn(*m*-L)_2_] (Figure S13) and at 0.80 V vs SCE for [Zn(*p*-L)_2_] (Figure S15). Given the ^3^ILCT energies in Table 1, we estimate excited-state oxidation potentials of −2.01 V vs SCE for [Zn(*m*-L)_2_] (Figure S16) and −1.66 V vs SCE for [Zn(*p*-L)_2_] (Figure S17). Consequently, photoinduced electron transfer to TCB (featuring a reduction potential of −0.74 V vs SCE in MeCN)^89^ is highly exergonic in both cases, which can explain why nearly diffusion-controlled kinetics are observed.

According to this analysis, the two Zn^II^ complexes are both rather strong photoreductants, but [Zn(*m*-L)_2_] is roughly 0.4 V more reducing in its ^3^ILCT excited state than [Zn(*p*-L)_2_]. This finding can be traced back to substantially different ground-state oxidation potentials, which might have their origin in different extents of electronic coupling between the coordinating phenolate units and the triarylamine N-atoms. Specifically, in [Zn(*m*-L)_2_], the anionic phenolate unit stands in *para*-position to the triarylamine N-atom, which might enhance the electron density at the N-atom and could lower the respective oxidation potential in comparison to [Zn(*p*-L)_2_], in which the phenolate unit stands in *meta*-position to the triarylamine N-atom.

### Blue to Ultraviolet Upconversion

**Figure 8:**
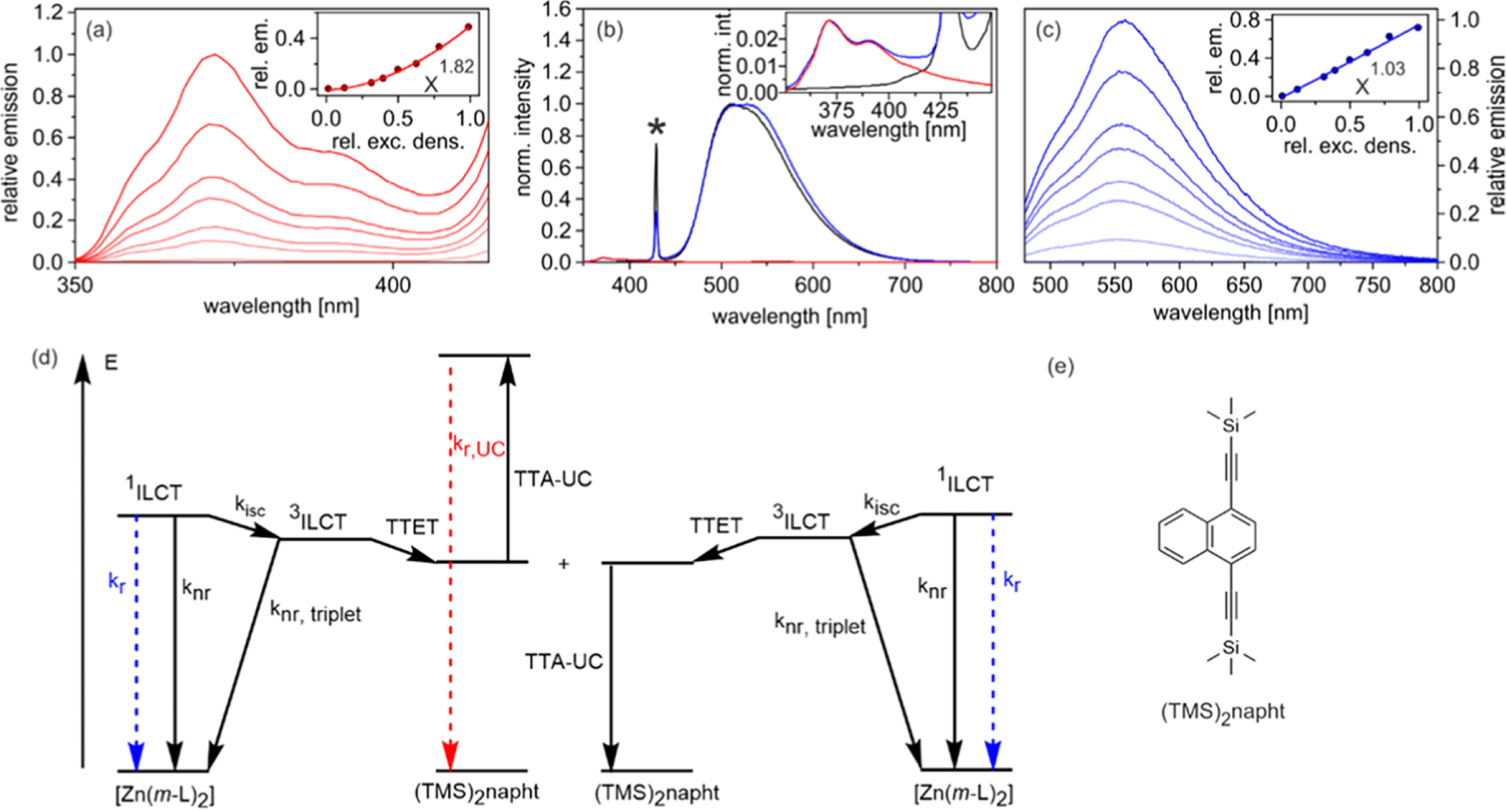
Sensitized triplet–triplet annihilation upconversion (sTTA-UC) using [Zn(*m*-L)_2_] as the sensitizer and 1,4-bis((trimethylsilyl)ethynyl)naphthalene ((TMS)_2_napht) as the annihilator in deaerated toluene. (a) Power dependence of the upconverted emission in a steady-state experiment with a solution containing 2 mM (TMS)_2_napht and 170 μM [Zn(*m*-L)_2_] excited at 447 nm. Inset: Quadratic dependence of the integrated emission intensities from the main plot of (a) on the relative excitation density. (b) Upconversion quantum yield estimation with 2 mM (TMS)_2_napht and 30 μM [Zn(*m*-L)_2_] under pulsed excitation at 430 nm. The blue trace is the prompt sensitizer emission (peaking at 528 nm) as well as the upconverted emission (peaking at 371 nm (seen mostly in the inset), recorded without delay, 100 μs integration time). The red trace represents only the upconverted emission (recorded with 1 μs delay (the prompt sensitizer emission has already decayed completely at that point), 100 μs integration time) of the same sensitizer/annihilator solution. The black trace represents only the prompt (normalized) [Zn(*m*-L)_2_] emission (measured from a 20 μM [Zn(*m*-L)_2_] solution without annihilator, recorded without delay, 100 μs integration time). The asterisk (*) designates stray laser light. Inset: Upconverted emission from the main plot of (b) on an enlarged scale. (c) Power dependence of the prompt emission of [Zn(*m*-L)_2_] in a steady-state experiment with a solution containing 2 mM (TMS)_2_napht and 170 μM [Zn(*m*-L)_2_] excited at 447 nm. Inset: Linear dependence of the data from the main plot of (c) on the relative excitation density. (d) Energy-level diagram of the sensitizer and annihilator, along with a summary of the photophysical processes they undergo in the course of sTTA-UC. (e) Molecular structure of the annihilator 1,4-bis((trimethylsilyl)ethynyl)naphthalene ((TMS)_2_napht).

With their photoactive ^3^ILCT excited states, both [Zn(*m*-L)_2_] and [Zn(*p*-L)_2_] should in principle be able to sensitize triplet–triplet annihilation upconversion, which is an attractive process for the conversion of low-energy input light into higher energy output radiation.^74,93−96^ In particular, upconversion from the visible to the ultraviolet spectral region has received increasing interest lately,^97−102^ and our Zn^II^ complexes with their relatively high ^3^ILCT energies seemed promising for this purpose. Previous upconversion studies with Zn^II^ sensitizers focused on red-to-blue upconversion and on near-infrared sensitization of photochemical reactions.^103−106^ We identified the combination of [Zn(*m*-L)_2_] and (TMS)_2_napht (Figure 8e) as particularly promising for blue to UV upconversion. Unsubstituted naphthalene has a triplet energy of 2.62 eV,^84^ which is too high for both our Zn^II^ complexes (Figure 4). The substitution of polyaromatic hydrocarbon annihilators with protected acetylene units was previously found to lower the energies of the lowest triplet excited states.^107,108^ For example, naphthalene substituted with triisopropylsilyl (TIPS) protected acetylenes in 1- and 4-positions has a triplet energy of 2.10 eV.^98^ The (TMS)_2_napht compound (Figure 8e) differs from that previously investigated annihilator merely by the presence of trimethylsilyl (TMS) instead of TIPS groups, and therefore we anticipate a similar triplet energy of ca. 2.10 eV for (TMS)_2_napht. TTET from the ^3^ILCT excited state of [Zn(*m*-L)_2_] to (TMS)_2_napht is thus expected to be exergonic by roughly 0.2 eV. This specific sensitizer-annihilator combination furthermore seemed beneficial because [Zn(*m*-L)_2_] absorbs less than [Zn(*p*-L)_2_] in the spectral region of the (TMS)_2_napht fluorescence (350–400 nm, Figure S34), which should limit unwanted reabsorption phenomena. In the following, we aimed at a proof-of-principle experiment, to explore the application potential of tetrahedral Zn^II^ complexes for photochemical upconversion.

A solution containing 30 μM [Zn(*m*-L)_2_] and 2 mM (TMS)_2_napht in deaerated toluene was excited at 430 nm with laser pulses of roughly 10 ns duration and 16 mJ energy. At this wavelength, (TMS)_2_napht is essentially transparent and the light is absorbed exclusively by the Zn^II^ complex. Emission spectra were then recorded in a time-gated manner, once immediately after the excitation pulses (blue trace in Figure 8b) and once with a delay of 1 μs (red trace in Figure 8b). The blue trace is dominated by the ^1^ILCT emission of [Zn(*m*-L)_2_] centered around 528 nm, yet between 350 and 400 nm, a weak luminescence band (shown on an enlarged scale in the inset of Figure 8b) is furthermore detectable. That latter emission band also appears in the luminescence spectrum recorded with a time delay (red trace), while the fluorescence of [Zn(*m*-L)_2_] is absent in that case because the ^1^ILCT excited state with its lifetime (τ_singlet_) of 25 ns (Table 1) has completely decayed after 1 μs. The UV emission in the inset of Figure 8b (blue and red traces) is in good agreement with the fluorescence spectrum of (TMS)_2_napht obtained after direct excitation at 350 nm (Figure S34), and consequently, these UV emissions are attributable to the delayed (upconverted) annihilator fluorescence. This delayed fluorescence decays on a microsecond timescale (100 μs time gates were used to record the blue and red traces in Figure 8b) and as such reflects the lifetime of the photoactive triplet excited state of (TMS)_2_napht, which is populated by TTET from the ^3^ILCT state of [Zn(*m*-L)_2_] (Figure 8d).

To estimate the upconversion quantum yield under the conditions of this pulsed laser study, an additional reference experiment was performed, in which we recorded the prompt ^1^ILCT fluorescence of [Zn(*m*-L)_2_] under identical conditions, using a solution containing only the Zn^II^ sensitizer (20 μM) but no annihilator (black trace in Figure 8b). When normalizing the black and blue traces in Figure 8b, we were thus able to determine the integrated ^1^ILCT emission intensity (*I*_fluo_) and the integrated delayed annihilator fluorescence (*I*_UC_) by comparing the black and red traces in Figure 8b (SI page S48). Given a ^1^ILCT fluorescence quantum yield (Φ_fluo_) of 50% (Table 1), this analysis yields an upconversion quantum yield (ϕ_UC_) of 0.73% (relative to a theoretical limit of 50%,^109^ SI page S48). Given the incomplete intersystem crossing in [Zn(*m*-L)_2_] (Φ_isc_ = 13%), the modest upconversion quantum yield is unsurprising. Normalized to the amount of photogenerated triplet excited states on [Zn(*m*-L)_2_], the upconversion quantum yield is 5.6%, which compares favorably to many previously investigated systems.

Excitation power-dependent measurements were performed using a 447 nm continuous-wave (cw) laser and yielded the series of upconversion fluorescence spectra shown in Figure 8a and the prompt ^1^ILCT emission spectra in Figure 8c. When plotting the integrated (normalized) emission intensities as a function of relative excitation density, the expectable quadratic power dependence is obtained for the upconversion emission (inset of Figure 8a, *x*^1.82^), whereas the prompt ^1^ILCT fluorescence shows the anticipated linear power dependence (inset of Figure 8c, *x*^1.03^).

### Photostability of the Zn^II^ Complexes

Many applications require long-term photo-irradiation, and thus it seemed meaningful to explore the photo-robustness of the Zn^II^ complexes from Figure 1 in comparison with [Ru(bpy)_3_]^2+^, a well-known and widely used benchmark compound.^110^ For this purpose, deaerated solutions of [Zn(*m*-L)_2_], [Zn(*p*-L)_2_], and [Ru(bpy)_3_]^2+^ with essentially identical absorbance values (*A*_0_) of 0.05 at 405 nm were prepared (Table S4) and then irradiated at that wavelength with a cw laser providing 0.526 W of output power. Following a previously published method (SI pages S43 and S44),^102,111,112^ we monitored the emission intensity of these three complexes as a function of irradiation time and estimated their photodegradation quantum yields (Φ_decomp_) by considering the time period during which their photoluminescence intensities decreased to 90% of the initial values at *t* = 0 (SI page S43). Given the known (initial) concentrations of the complexes (*c*_0_) and the measurable number of photons absorbed within the above-mentioned time period (the initial absorbance values *A*_0_ are known, and the laser power is known as well), the Φ_decomp_ values in Table 3 were obtained (Table S4). In comparison to [Ru(bpy)_3_]^2+^, the inherent photostability of the two investigated Zn^II^ complexes is an order of magnitude lower, which is perhaps unsurprising given the substantial structural and electronic differences between these compound classes. In contrast to the 3d^10^ Zn^II^ compounds, the octahedral 4d^6^ complex with Ru^II^ benefits from a high ligand field stabilization energy, which decelerates ligand substitution reactions in the electronic ground state, and furthermore might affect the stability in excited states. The ^3^ILCT excited states of the [Zn(*m*-L)_2_] and [Zn(*p*-L)_2_] complexes are considerably longer-lived (τ_triplet_ = 38 and 62 μs, Table 1) than the ^3^MLCT excited state of [Ru(bpy)_3_]^2+^ (τ_triplet_ < 1 μs), which increases the likelihood of photo-decomposition in the Zn^II^ compounds.

**Table 3:** Photodegradation Quantum Yields (Φ_decomp_) of [Zn(*m*-L)_2_], [Zn(*p*-L)_2_], and [Ru(bpy)_3_]^2+^ in Deaerated Solutions at 293 K Irradiated with a 405 nm cw Laser (Power Output of 0.526 W)

complex	ϕ_decomp_ [%]
[Zn(*m*-L)_2_]^a^	0.06
[Zn(*p*-L)_2_]^a^	0.04
[Ru(bpy)_3_]^2+ ^b	0.005

a Determined in deaerated toluene by monitoring the photoluminescence intensity at 510 nm.

b Determined in deaerated H_2_O by monitoring the emission intensity at 550 nm. Further details are in the SI on pages S43 and S44 (Figure S33).

**Figure 9:**
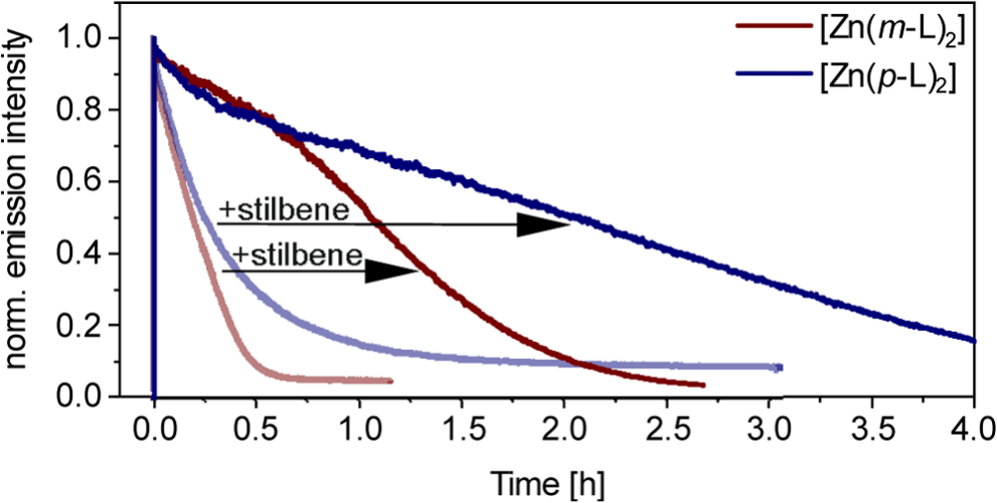
Influence of the addition of *trans*-stilbene on the photostability of [Zn(*m-*L)_2_] and [Zn(*p*-L)_2_] in deaerated toluene at 293 K. Red traces: Normalized intensity of the photoluminescence emitted by [Zn(*m-*L)_2_] at 510 nm in the absence (lighter trace) and presence of 10 mM *trans*-stilbene (darker trace). Blue traces: Analogous data set for [Zn(*p*-L)_2_], with emission intensity monitoring at 490 nm, once without *trans*-stilbene (lighter trace) and once with 10 mM *trans*-stilbene (darker trace). All samples were irradiated with a 415 nm LED (7 W), combined with a 400 nm long-pass filter.

Indeed, experiments in which the long-lived ^3^ILCT excited state of the Zn^II^ compounds is quenched partially by *trans*-stilbene indicate that under conditions of TTET catalysis, [Zn(*m*-L)_2_] and [Zn(*p*-L)_2_] are considerably more photo-robust than in neat solvent without any substrate present (Figure 9). For instance, a neat 3.2 μM solution of [Zn(*p*-L)_2_] in deaerated toluene degraded essentially completely within 1.5 h of irradiation with a 415 nm LED providing 7 W of output power (light blue trace in Figure 9). Upon addition of 10 mM of *trans*-stilbene, it takes more than 4 h to reach a similar level of photodegradation (darker blue trace in Figure 9), and an analogous observation is made when comparing a neat toluene solution of 10 μM [Zn(*m*-L)_2_] with a mixture containing 10 mM *trans*-stilbene (red traces in Figure 9). These observations strongly suggest that photodegradation from the long-lived ^3^ILCT excited states is a major decomposition pathway in the [Zn(*p*-L)_2_] and [Zn(*m*-L)_2_] compounds, as suspected above (Figure 4). Since intersystem crossing is not quantitative in these Zn^II^ complexes (Table 1), it is possible to monitor their photodegradation by following their ^1^ILCT fluorescence intensities, even if the ^3^ILCT is quenched and primarily responsible for the photo-decomposition.

## Conclusions

The application potential of charge-transfer excited states in Zn^II^ complexes has so far remained underexplored in comparison to isoelectronic copper(I) coordination compounds.^39,40^ Here, we demonstrate that intraligand charge-transfer (ILCT) excited states in tetrahedral Zn^II^ complexes can display similar reactivity as metal-to-ligand charge-transfer (MLCT) excited states in four-coordinate Cu^I^ compounds. In the latter, the photoactive MLCT excited states are typically strongly distorted due to the transient formation of a Jahn–Teller susceptible Cu^II^ d^9^ metal species,^78,113^ opening up unwanted nonradiative excited-state relaxation pathways. The ILCT excited states of the Zn^II^ complexes investigated herein do not suffer from this problem because the metal oxidation state remains unchanged. The Zn^II^ cation presumably contributes to mediating intersystem crossing from the initially excited ^1^ILCT to the ^3^ILCT states, from where typical triplet photo-reactivity becomes accessible, including for example the photo-isomerization of olefins and the sensitization of triplet–triplet annihilation upconversion. The ^3^ILCT energies of our Zn^II^ complexes are sufficiently high to allow for upconversion from the blue to the ultraviolet spectral range, and these ^3^ILCT excited states are furthermore very reducing, making these Zn^II^ compounds strong photoreductants even in comparison to some of the well-established precious-metal-based systems.^114^ Since the intersystem crossing is not quantitative, fluorescence from the ^1^ILCT excited state remains observable in our Zn^II^ compounds, in contrast to four-coordinate Cu^I^ complexes with photoactive MLCT states, in which intersystem crossing is usually ultrafast. Thus, our study illustrates some clear analogies and differences between tetrahedral Zn^II^ compounds with photoactive ILCT states and the more well-known four-coordinate Cu^I^ complexes.

The ILCT states of our Zn^II^ complexes complement the different types of photoactive excited states reported recently for first-row and other Earth-abundant transition-metal complexes,^28^ which includes the classical MLCT states for d^6^ complexes (Cr^0^, Mn^I^, Fe^II^, Co^III^),^19,24,25,115−117^ square-planar d^8^ compounds (Ni^II^)^26,118^ and four-coordinate d^10^ complexes (Cu^I^),^3,78^ ligand-to-metal charge-transfer (LMCT) states for Ti^IV^, Zr^IV^,^119,120^ Mn^IV^, Fe^III^ and Co^III^,^121−126^^121−126^ metal-centered (MC) states for V^III^, Cr^III^ and Co^III^,^127−132^ as well as ligand-to-ligand charge-transfer (LLCT) excited states for two-coordinate Cu^I^ complexes.^29−33^ Given these findings, it seems reasonable to conclude that Zn^II^ complexes with charge-transfer and triplet excited states would perhaps deserve greater attention in future studies aiming to discover new photophysics and photochemistry in first-row transition-metal complexes.

## Methods

Chemicals were obtained from commercial suppliers in high purity and were used without further purification. Dry solvents were used as purchased from commercial suppliers or from an Innovative Technology PureSolv micro multiunit solvent purification system.

Nuclear magnetic resonance (NMR) spectroscopy was performed using either a 400 MHz Bruker Avance III spectrometer or a 600 MHz Bruker Avance III spectrometer at 298 K. The latter instrument was equipped with a direct observe 5 mm BBFO smart probe. Chemical shifts δ are given in parts per million (ppm) and referenced to CDCl_3_ (7.26 ppm in ^1^H-NMR and 77.16 ppm in ^13^C-NMR spectroscopy), CD_2_Cl_2_ (5.30 ppm in ^1^H-NMR and 53.84 ppm in ^13^C-NMR spectroscopy), or DMSO-*d*_6_ (2.50 ppm in ^1^H-NMR and 39.52 ppm in ^13^C-NMR spectroscopy).^133^ The multiplicity of the signals is described with the following abbreviations: s (singlet), d (doublet), t (triplet), q (quartet), p (pentet), m (multiplet), and combinations of these abbreviations. The coupling constants *J* are given in hertz (Hz).

High-resolution mass spectroscopy (HRMS) was performed by Dr. Michael Pfeffer on a maxis 4G QTOF EDI spectrometer from Bruker. MALDI-TOF-MS was performed on a Bruker Microflex instrument operating in positive mode. The matrix (DCTB in CH_2_Cl_2_) was evaporated onto the sample plate and then the substrate was evaporated onto the matrix.

Elemental analysis (EA) was performed by Sylvie Mittelheisser on a Vario Micro Cube instrument from Elementar. The amounts of carbon, hydrogen, and nitrogen were determined. All values are given in percentages.

Cyclic voltammetry was either performed in an MBraun glovebox under argon atmosphere using a Versastat4-200 potentiostat from Princeton Applied Research, or under deaerated conditions using a Versastat3-200 potentiostat from Princeton Applied Research.

Photocatalytic reactions were performed in NMR tubes with tube caps from VWR. The irradiation source was a SOLIS-415C high-power LED from ThorLabs with a 380 or 400 nm cutoff filter.

Photostabilities were measured using a SOLIS-415C high-power LED from ThorLabs with a 400 nm cutoff filter or a Roithner Lasertechnik GmbH 405 nm continuous-wave laser with an output power of 526 mW.

All photophysical measurements were carried out at 293 K and the solutions were purged with argon (4.8, PanGas) for at least 5 min using screw cap cuvettes. Steady-state optical absorption and UV–vis spectro-electrochemical measurements were recorded using a Cary 5000 spectrophotometer (Varian). Steady-state luminescence spectra were measured using a Fluorolog-3-22 instrument from Horiba Jobin-Yvon. Transient absorption and time-resolved absorption and emission measurements were performed on an LP920-KS instrument from Edinburgh Instruments. The excitation source was a pulsed Quantel Brilliant B Nd:YAG laser equipped with an optical parameter oscillator (OPO) from OPOTEK or a Nd:YAG laser (Quantel Q-smart 450 mJ, ca. 10 ns pulse width) with a beam expander (BE02-355 from Thorlabs). The transient absorption spectra were detected on an iCCD camera (Andor), while kinetics at a single wavelength were recorded with a photomultiplier tube. Fluorescence lifetimes were measured on a LifeSpec II spectrometer (time-correlated single photon counting technique) from Edinburgh Instruments using picosecond pulsed diode lasers for excitation at 405 nm.

## Supporting Information Available

Materials and methods, synthetic procedures and characterization data, electrochemical data, spectroscopic data, and additional information to photophysical measurements and stability (PDF)
