# Supplementary material for: Zinc(II) Complexes with Triplet Charge-Transfer Excited States Enabling Energy-Transfer Catalysis, Photoinduced Electron Transfer, and Upconversion
Source: JACS Au. 2022 Oct 11;2(10):2367–80. doi: 10.1021/jacsau.2c00442 (PMC9597861; doi:10.1021/jacsau.2c00442)
Supplement: Supplementary file 1 — au2c00442_si_001.pdf [file au2c00442_si_001.pdf]

Supporting Information to

Zinc(II) Complexes with Triplet Charge-Transfer Excited States Enabling Energy-Transfer Catalysis, Photoinduced Electron Transfer, and Upconversion

Jasmin A. Kübler, Björn Pfund, Oliver S. Wenger

Department of Chemistry, University of Basel, St. Johanns-Ring 19, 4056 Basel, Switzerland

TABLE OF CONTENTS

Materials and methods S1

Synthesis and product characterization S3

2-(Benzo[*d*]oxazol-2-yl)-4-bromophenol (*m*-1) S5

2-(2-(Benzyloxy)-5-bromophenyl)benzo[*d*]oxazole (*m*-2) S6

3-(Benzo[*d*]oxazol-2-yl)-4-(benzyloxy)-*N*,*N*-bis(4-methoxyphenyl)aniline (*m*-3) S7

2-(Benzo[*d*]oxazol-2-yl)-4-(bis(4-methoxyphenyl)amino)phenol (*m*-LH) S8

[Zn(2-(benzo[*d*]oxazol-2-yl)-4-(bis(4-methoxyphenyl)amino)phenyl)$_2$] ([Zn(*m*-L)$_2$]) S9

2-(Benzo[*d*]oxazol-2-yl)-5-bromophenol (*p*-1) S10

2-(2-(Benzyloxy)-4-bromophenyl)benzo[*d*]oxazole (*p*-2) S11

4-(Benzo[*d*]oxazol-2-yl)-3-(benzyloxy)-*N*,*N*-bis(4-methoxyphenyl)aniline (*p*-3) S12

2-(Benzo[*d*]oxazol-2-yl)-5-(bis(4-methoxyphenyl)amino)phenol (*p*-LH) S13

[Zn(2-(benzo[d]oxazol-2-yl)-5-(bis(4-methoxyphenyl)amino)phenol)$_2$] ([Zn(p-4)$_2$]) S14

1,4-Bis((trimethylsilyl)ethynyl)naphthalene ((TMS)$_2$napht) S15

Chirality of the complexes S16

Photophysical measurements S17

Absorption and emission S17

Transient absorption S19

Luminescence and intersystem crossing quantum yields and lifetimes S20

Kinetics S23

Cyclic voltammetry S27

Photocatalysis S30

Photoisomerization of trans-stilbene S31

Photoisomerization of methyl *trans*-cinnamate S34

Photocatalytic decarboxylation S37

Photostability S43

Photoinduced electron transfer to 1,2,4,5-tetracyanobenzene S45

Upconversion S48

NMR spectra S50

Mass spectra S52

MALDI-TOF MS S52

TD-DFT calculations S55

Ground state geometry of [Zn(*p*-L)$_2$] in vacuum S59

Triplet state geometry of [Zn(*p*-L)$_2$] in vacuum S61

Ground state geometry of [Zn(*m*-L)$_2$] in vacuum S63

Triplet state geometry of [Zn(*m*-L)$_2$] in vacuum S65

References S67

MATERIALS AND METHODS

Chemicals were obtained from commercial suppliers in high purity and were used without further purification. Dry solvents were used as purchased from commercial suppliers or from an Innovative Technology PureSolv micro multi-unit solvent purification system.

Nuclear magnetic resonance (NMR) spectroscopy was performed using either a 400 MHz Bruker Avance III spectrometer or a 600 MHz Bruker Avance III spectrometer at 298 K. The latter instrument was equipped with a direct observe 5-mm BBFO smart probe. Chemical shifts δ are given in ppm (parts per million) and referenced to $CDCl_3$ (7.26 ppm in ^{1}H-NMR and 77.16 ppm in ^{13}C-NMR spectroscopy), CD_2Cl_2 (5.30 ppm in ^{1}H-NMR and 53.84 ppm in ^{13}C-NMR spectroscopy) or DMSO-d_6 (2.50 ppm in ^{1}H-NMR and 39.52 ppm in ^{13}C-NMR spectroscopy).[1] The multiplicity of the signals is described with the following abbreviations: s (singlet), d (doublet), t (triplet), q (quartet), p (pentet), m (multiplet) and combinations of these abbreviations. The coupling constants J are given in Hertz (Hz).

High resolution mass spectroscopy (HRMS) was performed by Dr. Michael Pfeffer on a maxis 4G QTOF EDI spectrometer from Bruker. MALDI-TOF-MS was performed on a Bruker microflex instrument operating in positive mode. The matrix (DCTB in CH_2Cl_2) was evaporated onto the sample plate and then the substrate was evaporated onto the matrix.

Elemental analysis (EA) was performed by Sylvie Mittelheisser on a Vario Micro Cube instrument from Elementar. The amounts of carbon, hydrogen and nitrogen were determined. All values are given in percentages.

Cyclic voltammetry was either performed in an MBraun glovebox under argon atmosphere using a Versastat4-200 potentiostat from Princeton Applied Research, or under deaerated conditions using a Versastat3-200 potentiostat from Princeton Applied Research.

Photocatalytic reactions were performed in NMR tubes with tube caps from VWR. The irradiation source was a SOLIS-415C high-power LED from ThorLabs with a 380 or 400 nm cutoff filter.

Photostabilities were measured using a SOLIS-415C high-power LED from ThorLabs with a 400 nm cutoff filter or a Roithner Lasertechnik GmbH 405 nm continuous wave laser with an output power of 526 mW.

All photophysical measurements were carried out at 293 K and the solutions were purged with argon (4.8, PanGas) for at least 5 minutes using screw cap cuvettes. Steady-state optical absorption and UV-Vis spectro-electrochemical measurements were recorded using a Cary 5000

spectrophotometer (Varian). Steady-state luminescence spectra were measured using a Fluorolog-3-22 instrument from Horiba Jobin-Yvon. Transient absorption and time-resolved absorption and emission measurements were performed on a LP920-KS instrument from Edinburgh Instruments. The excitation source was a pulsed Quantel Brilliant b ND:YAG laser equipped with an optical parameter oscillator (OPO) from OPOTEK or a Nd:YAG laser (Quantel Q-smart 450 mJ, ca. 10 ns pulse width) with a beam expander (BE02-355 from Thorlabs). The transient absorption spectra were detected on an iCCD camera (Andor), while kinetics at a single wavelength were recorded with a photomultiplier tube. Fluorescence lifetimes were measured on a LifeSpec II spectrometer (time-correlated single photon counting technique) from Edinburgh Instruments using picosecond pulsed diode lasers for excitation at 405 nm.

SYNTHESIS AND PRODUCT CHARACTERIZATION

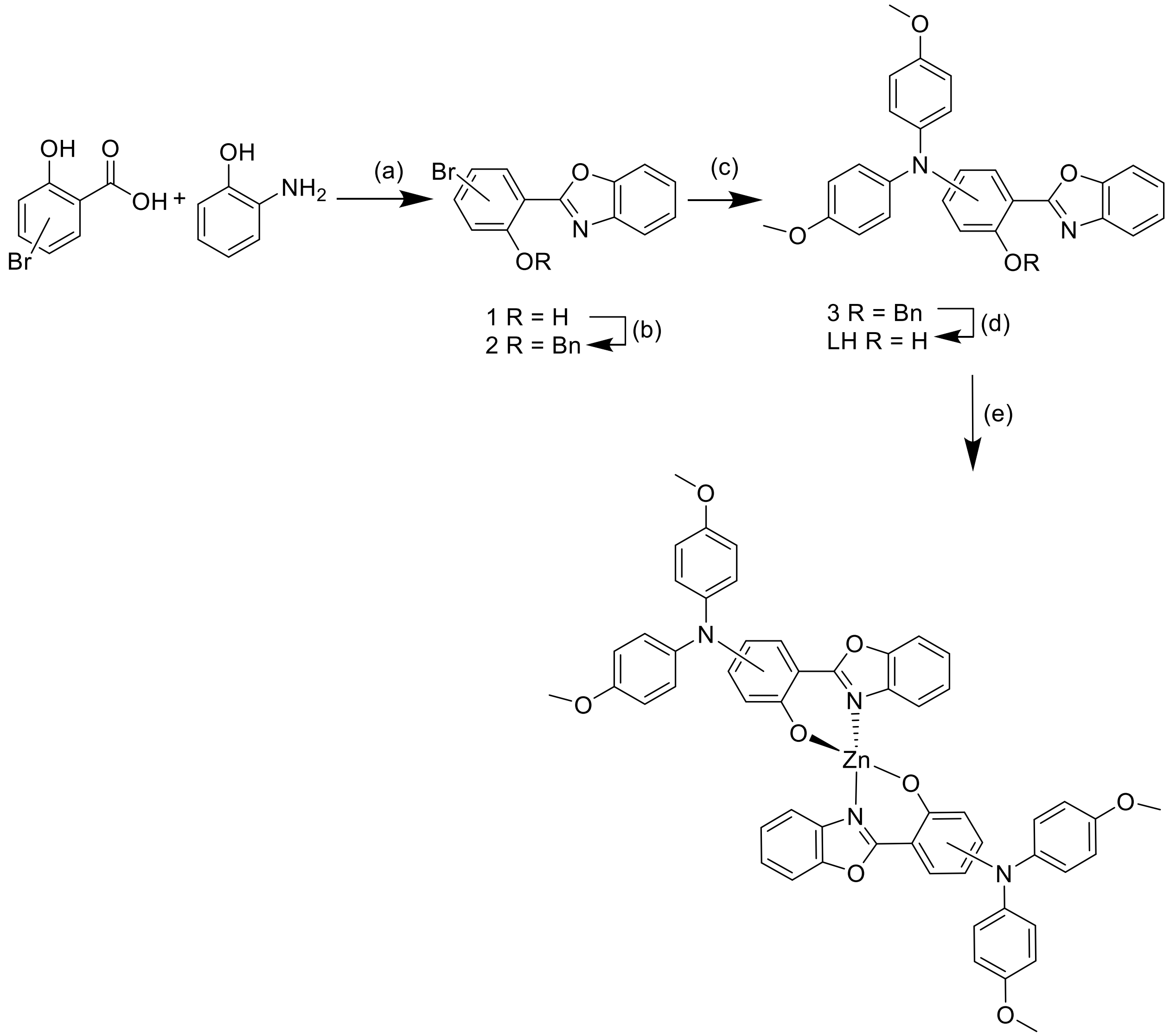

[Zn(*m*-L)$_2$] and [Zn(*p*-L)$_2$]

Scheme S1: Synthesis of [Zn(*m*-L)$_2$] and [Zn(*p*-L)$_2$] (a) polyphosphoric acid, 180 °C; (b) Cs_2CO_3, benzyl bromide, MeCN, reflux; (c) Pd(dba)$_2$ or Pd(OAc)$_2$, bis(4-methoxyphenyl)amine, K_2CO_3 or KO^{t}Bu, Sphos or (^{t}Bu)$_3$P, toluene, 90 or 111 °C; (d) H_2, Pd/C, CH_2Cl_2, RT; (e) Zn(OAc)$_2$, THF or toluene, 66 °C.

The ligands *m*-LH and *p*-LH were synthesized starting with a previously published condensation reaction of the respective brominated salicylic acid and 2-aminophenol in polyphosphoric acid,[2] and then following a known synthesis strategy as displayed in Scheme S1.[3]. The Buchwald-Hartwig coupling with bis(4-methoxyphenyl)amine was specifically optimized for the reaction

partner *m*-2. The final deprotection of the phenolic oxygen was performed in CH_2Cl_2 using palladium on activated charcoal under a hydrogen atmosphere, which gave the ligands *m*-LH and *p*-LH in excellent yields. Complexation was achieved by refluxing two equivalents of *m*-LH or *p*-LH with $Zn(OAc)_2$ in tetrahydrofuran (THF) or toluene overnight. After filtration, the ^{1}H-NMR spectra of the complexes exhibited broad and ill-defined signals. In addition to the anticipated signals for [Zn(*m*-L)$_2$] or [Zn(*p*-L)$_2$] at m/z = 938 the MALDI-TOF-MS data furthermore indicated the formation of dinuclear [Zn$_2$(*m*-L)$_3$] or [Zn$_2$(*p*-L)$_3$] complexes with m/z = 1443. The yellow powders were heated to 220 °C under vacuum overnight in a sublimation apparatus. On the cooling finger sublimated ligand *m*-LH or *p*-LH was collected, while the remaining powder in the flask was again subjected to NMR and MS analysis. The signals for the dinculear [Zn$_2$(*m*-L)$_3$] or [Zn$_2$(*p*-L)$_3$] complexes in the MALDI-TOF-MS were no longer present and well-defined ^{1}H-NMR signals for [Zn(*m*-L)$_2$] or [Zn(*p*-L)$_2$] were observed. The mononuclear target complexes were furthermore characterized by elemental analysis and HR-ESI-MS.

2-(BENZO[*D*]OXAZOL-2-YL)-4-BROMOPHENOL (*M*-1)

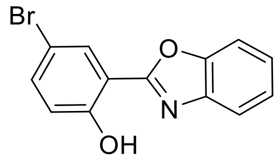

5-Bromo-2-hydroxybenzoic acid (3.00 g, 13.8 mmol, 1.0 eq.) and 2-aminophenol (1.51 g, 13.8 mmol, 1.0 eq.) were suspended in polyphosphoric acid (8.0 mL). The reaction mixture was stirred at 180 °C overnight before it was allowed to cool to RT and poured into ice-water. The precipitate was collected by filtration, washed with water and dried *in vacuo* overnight, yielding the product (*m*-1, 3.18 g, 11.0 mmol, 80%) as a pink solid, which was used for the next reaction without further purification. Analytical data matches the literature.[3]

^{1}H-NMR ($CDCl_3$, 298 K, 400 MHz) δ [ppm]: 11.29 (s, 1H), 8.15 (d, *J* = 2.5 Hz, 1H), 7.78 – 7.72 (m, 1H), 7.66 – 7.60 (m, 1H), 7.52 (dd, *J* = 8.8, 2.5 Hz, 1H), 7.44 – 7.39 (m, 2H), 7.02 (d, *J* = 8.9 Hz, 1H).

2-(2-(Benzyloxy)-5-bromophenyl)benzo[*d*]oxazole (*m*-2)

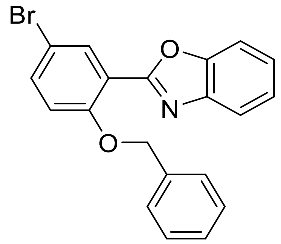

This reaction was adapted from a previously published procedure.[3] 2-(Benzo[*d*]oxazol-2-yl)-4-bromophenol (*m*-1, 1.51 g, 5.20 mmol, 1.0 eq.) and Cs_2CO_3 (1.00 g, 5.18 mmol, 1.0 eq.) were dissolved in MeCN (33 mL). Benzyl bromide (0.62 mL, 5.22 mmol, 1.0 eq.) was added and the reaction mixture was stirred at 80 °C for 2.5 h before it was allowed to cool to RT and filtered. The filtrate was diluted with CH_2Cl_2. The MeCN/CH_2Cl_2 mixture was washed with an aq. solution of NaOH (0.2 M) and brine, and then dried over anhydrous Na_2SO_4. The crude product was purified by column chromatography (SiO_2, CH_2Cl_2 : EtOH 50:1) yielding the product (*m*-2, 1.58 g, 4.16 mmol, 80%) as a pink solid. Analytical data matches the literature.[3]

^{1}H-NMR ($CDCl_3$, 298 K, 400 MHz) δ [ppm]: 8.32 (d, J = 2.5 Hz, 1H), 7.86 – 7.77 (m, 1H), 7.61 – 7.52 (m, 4H), 7.43 – 7.35 (m, 4H), 7.35 – 7.30 (m, 1H), 7.00 (d, J = 8.9 Hz, 1H), 5.30 (s, 2H).

3-(Benzo[*d*]oxazol-2-yl)-4-(benzyloxy)-*N*,*N*-bis(4-methoxyphenyl)aniline (*m*-3)

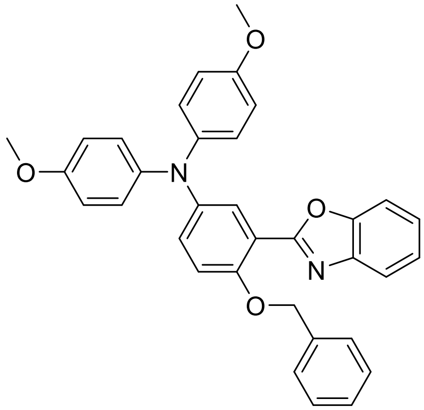

2-(2-(Benzyloxy)-5-bromophenyl)benzo[*d*]oxazole (*m*-2, 743 mg, 1.95 mmol, 1.0 eq.), bis(dibenzylideneacetone)palladium(0) (113 mg, 197 μmol, 10 mol-%), 2-dicyclohexylphosphino-2′,6′-dimethoxybiphenyl (80.1 mg, 195 μmol, 10 mol-%), bis(4-methoxyphenyl)amine (678 mg, 2.96 mmol, 1.5 eq.), and KO^{t}Bu (659 mg, 5.88 mmol, 3.0 eq.) were set under an inert gas atmosphere. Deaerated toluene (30 mL) was added and the reaction mixture was heated to 90 °C for 20 h. H_2O and CH_2Cl_2 were added, the phases were separated and the aqueous phase was extracted with CH_2Cl_2. The combined organic phases were dried over anhydrous Na_2SO_4, and the solvent was removed under reduced pressure. The crude product was purified by column chromatography (SiO_2, CH_2Cl_2) yielding the product (*m*-3, 372 mg, 703 μmol, 36%) as a yellow oil.

^{1}H-NMR ($CDCl_3$, 298 K, 400 MHz) *δ* [ppm]: 7.81 (d, *J* = 2.8 Hz, 1H), 7.79 – 7.74 (m, 1H), 7.59 – 7.51 (m, 3H), 7.41 – 7.28 (m, 5H), 7.10 (dd, *J* = 8.9, 2.9 Hz, 1H), 7.05 – 6.96 (m, 5H), 6.84 – 6.79 (m, 4H), 5.24 (s, 2H), 3.79 (s, 6H).

^{13}C-NMR ($CDCl_3$, 298 K, 151 MHz) *δ* [ppm]: 161.9, 155.5, 152.6, 150.8, 142.9, 142.1, 141.5, 137.2, 128.6, 127.9, 127.2, 127.2, 125.5, 125.4, 125.0, 124.4, 120.2, 118.1, 115.9, 114.9, 110.6, 71.8, 55.6.

HR-MS (ESI): *m/z* = calc. for $[M+H]^+$: 529.2127 ; meas.: 529.2119.

2-(Benzo[*d*]oxazol-2-yl)-4-(bis(4-methoxyphenyl)amino)phenol (*m*-LH)

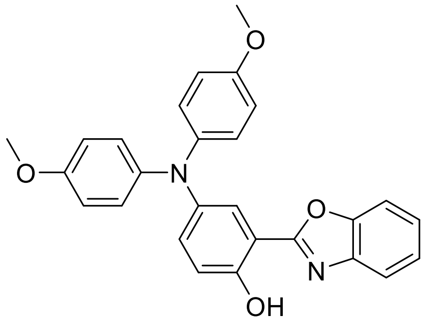

3-(Benzo[*d*]oxazol-2-yl)-4-(benzyloxy)-*N*,*N*-bis(4-methoxyphenyl)aniline (*m*-3, 103 mg, 195 mmol, 1.0 eq.) was dissolved in CH_2Cl_2 (1.0 mL), and Pd on activated charcoal (21 mg, 20 wt.-% Pd) was added. The reaction mixture was stirred at RT under a hydrogen atmosphere (8 bar) for 3 h. The suspension was filtered over celite and the solvent was removed under reduced pressure yielding the product *m*-LH as a yellow solid in essentially quantitative yield.

^{1}H-NMR ($CDCl_3$, 298 K, 400 MHz) δ [ppm]: 7.74 – 7.69 (m, 2 H), 7.57 – 7.52 (m, 1 H), 7.39 – 7.33 (m, 2 H), 7.16 (dd, *J* = 8.9, 2.8 Hz, 1 H), 7.04 – 6.99 (m, 5 H), 6.85 – 6.80 (m, 4 H), 3.80 (s, 6 H).

^{13}C-NMR ($CDCl_3$, 298 K, 151 MHz) δ [ppm]: 162.8, 155.3, 154.3, 149.2, 141.8, 141.2, 140.2, 130.1, 125.5, 125.1, 125.1, 121.4, 119.3, 118.4, 114.8, 111.0, 110.9, 55.7.

HR-MS (ESI): *m/z* = calc. for $[M+H]^+$: 439.1652 ; meas.: 439.1653.

Anal. Calcd for $C_{27}H_{22}N_2O_4 \cdot 0.5\,H_2O$: C, 72.47; H, 5.18; N, 6.26. Found: C, 72.22; H, 5.37; N, 6.27.

[Zn(2-(benzo[*d*]oxazol-2-yl)-4-(bis(4-methoxyphenyl)amino)phenyl)$_2$] ([Zn(*m*-L)$_2$])

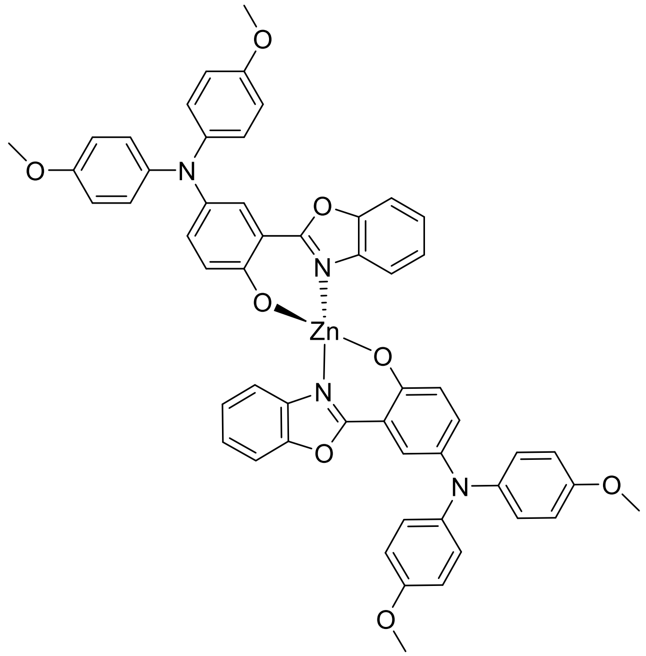

A solution of $Zn(OAc)_2$ (10.5 mg, 57.2 µmol, 1.0 eq.) in dry THF (0.4 mL) was added dropwise to a solution of 2-(benzo[*d*]oxazol-2-yl)-4-(bis(4-methoxyphenyl)amino)phenol (*m*-LH, 50.0 mg, 114 mmol, 2.0 eq.) in dry THF (1.5 mL). The reaction mixture was stirred at 66 °C overnight. The precipitate was collected by filtration, washed with H_2O and EtOH, and then dried *in vacuo* in a stove, yielding the product ([Zn(*p*-L)$_2$], 27.2 mg, 28.9 µmol, 51%) as a yellow solid. The ^{1}H-NMR spectrum was measured immediately after heating the complex to 220 °C under vacuum overnight. This step was of key importance to obtain the mononuclear target complex (see comments below Scheme S1).

^{1}H-NMR ($CDCl_3$, 298 K, 400 MHz) δ [ppm]: 7.78 – 7.68 (m, 2H), 7.54 – 7.45 (m, 2H), 7.43 – 7.34 (m, 2H), 7.32 – 7.28 (m, 2H), 7.23 – 7.13 (m, 4H), 7.07 – 6.99 (m, 8H), 6.99 – 6.92 (m, 2H), 6.87 – 6.78 (m, 8H), 3.80 (s, 12H).

MS (MALDI): m/z for $[M]^+$ calc.: 938.229; meas.: 938.237.

Anal. Calcd for $C_{54}H_{42}N_4O_8Zn\cdot 2\ C_4H_8O\cdot 2\ CH_2Cl_2$: C, 61.28; H, 4.98; N, 4.47. Found: C, 61.47; H, 4.87; N, 4.23.

For both complexes only minor signals in the HR-ESI-MS spectra were observed (with the expected isotope pattern and main peak at m/z=937), while the major peaks corresponded to the ligands *m*-LH and *p*-LH, indicating that the complexes are unstable upon electrospray ionization.

2-(BENZO[*D*]OXAZOL-2-YL)-5-BROMOPHENOL (*P*-1)

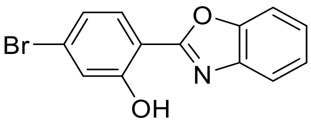

This reaction was adapted from a previously published procedure.[3] 4-Bromo-2-hydroxybenzoic acid (3.00 g, 13.8 mmol, 1.0 eq.) and 2-aminophenol (1.51 g, 13.8 mmol, 1.0 eq.) were suspended in polyphosphoric acid (10 mL) and stirred at 180 °C for 18 h. The reaction mixture was allowed to cool to RT and poured into ice-cold water. The precipitate was collected by filtration, washed with water and dried *in vacuo* yielding the product (*p*-1, 3.05 g, 10.5 mmol, 76%) as a green solid, which was used for the next reaction without further purification. Analytical data matches the literature.[3]

^{1}H-NMR ($CDCl_3$, 298 K, 400 MHz) δ [ppm]: 7.88 (d, J = 8.4 Hz, 1 H), 7.77 – 7.71 (m, 1 H), 7.64 – 7.58 (m, 1 H), 7.44 – 7.37 (m, 2 H), 7.33 (d, J = 1.9 Hz, 1 H), 7.16 (dd, J = 8.4, 1.9 Hz, 1 H).

2-(2-(Benzyloxy)-4-bromophenyl)benzo[*d*]oxazole (*p*-2)

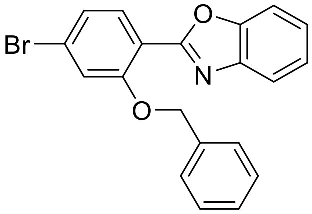

This reaction followed a known procedure.[3] 2-(Benzo[*d*]oxazol-2-yl)-5-bromophenol (*p*-1, 1.51 g, 5.19 mmol, 1.0 eq.) and Cs_2CO_3 (998 mg, 5.17 mmol, 1.0 eq.) were added to MeCN (25 mL) under an inert gas atmosphere. Benzyl bromide (886 mg, 5.18 mmol, 1.0 eq.) was added and the reaction mixture was heated to 80 °C for 2.5 h. The suspension was allowed to cool to RT and filtered. The filtrate was diluted with CH_2Cl_2 and washed with aq. NaOH (0.2 M) and brine. The organic layer was dried over anhydrous Na_2SO_4. The crude product was recrystallized from pentane yielding the product (*p*-2, 930 mg, 2.45 mmol, 47%) as an orange solid. Analytical data matches the literature.[3]

^{1}H-NMR ($CDCl_3$, 298 K, 400 MHz) δ [ppm]: 8.06 (d, J = 8.3 Hz, 1 H), 7.82 (ddd, J = 5.8, 3.2, 0.7 Hz, 1 H), 7.61 – 7.55 (m, 3 H), 7.45 – 7.27 (m, 7 H), 5.31 (s, 2 H).

4-(BENZO[D]OXAZOL-2-YL)-3-(BENZYLOXY)-*N*,*N*-BIS(4-METHOXYPHENYL)ANILINE (*P*-3)

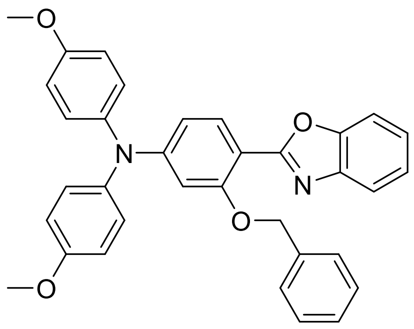

This reaction was adapted from a previously published procedure.[3] 2-(2-(Benzyloxy)-4-bromophenyl)benzo[*d*]oxazole (*p*-2, 400 mg, 1.05 mmol, 1.0 eq.), K_2CO_3 (486 mg, 3.51 mmol, 3.3 eq.), bis(4-methoxyphenyl)amine (302 mg, 1.32 mmol, 1.3 eq.) and $Pd(OAc)_2$ (23.6 mg, 0.105 mmol, 0.1 eq.) were suspended in dry toluene (2.0 mL) under an inert gas atmosphere. $(^tBu)_3P$ (0.12 mL, 1.0 M in toluene, 0.120 mmol, 0.11 eq.) was added. The reaction mixture was refluxed overnight before it was allowed to cool to RT. CH_2Cl_2 and H_2O were added. The phases were separated and the aqueous phase was extracted with CH_2Cl_2. The combined organic phases were washed with brine and dried over $NaSO_4$. The solvent was removed under reduced pressure and the resulting crude product was purified by column chromatography (SiO_2, CH_2Cl_2 : petroleum ether 5:1 → neat CH_2Cl_2) yielding the product (*p*-3, 284 mg, 0.537 mmol, 51%) as a yellow solid.

^{1}H-NMR ($CDCl_3$, 298 K, 400 MHz) δ [ppm]: 7.96 (d, *J* = 9.3 Hz, 1 H), 7.76 (d, *J* = 7.3 Hz, 1 H), 7.55 – 7.49 (m, 1 H), 7.47 – 7.41 (m, 2 H), 7.39 – 7.27 (m, 5 H), 7.12 – 7.04 (m, 4 H), 6.90 – 6.82 (m, 4 H), 6.51 (dq, *J* = 4.2, 2.2 Hz, 2 H), 5.09 (s, 2 H), 3.83 (s, 6 H).

^{13}C-NMR ($CDCl_3$, 298 K, 151 MHz) δ [ppm]:162.6, 158.4, 156.8, 152.7, 150.4, 142.4, 139.3, 136.9, 131.9, 128.4, 127.8, 127.6, 126.9, 124.0, 119.5, 116.4, 114.2, 111.1, 110.1, 107.6, 103.5, 70.4, 55.5.

HR-MS (ESI): *m/z* = calc. for $[M+H]^+$: 529.2127 ; meas.: 529.2131.

2-(Benzo[d]oxazol-2-yl)-5-(bis(4-methoxyphenyl)amino)phenol (p-LH)

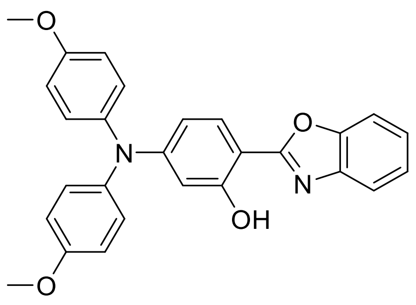

4-(Benzo[d]oxazol-2-yl)-3-(benzyloxy)-*N*,*N*-bis(4-methoxyphenyl)aniline (*p*-3, 104 mg, 197 µmol, 1.0 eq.) was dissolved in CH_2Cl_2 (1.0 mL). Pd on activated charcoal (38 mg, 20 wt.-% Pd) was added and the reaction mixture was stirred at RT under a hydrogen atmosphere (7 bar) for 3 h. The suspension was filtered over celite and the solvent was removed under reduced pressure yielding the product (*p*-LH, 85.2 mg, 194 µmol, 99%) as a yellow solid, which was used for the next reaction without further purification.

^{1}H-NMR (DMSO-d_6, 298 K, 400 MHz) δ [ppm]: 11.16 (s, 1 H), 7.83 – 7.67 (m, 3 H), 7.41 – 7.37 (m, 2 H), 7.25 – 7.20 (m, 4 H), 7.03 – 6.98 (m, 4 H), 6.31 (dd, J = 8.9, 2.3 Hz, 1 H), 6.15 (d, J = 2.3 Hz, 1 H), 3.78 (s, 6 H).

^{1}H-NMR (CD_2Cl_2, 298 K, 400 MHz) δ [ppm]: 11.32 (s, 1H), 7.74 (d, J = 8.8 Hz, 1H), 7.66 – 7.61 (m, 1H), 7.58 – 7.54 (m, 1H), 7.38 – 7.28 (m, 2H), 7.19 – 7.13 (m, 4H), 6.93 – 6.88 (m, 4H), 6.44 (dd, J = 8.8, 2.4 Hz, 1H), 6.39 (d, J = 2.3 Hz, 1H), 3.81 (s, 6H).

^{13}C-NMR ($CDCl_3$, 298 K, 101 MHz) δ [ppm]: 172.9, 163.1, 162.3, 159.6, 156.8, 153.3, 148.7, 140.1, 138.9, 127.9, 127.4, 124.4, 124.2, 118.1, 114.7, 110.1, 109.7, 104.2, 101.5, 55.2.

HR-MS (ESI): *m/z* = calc. for $[M+H]^+$: 439.1652 ; meas.: 439.1651.

[Zn(2-(benzo[d]oxazol-2-yl)-5-(bis(4-methoxyphenyl)amino)phenol)$_2$] ([Zn(p-4)$_2$])

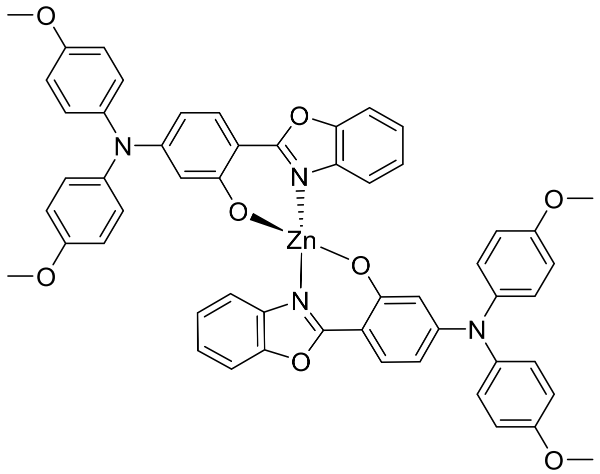

A solution of 2-(benzo[*d*]oxazol-2-yl)-5-(bis(4-methoxyphenyl)amino)phenol (*p*-LH, 49.8 mg, 114 μmol, 2.0 eq.) was dissolved in dry toluene (1.5 mL) and added to a solution of $Zn(OAc)_2$ (10.3 mg, 56.1 μmol, 1.0 eq.) in dry THF yielding a mixture of the desired [Zn(*p*-L)$_2$] and [Zn$_2$(*p*-L)$_3$] as a yellow solid in essentially quantitative yield. The ^{1}H-NMR spectrum was measured immediately after heating the crude product to 220 °C under vacuum overnight. This step was of key importance to obtain the mononuclear target complex (see comments below Scheme S1).

^{1}H-NMR (CD_2Cl_2, 298 K, 400 MHz) δ [ppm]: 7.77 (d, J = 8.9 Hz, 2H), 7.50 (d, J = 8.1 Hz, 2H), 7.28 – 7.19 (m, 4H), 7.18 – 7.05 (m, 10H), 6.86 (d, J = 8.5 Hz, 8H), 6.28 – 6.18 (m, 4H), 3.79 (s, 12H).

MS (MALDI): m/z for $[M]^+$ calc.: 938.229; meas.: 937.532.

Anal. Calcd for $C_{54}H_{42}N_4O_8Zn \cdot 1.5\ C_4H_8O$: C, 68.73; H, 5.19; N, 5.34. Found: C, 68.62; H, 4.93; N, 5.13.

For both complexes only minor signals in the HR-ESI-MS spectra were observed (with expected isotope pattern and main peak at m/z=937), while the major peaks correspond to the ligands *m*-HL and *p*-HL, indicating that the complexes are unstable upon electrospray ionization.

1,4-BIS((TRIMETHYLSILYL)ETHYNYL)NAPHTHALENE ((TMS)2NAPHT)

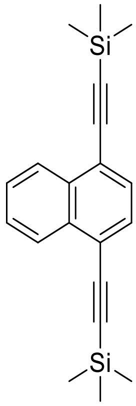

This reaction was adapted from a previously published procedure.[4]

A solution of (trimethylsilyl)acetylene (0.70 mL, 5.06 mmol, 2.5 eq.) in degassed THF (12 mL) was added to a mixture of 1,4-dibromonaphthalene (574 mg, 2.01 mmol, 1.0 eq.), CuI (39.7 mg, 0.209 mmol, 0.10 eq.), and $Pd(PPh_3)_4$ (113 mg, 98.2 mmol, 0.049 eq.) under inert atmosphere. Et_3N (12.0 mL, 86.1 mmol, 43 eq.) was added. The reaction mixture was heated to reflux for 24 h before it was allowed to cool to RT. The suspension was filtered over celite. The solvent of the filtrate was removed under reduced pressure and the crude product purified by column chromatography (SiO2, petroleum ether), yielding the product (596 mg, 1.86 mmol, 93%) as a pale yellow solid. Analytical data matches the literature.[4] **^{1}H-NMR** ($CDCl_3$, 298 K, 400 MHz) δ [ppm]: 8.33 (dd, *J* = 6.2, 3.3 Hz, 2H), 7.66 – 7.56 (m, 4H), 0.33 (s, 18H).

^{13}C NMR ($CDCl_3$, 126 MHz, $CDCl_3$) δ [ppm]: 133.22, 130.12, 127.40, 126.70, 121.68, 102.94, 101.53, 0.19.

CHIRALITY OF THE COMPLEXES

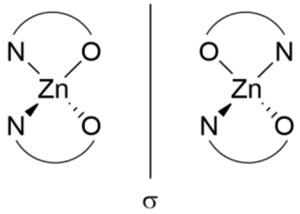

Figure S1: Schematic representation of the two isomers of [Zn(*m*-L)$_2$] and [Zn(*p*-L)$_2$] with a mirror axis σ.

PHOTOPHYSICAL MEASUREMENTS

ABSORPTION AND EMISSION

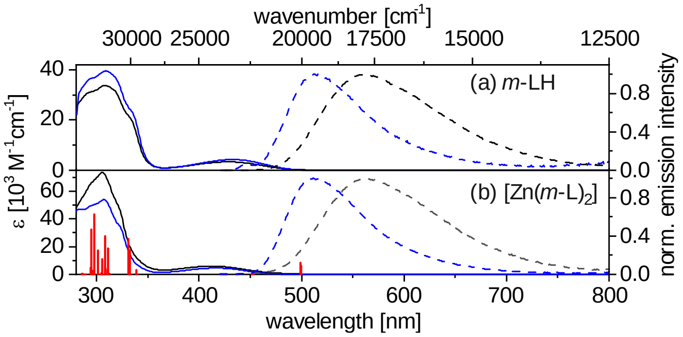

Figure S2: UV-Vis absorption (solid lines) and normalized emission spectra (dashed lines) of (a) *m*-LH (excited at 415 nm) and b) [Zn(*m*-L)$_2$] (excited at 419 nm) in deaerated toluene (blue) and deaerated CH_2Cl_2 (black) at 293 K. The relative oscillator strengths of the first 60 vertical transitions (S_1-S_{60}) were calculated by TD-DFT. Vertical transitions with an energy below 4.43 eV (corresponding to 280 nm) are displayed as horizontal red bars.

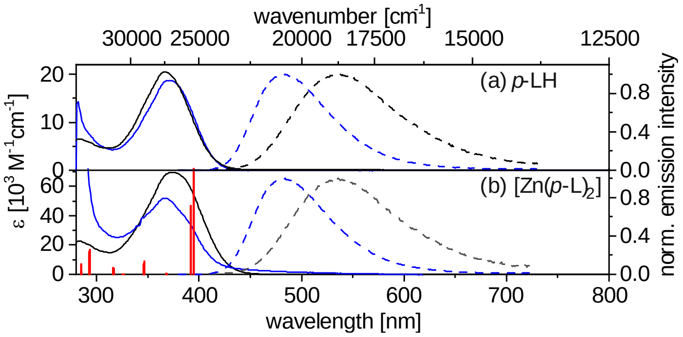

Figure S3: UV-Vis absorption (solid lines) and normalized emission spectra (dashed lines) of (a) *p*-LH (excited at 370 nm) and (b) [Zn(*p*-L)$_2$] (excited at 370 nm) in deaerated toluene (blue) and deaerated CH_2Cl_2 (black) at 293 K. The relative oscillator strengths of the first 60 vertical transitions (S_1-S_{60}) were calculated by TD-DFT. Vertical transitions with an energy below 4.43 eV (corresponding to 280 nm) are displayed as horizontal red bars.

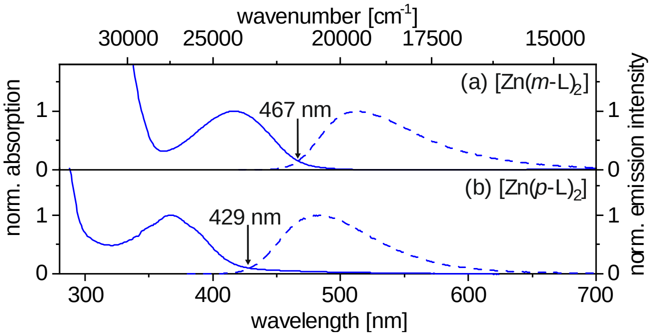

Figure S4: Normalized absorption (solid lines) and normalized emission intensity (dashed lines) of (a) [Zn(*m*-L)$_2$] (excited at 419 nm) and (b) [Zn(*p*-L)$_2$] (excited at 370 nm) in deaerated toluene at 293 K.

Transient absorption

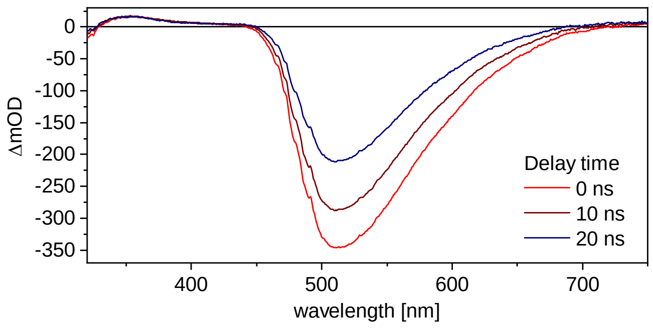

Figure S5: Transient absorption spectra of [Zn(m-L)$_2$] in deaerated toluene at 293 K, recorded at different time delays as indicated by the legend. The sample was excited at 424 nm with laser pulse energies of 11 mJ, and the integration time was 200 ns after the indicated delay times.

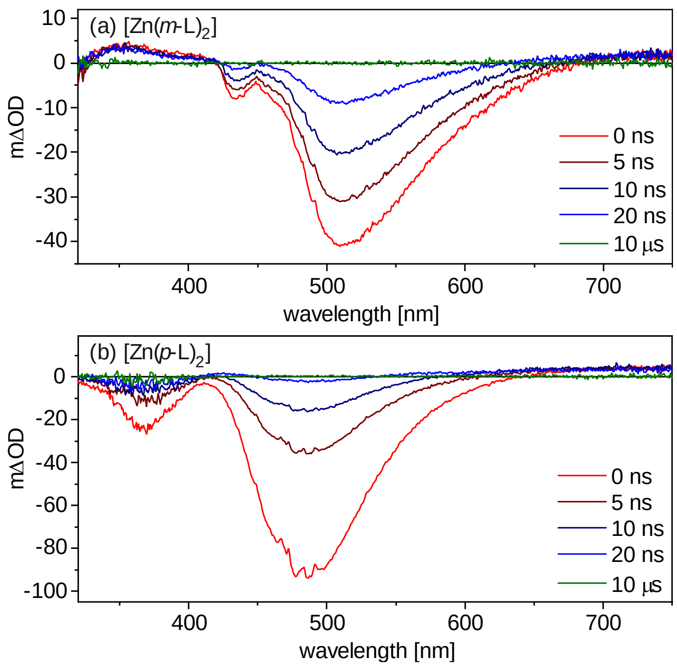

Figure S6: Transient absorption spectra of (a) [Zn(m-L)$_2$] and (b) [Zn(p-L)$_2$] in aerated toluene at 293 K, recorded at different time delays as indicated by the legends. [Zn(m-L)$_2$] was excited at 424 nm with laser pulse energies of 15 mJ, whereas [Zn(p-L)$_2$] was excited at 355 nm using laser pulses with 20 mJ energy. All spectra were recorded with an integration time of 200 ns after the indicated delay times.

Luminescence and intersystem crossing quantum yields and lifetimes

The measurements of the luminescence quantum yields were carried out using a relative method, where the integrals of the steady-state emission spectra of a reference compound and the sample were determined. Then the luminescence quantum yields were calculated using equation S1.

$$\phi_{fluo} = \phi_{fluo,r} \cdot \frac{I \cdot OD_r \cdot n^2}{I_r \cdot OD \cdot n_r^2} \qquad \text{eq. S1}$$

In equations S1, Φ_{fluo} is the luminescence quantum yield, I the integral of the emission, OD the optical density at the excitation wavelength, n the refractive index (from literature[5]) of the solvent. Parameters without any subscript stand for the data of the sample, whereas parameters containing the subscript "r" stand for the data obtained for a reference compound. Both the reference compound as well as the main sample were excited at the same wavelength, where the concentrations of two solutions were adjusted to the same optical density. The optical density at the excitation wavelength was kept between 0.1 and 0.05.

As reference compounds, $[Ru(bpy)_3](PF_6)_2$[6] (bpy = 2,2'-bipyridine) and fluorescein[7] were used.

Table S1: Luminescence quantum yields $\Phi_{fluo,r}$ at 293 K.

Reference	Solvent	$\Phi_{fluo,r}$ [%]
$[Ru(bpy)_3][PF_6]_2$	Aerated MeCN	1.8 [6]
Fluorescein	Deaerated 0.1 M aq. NaOH	95 [7]

Refractive indices n were used as displayed in Table S2[5]:

Table S2: Refractive indices (n) of used solvents for luminescence quantum yield calculations.

Solvent	n
H_2O	1.33
MeCN	1.34
CH_2Cl_2	1.42
Toluene	1.50

The intersystem crossing quantum yields (Φ_{isc}) were determined in a relative actinometry method using $[Ru(bpy)_3]Cl_2$ in H_2O as a reference system (Φ_{isc}=100%[8,9]) and solutions of [Zn(*m*-L)$_2$] or [Zn(*p*-L)$_2$ containing 10 mM of anthracene. The differences in refractive indices between the solvents were neglected in this case. The solution of one of the complexes (containing 10 mM anthracene in addition to the photosensitizer) and the corresponding reference solution of $[Ru(bpy)_3]Cl_2$ in H_2O (without added solutes) were adjusted to the same optical density of about 0.15 at the excitation wavelength (424 and 415 nm, respectively, see insets of Figure 6). Using

these solutions and identical settings at the transient absorption set-up can be expected to form identical amounts of photosensitizers in their initial singlet excited state. From time-resolved absorption measurements, we determined the initial ΔOD values at t = 0 (i. e., immediately after the excitation pulses), in order to calculate the concentrations of reference or acceptor molecules in the respective triplet excited states for the individual solutions:

$$c_{acc} = \frac{\Delta OD_{acc}}{\varepsilon_{acc} \cdot d} \qquad \text{eq. S2}$$

$$c_r = \frac{\Delta OD_r}{\Delta\varepsilon_r \cdot d} \qquad \text{eq. S3}$$

Where $\Delta\varepsilon_r$ is the known change in molar extinction coefficient of the ground-state MLCT bleach at 455 nm of excited $[Ru(bpy)_3]Cl_2$ ($\Delta\varepsilon_r$ = -10100 $M^{-1}cm^{-1}$) [10] and ε_{acc} is the molar extinction coefficient at the absorption maximum of triplet anthracene at 423 nm (53000 $M^{-1}cm^{-1}$).[11] d is the path length of the optical cuvette. As the intersystem crossing for $[Ru(bpy)_3]^{2+}$ is quantitative,[8,9] Φ_{isc} of the complex can be estimated as follows:

$$\phi_{isc} = \frac{c_{acc}}{c_r} \qquad \text{eq. S4}$$

Table S3: Luminescence and intersystem crossing quantum yields (Φ_{fluo} and Φ_{isc}), lifetimes and the corresponding radiative excited-state decay rate constants k_r, non-radiative rate constants k_{nr} and non-radiative rate constants originating from the triplet state $k_{nr, triplet}$ as illustrated in Figure 4 in deaerated toluene and deaerated CH_2Cl_2 at 293 K.[a]

toluene									
	$\tau_{singlet}$ [ns]	$\tau_{triplet}$ [μs]	Φ_{fluo} [%]	Φ_{isc} [%]	k_{tot} [s^{-1}]	k_r [s^{-1}]	k_{nr} [10^7 s^{-1}]	k_{isc} [s^{-1}]	$k_{nr, triplet}$ [s^{-1}]
m- LH	24	-	22	-	$4.2 \cdot 10^7$	$9.2 \cdot 10^6$	3.3	-	-
[Zn(*m*-L)$_2$]	25[a]	38	50	13	$4.0 \cdot 10^7$	$2.0 \cdot 10^7$	1.5	$5.2 \cdot 10^6$	$2.6 \cdot 10^4$
p-LH	5.5	-	94	-	$1.8 \cdot 10^8$	$1.7 \cdot 10^8$	1.0	-	-
[Zn(*p*-L)$_2$]	5.5	62	86	7.2	$1.8 \cdot 10^8$	$1.5 \cdot 10^8$	1.2	$1.3 \cdot 10^7$	$1.6 \cdot 10^4$
CH_2Cl_2									
	$\tau_{singlet}$ [ns]	$\tau_{triplet}$ [μs]	Φ_{fluo} [%]	Φ_{isc} [%]	k_{tot} [s^{-1}]	k_r [s^{-1}]	k_{nr} [10^7 s^{-1}]	k_{isc} [s^{-1}]	$k_{nr, triplet}$ [s^{-1}]
m-LH	9.1	-	10	-	-	$1.1 \cdot 10^7$	9.9	-	-
[Zn(*m*-L)$_2$]	9.0	-	7.6	-	-	$8.4 \cdot 10^6$	10	-	-
p-LH	5.6	-	47	-	-	$8.5 \cdot 10^7$	9.5	-	-
[Zn(*p*-L)$_2$]	5.5	-	49	-	-	$8.8 \cdot 10^7$	9.2	-	-

[a] Lifetimes of biexponential decays were averaged in a weighted manner (see Figure S7).

The rate constants k_{tot}, k_r, k_{nr}, k_{isc}, and $k_{nr, triplet}$ were calculated according to equations S5-S9.

$$k_{tot} = k_r + k_{nr} + k_{isc} = \frac{1}{\tau_{singlet}} \quad \text{eq. S5}$$

$$k_r = \phi_{fluo} \cdot k_{tot} \quad \text{eq. S6}$$

$$k_{nr} = (1 - \phi_{isc} - \phi_{fluo}) \cdot k_{tot} \quad \text{eq. S7}$$

$$k_{isc} = \phi_{isc} \cdot k_{tot} \quad \text{eq. S8}$$

$$k_{nr,triplet} = \frac{1}{\tau_{triplet}} \quad \text{eq. S9}$$

Kinetics

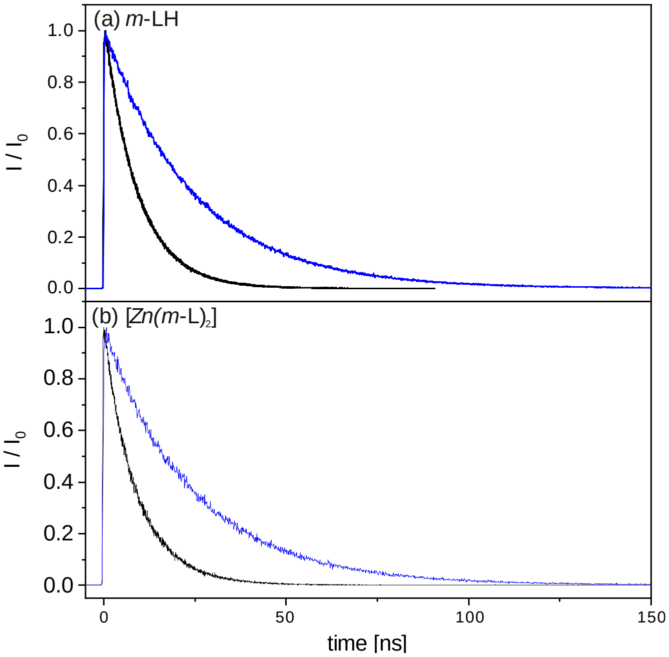

Figure S7: Emission decay curves of (a) *m*-LH detected at 500 nm in deaerated toluene (blue) and at 550 nm in deaerated CH_2Cl_2 (black), and (b) [Zn(*m*-L)$_2$] detected at 500 nm in deaerated toluene (blue) and at 550 nm in deaerated CH_2Cl_2 (black) at 298 K measured in a TCSPC experiment. All samples were excited at 405 nm.

The luminescence decay data of [Zn(*m*-L)$_2$] was fitted using a biexponential decay function according to eq. S10.

$$y(t) = A_1 \exp\left(-\frac{t}{\tau_1}\right) + A_2 \exp\left(-\frac{t}{\tau_2}\right) \qquad \text{eq. S10}$$

The luminescence decays of [Zn(*m*-L)$_2$] were composed of a decay component with τ_1 = 21 ns (τ_1, A_1=50%) and a second decay component with τ_2 = 28 ns (A_2=50%) in toluene and τ_1 = 7.2 ns (τ_1, A_1=52%) and τ_2 = 11 ns (τ_2, A_2=48%) in CH_2Cl_2.

The average lifetimes for [Zn(*m*-L)$_2$] in Table 1 and Table S3 are weighted averages calculated according to eq. S11.

$$\tau_{singlet} = \frac{A_1}{A_1 + A_2}\tau_1 + \frac{A_2}{A_1 + A_2}\tau_2 \qquad \text{eq. S11}$$

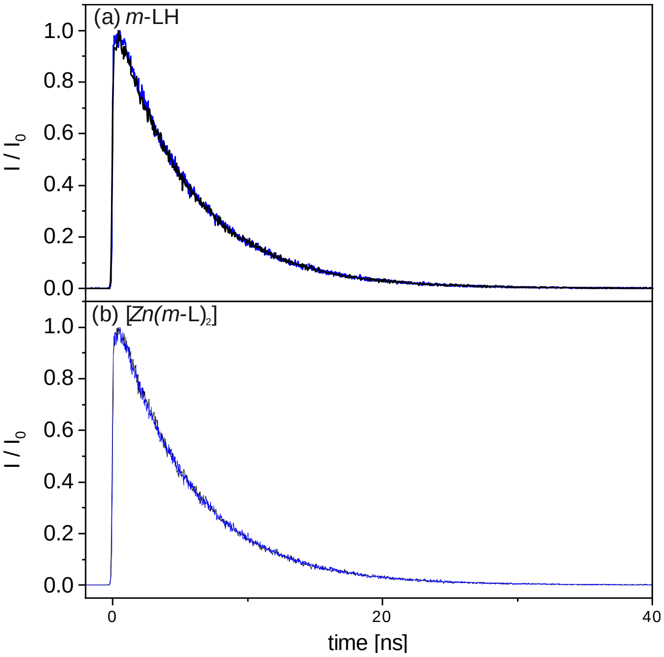

Figure S8: Emission decay curves of (a) *p*-LH detected at 500 nm in deaerated toluene (blue) and at 550 nm in deaerated CH_2Cl_2 (black), and (b) [Zn(*p*-L)$_2$] detected at 500 nm in deaerated toluene (blue) and 550 nm in deaerated CH_2Cl_2 (black) at 298 K measured in a TCSPC experiment. All samples were excited at 405 nm.

The emission decays of [Zn(*p*-L)$_2$] were single exponential in all recorded cases.

UV-Vis transient absorption spectra of the [Zn(*m*-L)$_2$] and the [Zn(*p*-L)$_2$] complex are included in the main paper (Figure 5). The photoactive ^{3}ILCT state of [Zn(*m*-L)$_2$] complex exhibits a prominent excited-state absorption (ESA) band at 350 nm, which was monitored to determine the ^{3}ILCT lifetime of this compound (Figure S9). In the case of [Zn(*p*-L)$_2$], it was more convenient to monitor a ground-state absorption bleach (GSB) at 390 nm (Figure S10).

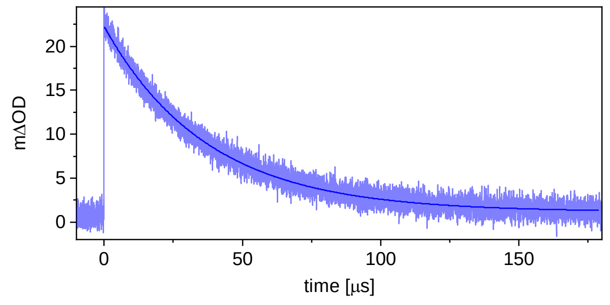

Figure S9: Decay of the transient absorption signal of [Zn(*m*-L)$_2$] at 350 nm ($\tau_{triplet}$=38 μs) in deaerated toluene at 293 K. Excitation occurred at 424 nm with laser pulses of ca. 10 ns duration and a pulse energy of 11 mJ.

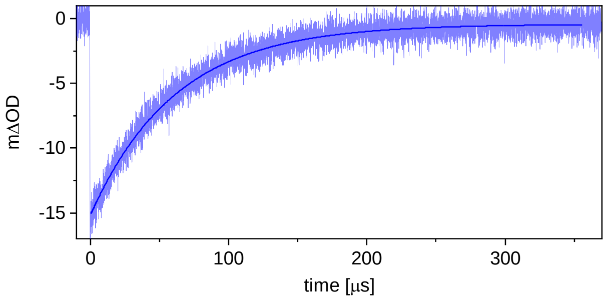

Figure S10: Recovery of the bleach at 390 nm in the transient absorption spectrum of [Zn(*p*-L)$_2$] ($\tau_{triplet}$=62 μs) in deaerated toluene at 293 K. Excitation occurred at 355 nm with laser pulses of ca. 10 ns duration and a pulse energy of 35 mJ.

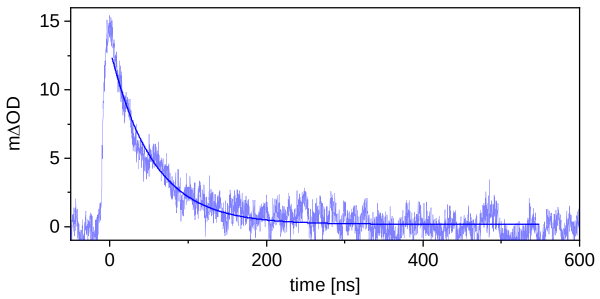

Figure S11: Decay of the transient absorption signal of [Zn(m-L)$_2$] at 350 nm ($\tau_{triplet}$=53.9 ns) in aerated toluene at 293 K. Excitation occurred at 424 nm with laser pulses of ca. 10 ns duration and a pulse energy of 15 mJ.

Cyclic voltammetry

For solubility reasons, cyclic voltammetry of the free (protonated) ligands was performed in acetonitrile, whereas dichloromethane was used for the two Zn^{II} complexes. The *m*-LH ligand shows on oxidation wave at 0.61 V vs SCE (Figure S12), which we attribute to a triarylamine-based oxidation process. The respective potential value seems in line with typical one-electron oxidation potentials of comparable triarylamines.[12] An irreversible reduction wave is furthermore observed below -2.0 V vs SCE, but this is not of interest for the current study.

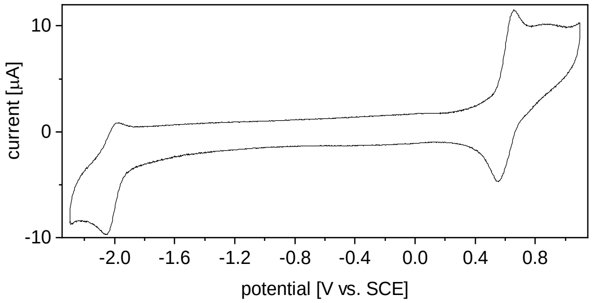

Figure S12: Cyclic voltammogram of *m*-LH in deaerated MeCN. 0.1 M $n\mathrm{Bu_4PF_6}$ was used as an electrolyte and the potential scan rate was 0.1 Vs^{-1}.

In the cyclic voltammogram of $[\mathrm{Zn}(m\text{-L})_2]$, the analogous triarylamine-based oxidation process occurs at similar potential as in the free *m*-LH ligand (Figure S13), but it seems that the consecutive one-electron oxidation of both coordinated ligands occurs at slightly different potentials (0.32 and 0.45 V vs SCE). Thus, it seems that oxidation of one *m*-LH ligand makes the oxidation of the second coordinated *m*-LH ligand somewhat more difficult, presumably for electrostatic reasons. Further oxidation of the two triarylamines (to their dication forms) then occurs as a single wave at 0.89 V vs SCE.

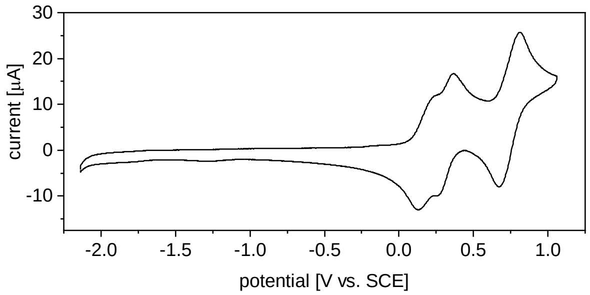

Figure S13: Cyclic voltammogram of $[\mathrm{Zn}(m\text{-L})_2]$ in deaerated CH_2Cl_2. 0.1 M $\mathrm{N}(n\mathrm{Bu})_4\mathrm{PF_6}$ was used as an electrolyte and the scan rate was 0.1 Vs^{-1}. The voltammogram was measured in a glovebox and referenced to $\mathrm{Fe(Cp^*)_2}$.[13]

In the cyclic voltammogram of the free *p*-LH ligand (Figure S14), the primary triarylamine oxidation occurs at 0.80 V vs SCE, i. e., at substantially higher potential than in the free *m*-LH ligand. Similarly, in the [Zn(*p*-L)$_2$] complex, the analogous oxidation process occurs at 0.80 V vs SCE (Figure S12). We tentatively attribute the higher triarylamine oxidation potential in the [Zn(*p*-L)$_2$] and *p*-LH compounds to different extents of electronic coupling between the phenolic substituent (the oxygen ligand atom) and the triarylamine N-atom. In [Zn(*p*-L)$_2$] and *p*-LH, the phenolic substituent stands in *meta*-position to the triarylamine N-atom, whereas in the [Zn(*m*-L)$_2$] and *m*-LH compounds there is a *para*-substitution pattern.

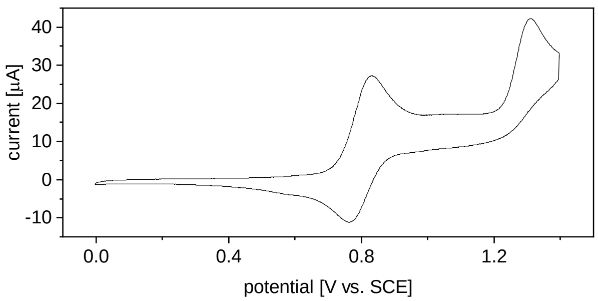

Figure S14: Oxidative cyclic voltammogram of *p*-LH in deaerated MeCN. 0.1 M N(*n*Bu)$_4$PF$_6$ was used as an electrolyte and the scan rate was 0.1 Vs^{-1}.

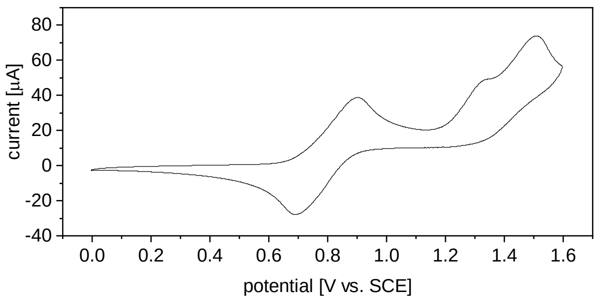

Figure S15: Oxidative cyclic voltammogram of [Zn(*p*-L)$_2$] in deaerated CH_2Cl_2. 0.1 M N(*n*Bu)$_4$PF$_6$ was used as an electrolyte and the scan rate was 0.1 Vs^{-1}.

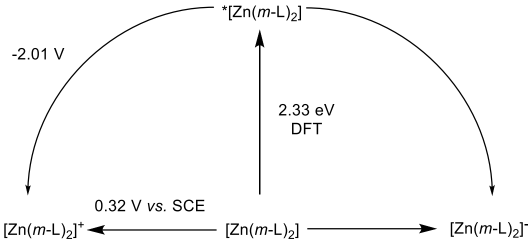

Figure S16: Latimer diagram of [Zn(*m*-L)] with the estimated excited state oxidation potential calculated based on the excited state energy from TD-DFT and the ground state oxidation potential determined by cyclic voltammetry (see Figure S13).

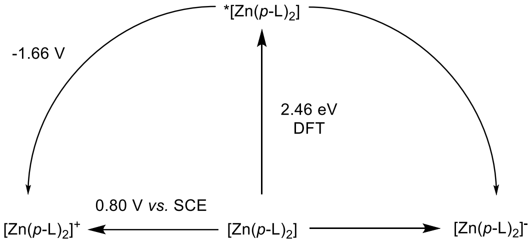

Figure S17: Latimer diagram of [Zn(*p*-L)$_2$] with the calculated excited state oxidation potential calculated from the excited state energy from TD-DFT and the ground state oxidation potential measured in cyclic voltammetry (see Figure S15).

PHOTOCATALYSIS

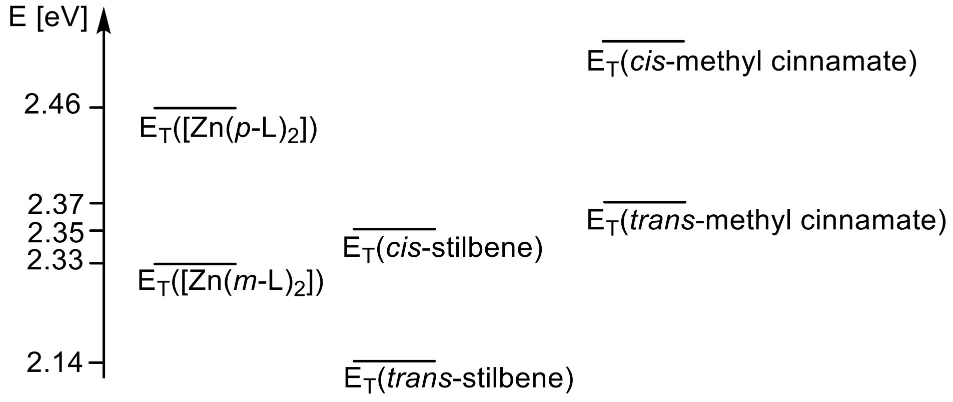

Figure S18: Energies of the lowest triplet excited states (E_T) of the two complexes [Zn(*m*-L)$_2$] and [Zn(*p*-L)$_2$], and of the *cis*- and *trans*-isomers of the substrates stilbene and methyl cinnamate.

Photoisomerization of trans-stilbene

To estimate the yield of the *cis*-stilbene product in the reaction shown in Table 2, ^{1}H-NMR resonances attributable to the *trans*-stilbene starting material and the *cis*-stilbene product were compared. Specifically, the integrals of the multiplet in the ^{1}H-NMR spectrum at 7.37-7.30 (I_{trans}) due to *trans*-stilbene and the singlet at 6.47 (I_{cis}, marked by the triangles in Figure S19 and Figure S20) attributable to *cis*-stilbene were used.

The yields (y_{cis} and y_{trans}) were calculated using eq. S12 and eq. S13.

$$y_{cis} = \frac{I_{cis}}{I_{cis} + I_{trans}} \quad \text{eq. S12}$$

$$y_{trans} = \frac{I_{trans}}{I_{cis} + I_{trans}} \quad \text{eq. S13}$$

For the calculation of the ratio of cis-stilbene to trans-stilbene in Figure S22, y_{cis} was divided by y_{trans}.

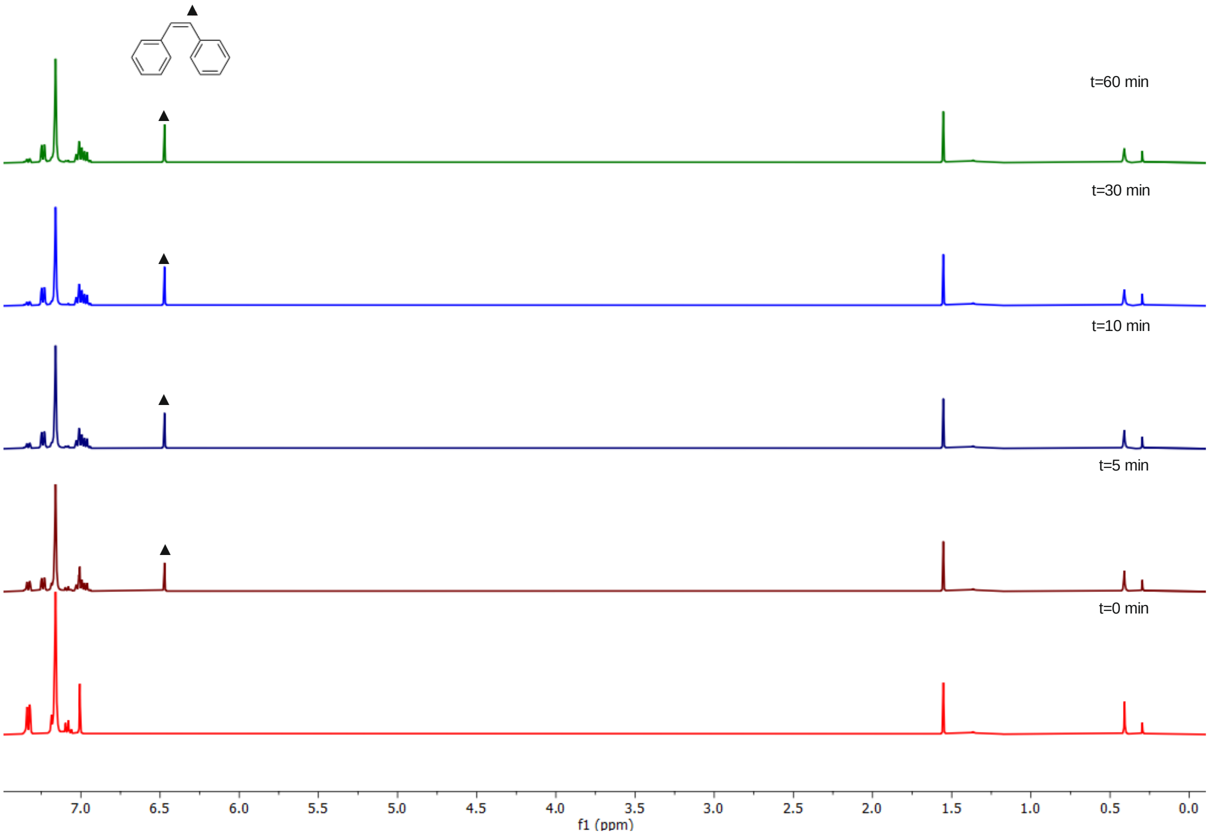

Figure S19: Stacked ^{1}H-NMR spectra in C_6D_6 at 298 K monitoring the photoisomerization of *trans*-stilbene with the [Zn(*m*-L)$_2$] sensitizer as a function of irradiation time as indicated on the right-hand side.

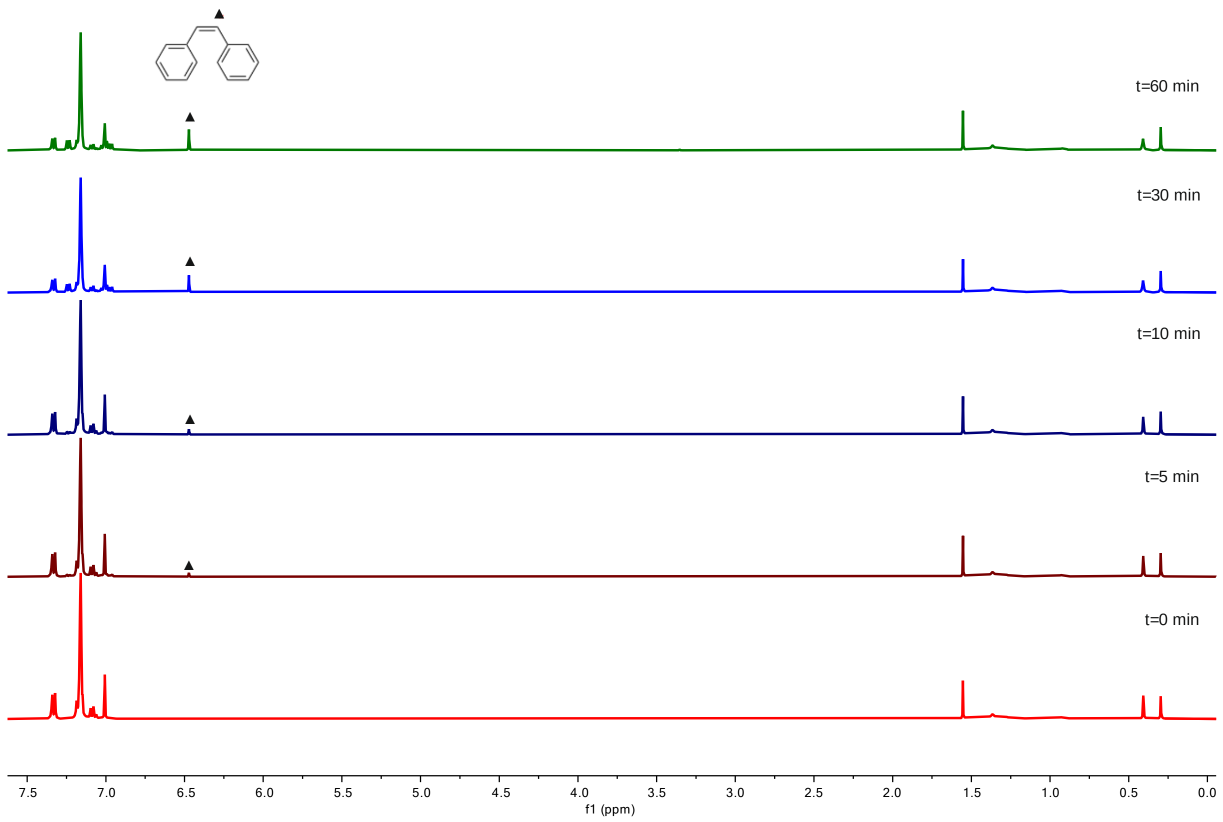

Figure S20: Stacked ^{1}H-NMR spectra in C_6D_6 at 298 K monitoring the photoisomerization of *trans*-stilbene with the $[Zn(p\text{-}L)_2]$ sensitizer as a function of irradiation time as indicated on the right-hand side.

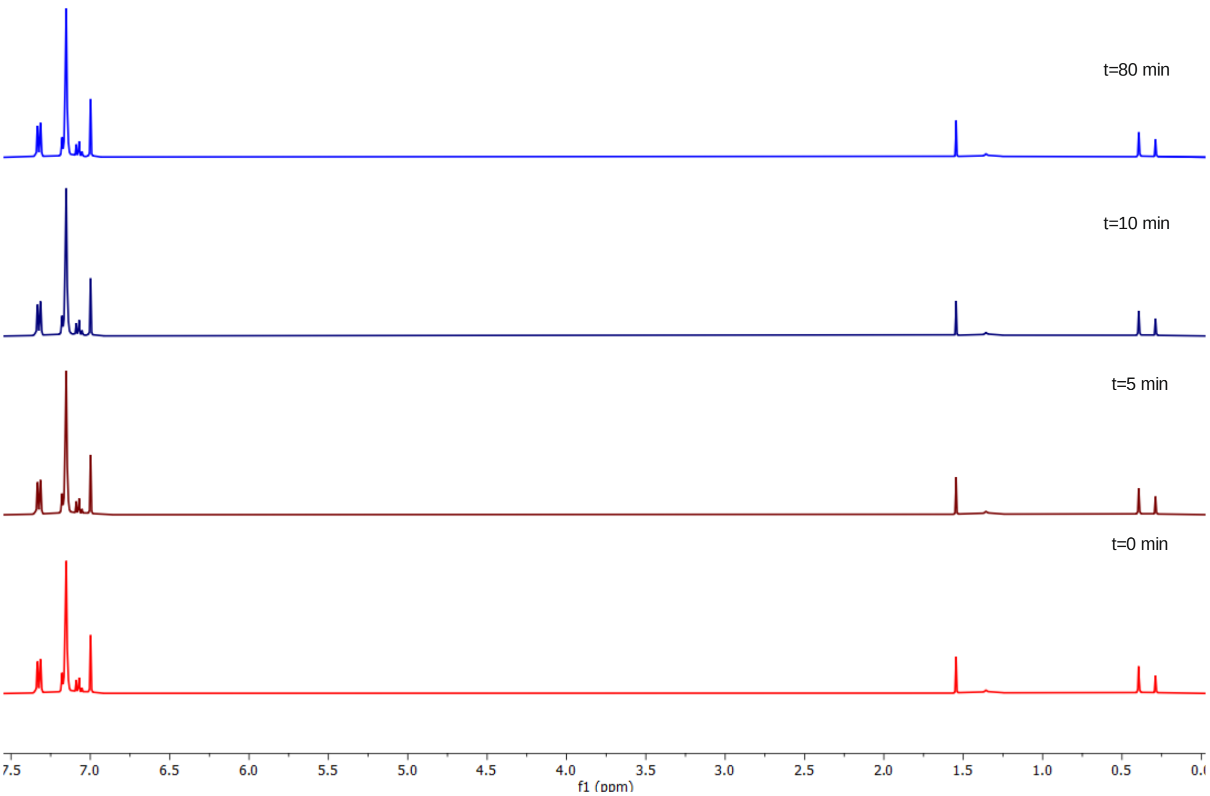

Figure S21: Stacked ^{1}H-NMR spectra in C_6D_6 at 298 K monitoring possible changes in the reaction mixture composition in the absence of the photosensitizer as a function of irradiation time as indicated on the right-hand side. This control experiment shows that the Zn^{II} photosensitizer is an essential ingredient for the photo-isomerization of *trans*-stilbene under these conditions.

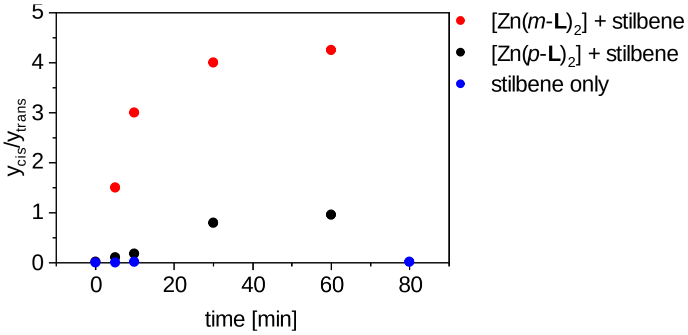

Figure S22: Ratios of the yields of *cis*-stilbene and *trans*-stilbene monitored over time using the integrals of the ^{1}H-NMR resonances as discussed above.

PHOTOISOMERIZATION OF METHYL *TRANS*-CINNAMATE

In ^{1}H-NMR spectroscopy, the integral of the signal of the internal standard Me_3SiPh was normalized to 1. The integrals of the methyl group resonance of the methyl *trans*-cinnamate substrate (I_{trans}) at 3.27 ppm and the methyl *cis*-cinnamate product (I_{cis}) at 3.11 ppm were used for Figure S26*:* and Table 2.

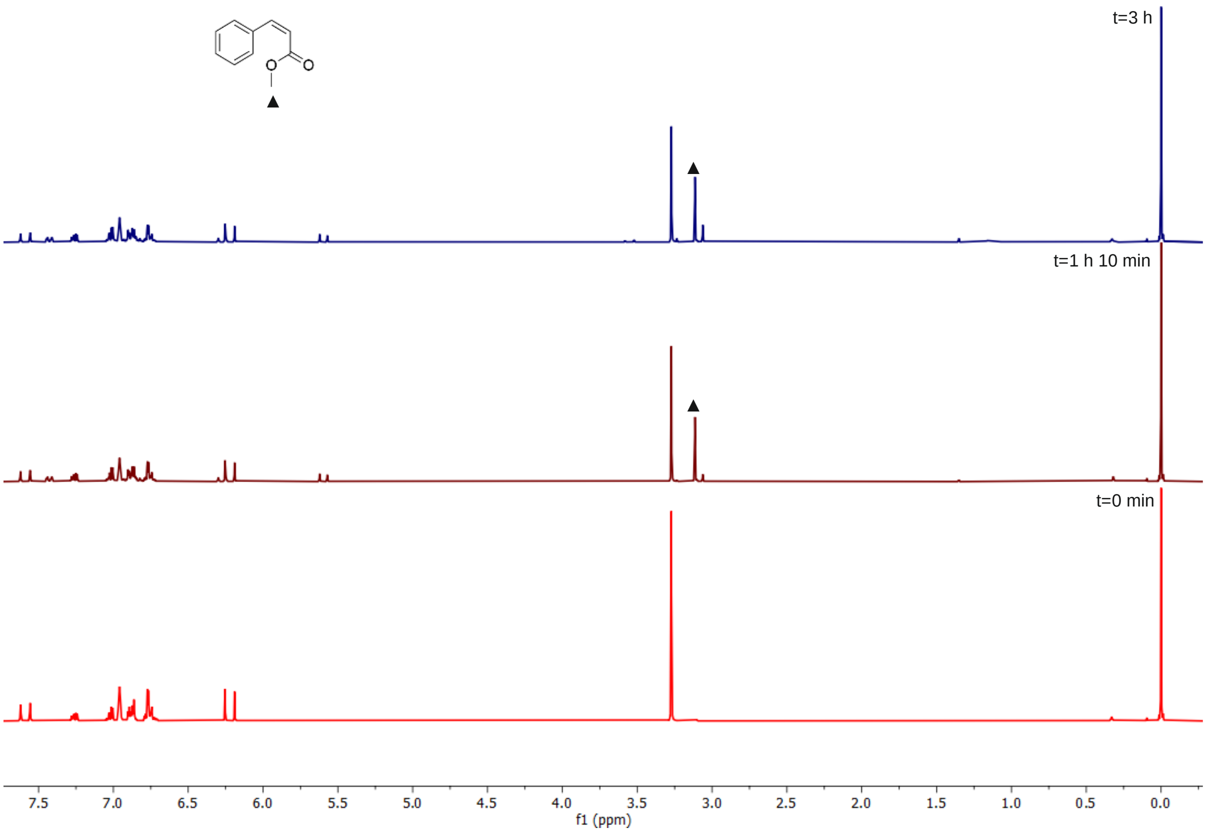

Figure S23: Stacked ^{1}H-NMR spectra in C_6D_6 at 298 K monitoring the photoisomerization of methyl *trans*-cinnamate with the [Zn(*m*-L)$_2$] sensitizer as a function of irradiation time as indicated on the right-hand side.

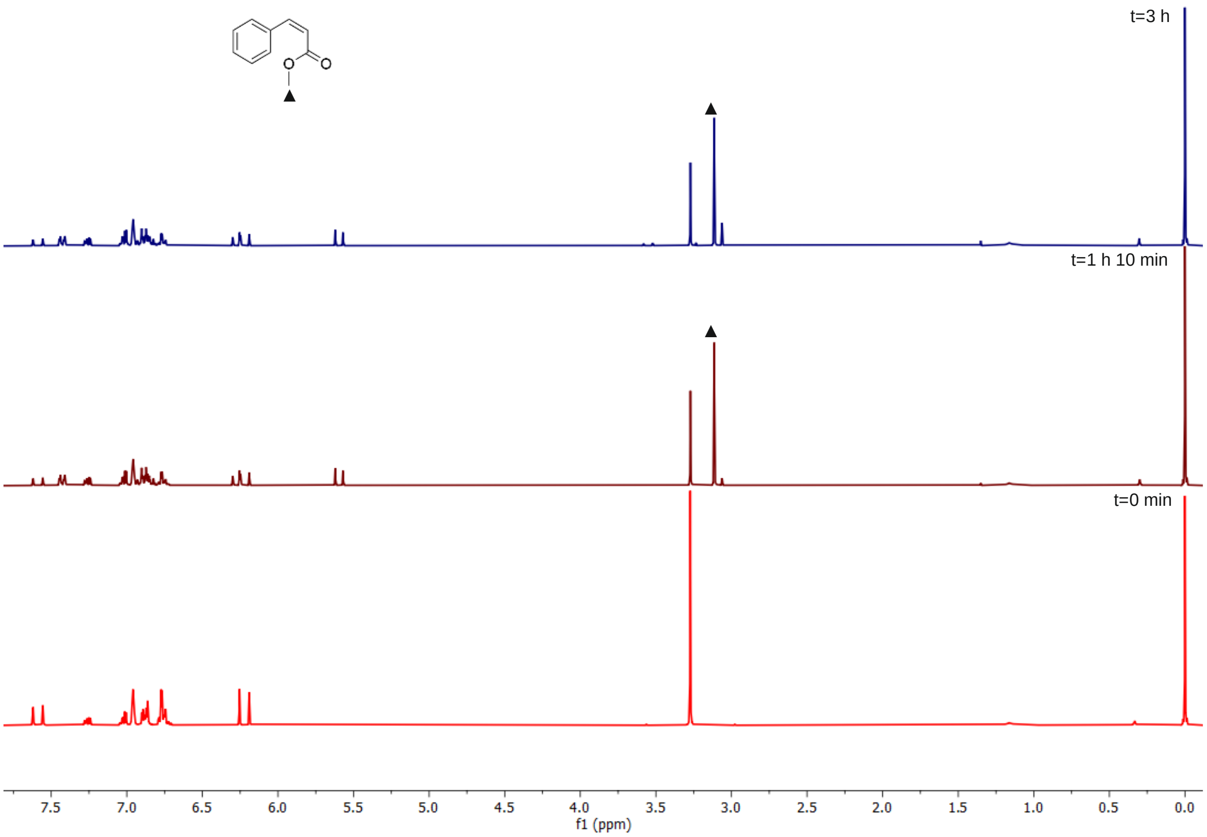

Figure S24: Stacked ^{1}H-NMR spectra in C_6D_6 at 298 K monitoring the photoisomerization of methyl *trans*-cinnamate with the [Zn(*p*-L)$_2$] sensitizer as a function of irradiation time as indicated on the right-hand side.

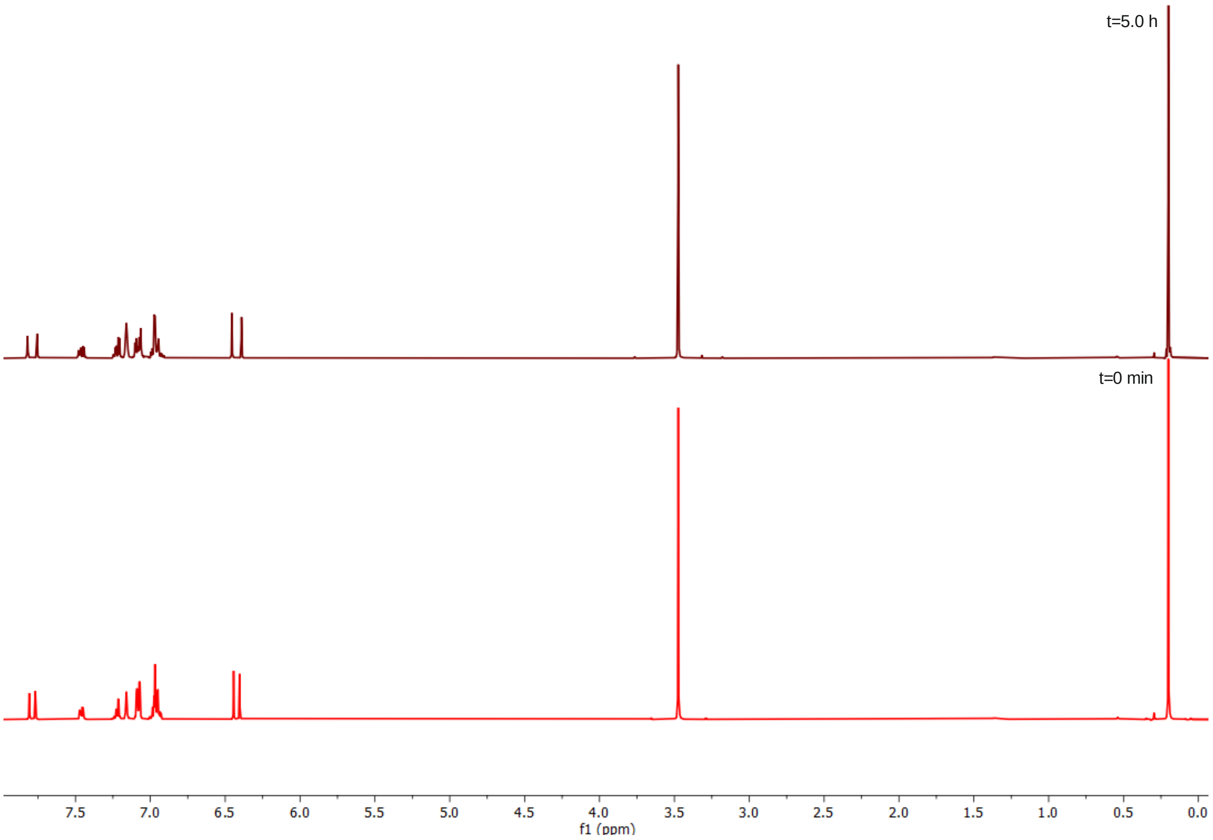

Figure S25: :Stacked ^{1}H-NMR spectra in C_6D_6 at 298 K monitoring possible changes in the reaction mixture composition in the absence of the photosensitizer as a function of irradiation time as indicated on the right-hand side. This control experiment shows that the Zn(II) photosensitizer is an essential ingredient for the photo-isomerization of methyl *trans*-cinnamate under these conditions.

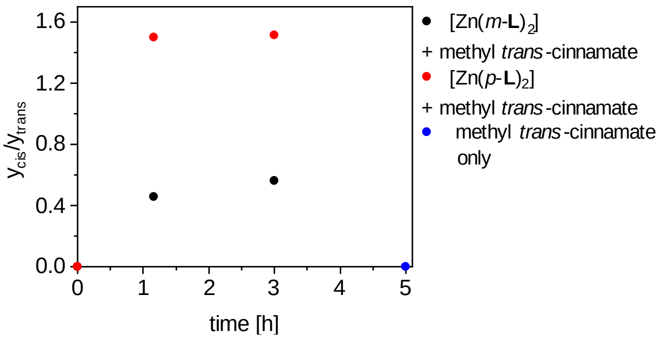

Figure S26: Ratios of the yields of methyl *cis*-cinnamate over methyl *trans*-cinnamate monitored over time using the integrals of the ^{1}H-NMR resonances of the corresponding cinnamate.

Photocatalytic decarboxylation

In ^{1}H-NMR spectroscopy, the integral of the signal of the internal standard Me_3SiPh was normalized to 1. The integral of the *tert*-butyl-group resonance attributable to the reactant ($I_{reactant}$, singlet at 1.32 ppm) was used as a measure for the consumption of the reactant. The ^{1}H-NMR integral of the corresponding *tert*-butyl-group resonance in the product 1-(*tert*-butyl)benzene-4-d ($I_{product}$, singlet at 1.36 ppm) corresponds to the yield of the latter.

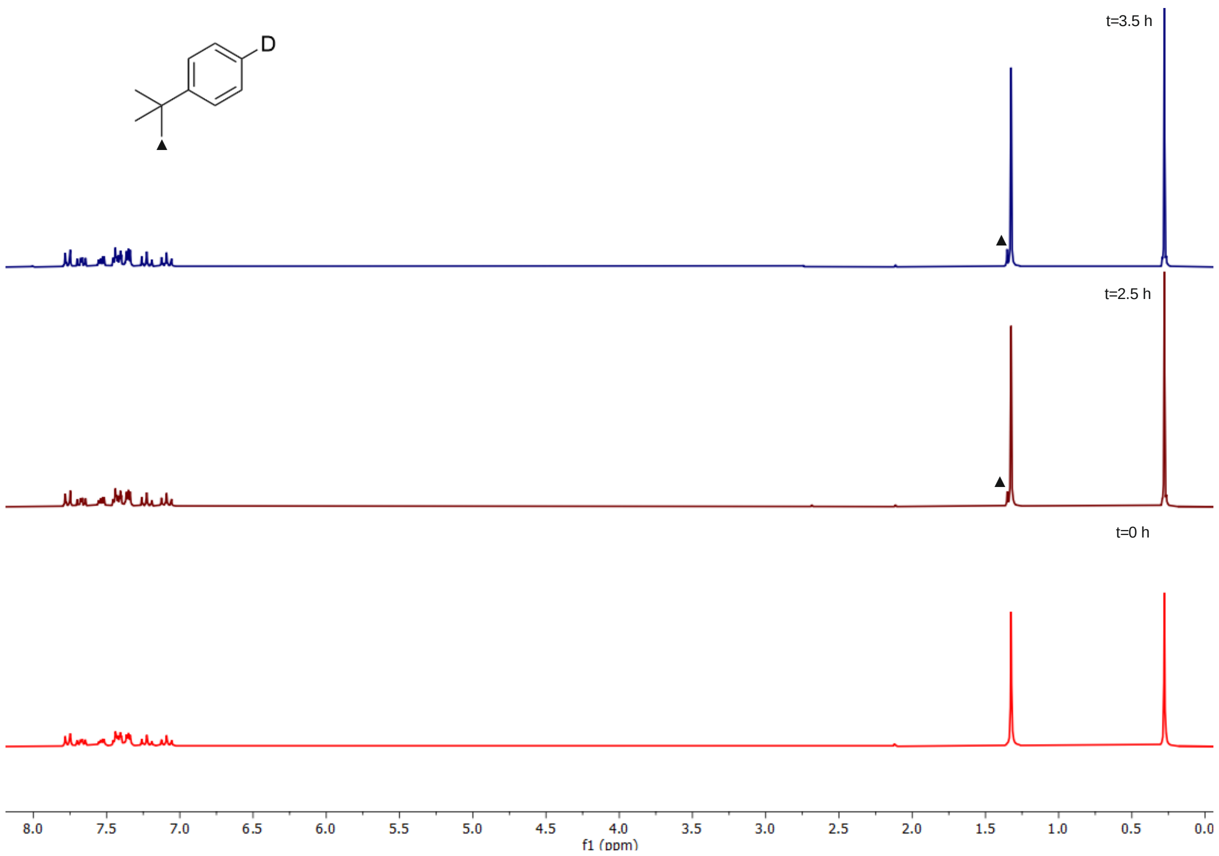

Figure S27: Stacked ^{1}H-NMR spectra in C_6D_6 at 298 K monitoring the photocatalytic decarboxylation of bis(4-fluorophenyl)methanone O-(4-(tert-butyl)benzoyl) oxime with the [Zn(*m*-L)$_2$] sensitizer as a function of irradiation time as indicated on the right-hand side.

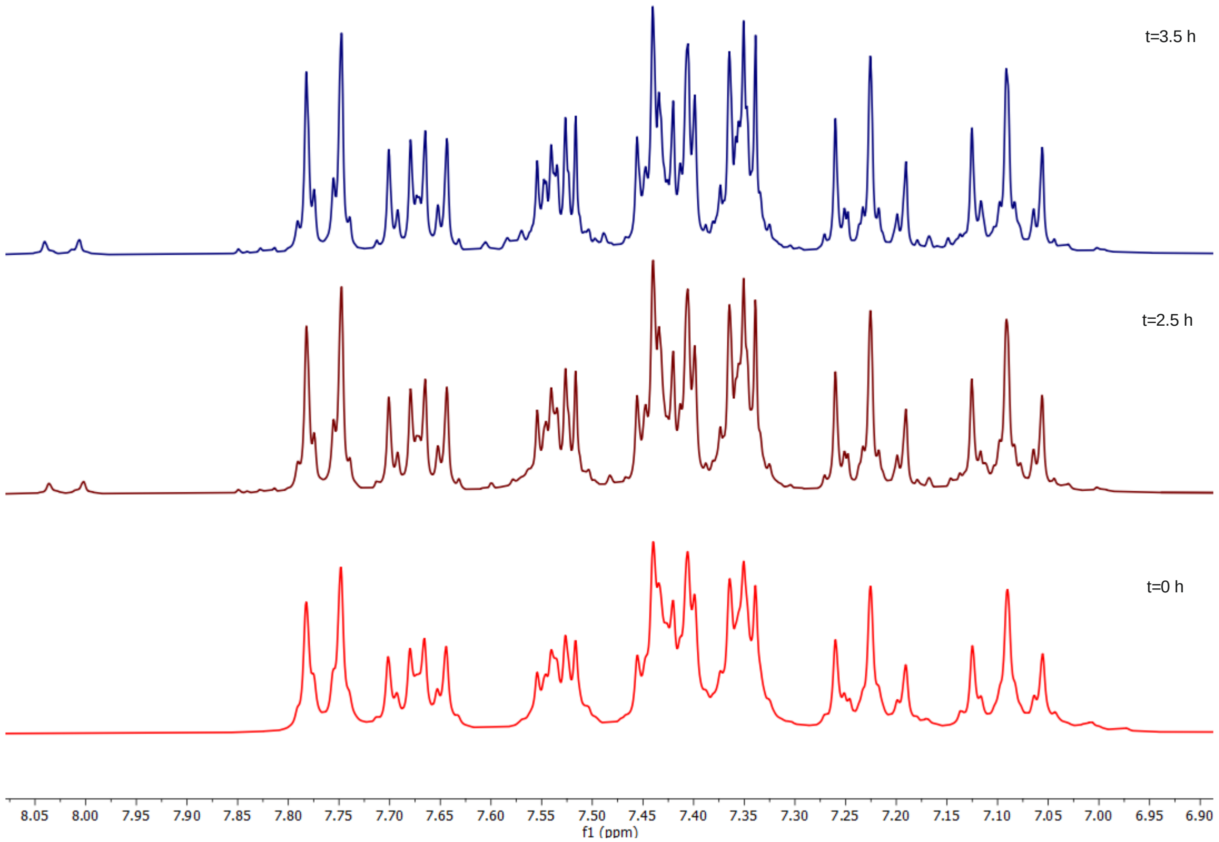

Figure S28: Zoom in the aromatic region of the stacked ^{1}H-NMR spectra in C_6D_6 at 298 K monitoring the photocatalytic decarboxylation of bis(4-fluorophenyl)methanone O-(4-(tert-butyl)benzoyl) oxime with the [Zn(*m*-L)$_2$] sensitizer as a function of irradiation time as indicated on the right-hand side.

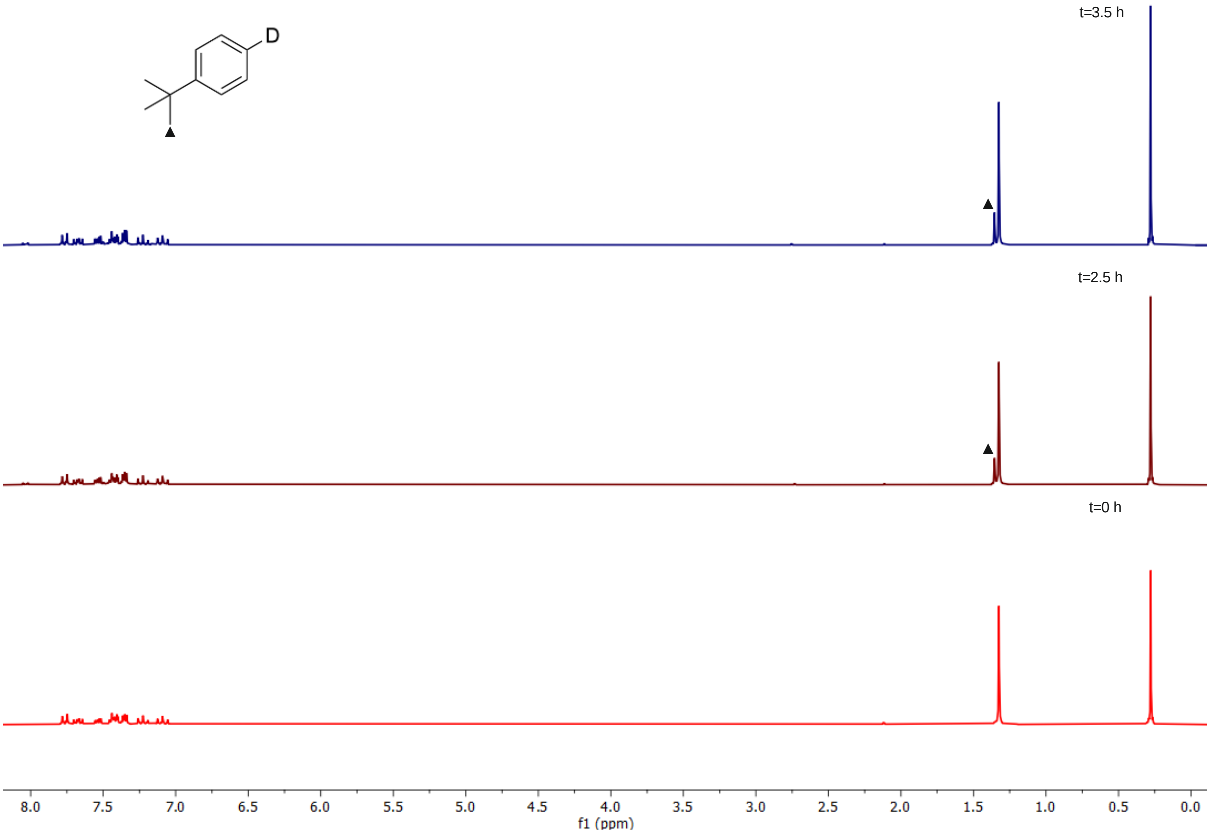

Figure S29: Stacked ^{1}H-NMR spectra in C_6D_6 at 298 K monitoring the photocatalytic decarboxylation of bis(4-fluorophenyl)methanone O-(4-(tert-butyl)benzoyl) oxime with the [Zn(*p*-L)$_2$] sensitizer as a function of irradiation time as indicated on the right-hand side.

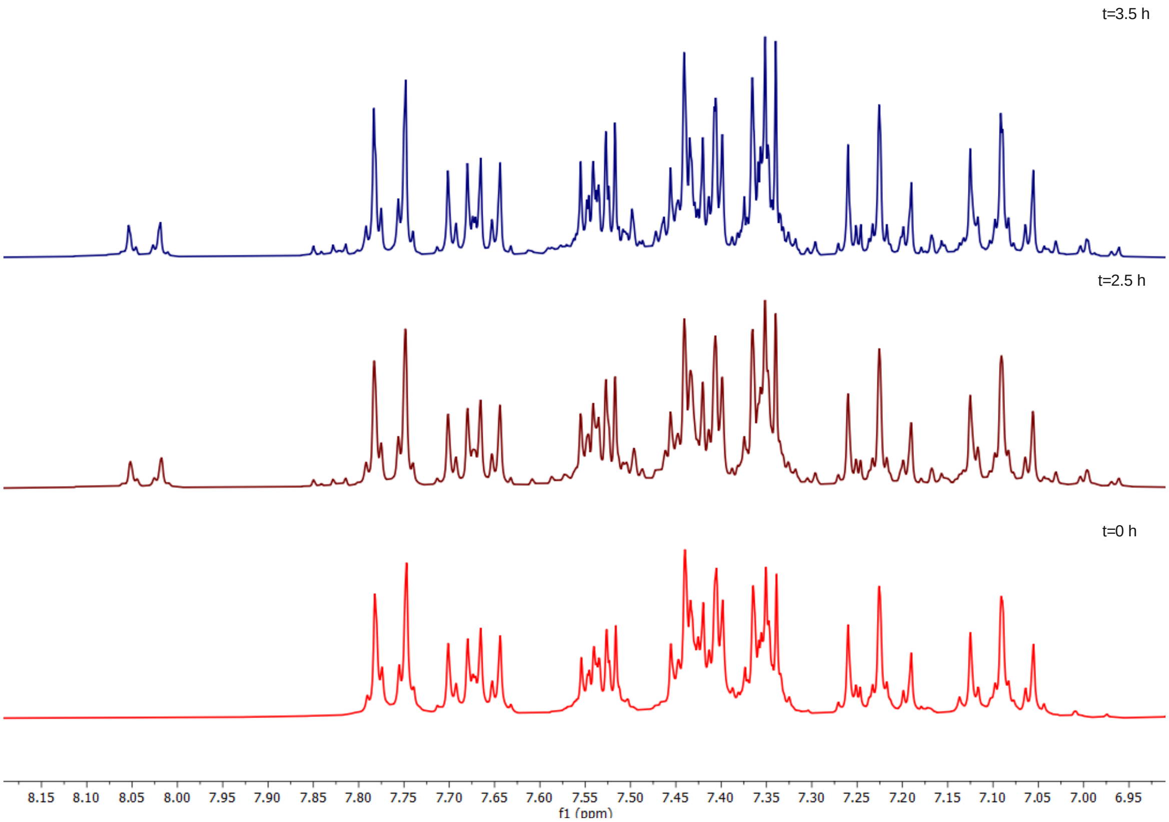

Figure S30: Zoom in the aromatic region of the stacked ^{1}H-NMR spectra in C_6D_6 at 298 K monitoring the photocatalytic decarboxylation of bis(4-fluorophenyl)methanone O-(4-(tert-butyl)benzoyl) oxime with the [Zn(*p*-L)$_2$] sensitizer as a function of irradiation time as indicated on the right-hand side.

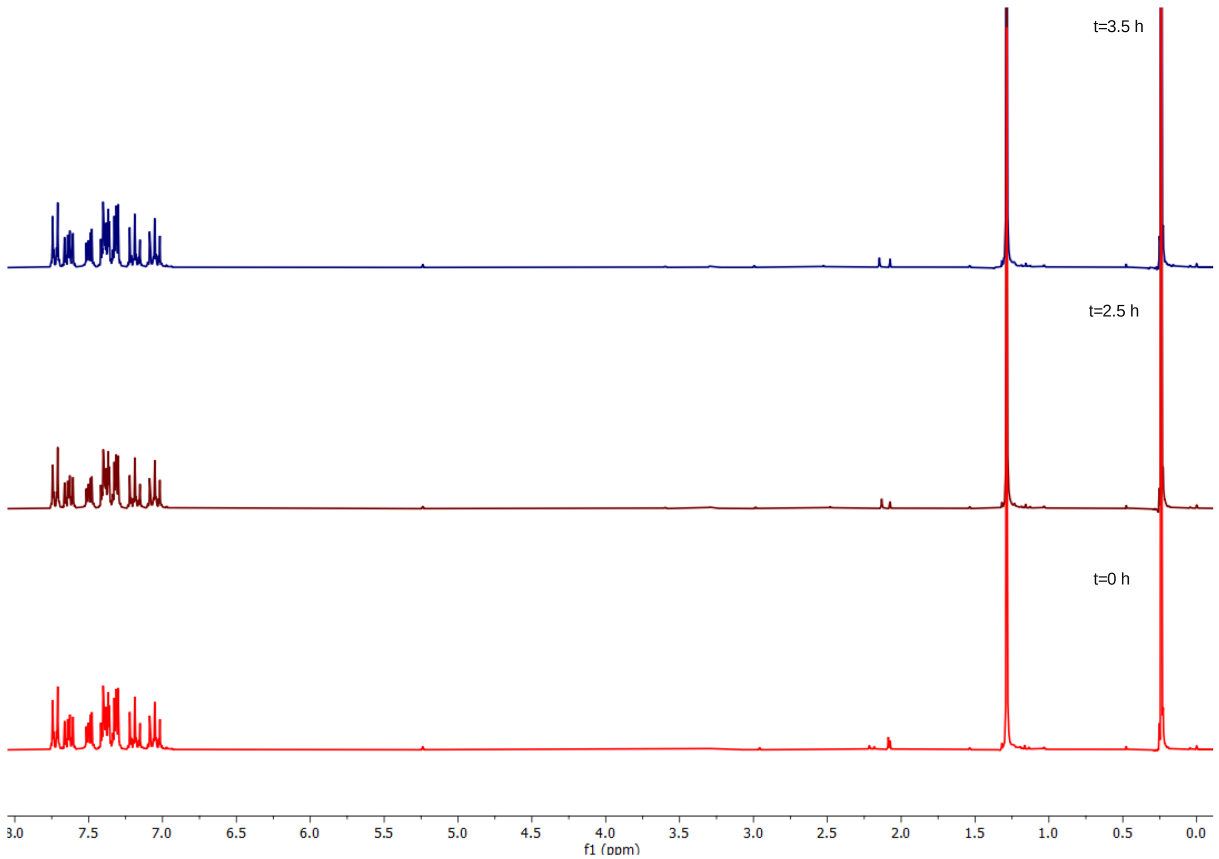

Figure S31: Stacked ^{1}H-NMR spectra in C_6D_6 at 298 K monitoring possible changes in the reaction mixture composition in the absence of the photosensitizer as a function of irradiation time as indicated on the right-hand side. This control experiment shows that the Zn(II) photosensitizer is an essential ingredient for the photocatalytic decarboxylation of bis(4-fluorophenyl)methanone O-(4-(tert-butyl)benzoyl) oxime.

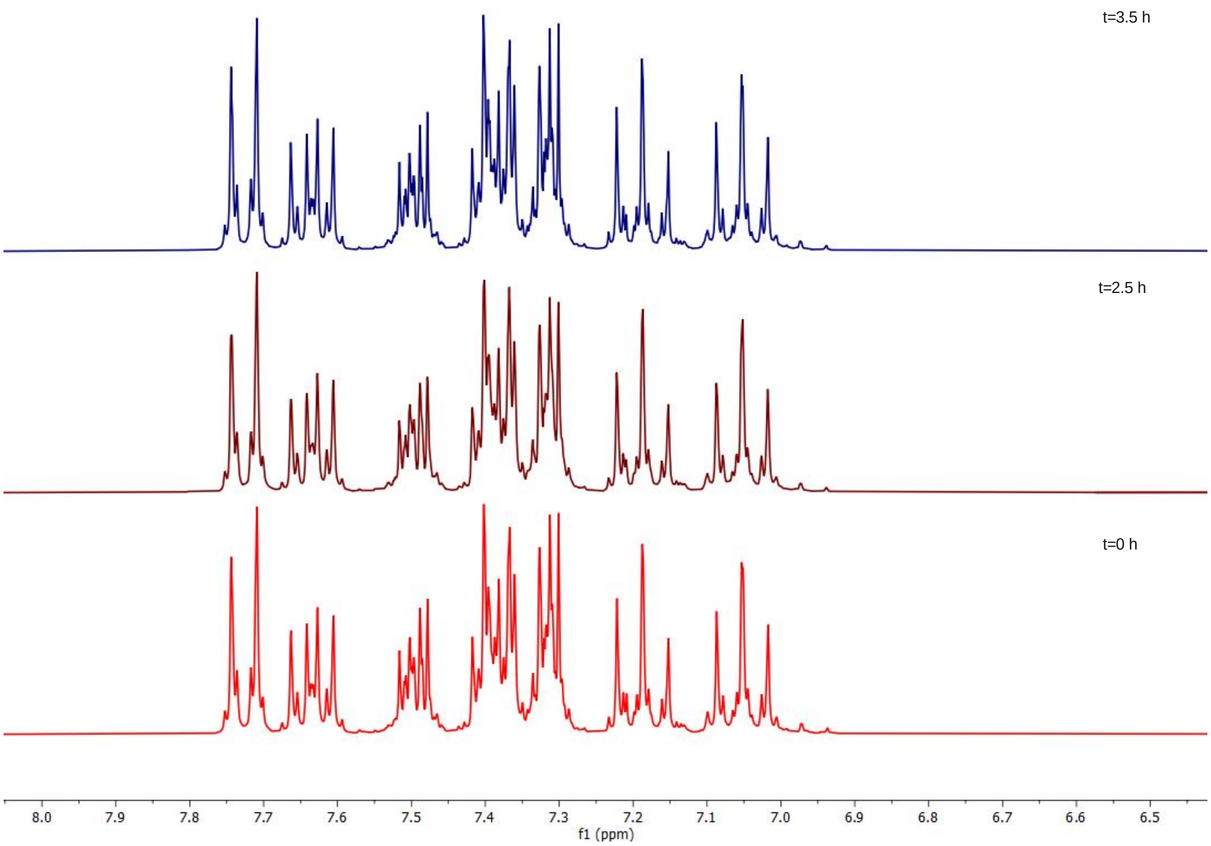

Figure S32: Zoom in the aromatic region of the stacked ^{1}H-NMR spectra in C_6D_6 at 298 K monitoring possible changes in the reaction mixture composition in the absence of the photosensitizer as a function of irradiation time as indicated on the right-hand side. This control experiment shows that the Zn(II) photosensitizer is an essential ingredient for the photocatalytic decarboxylation of bis(4-fluorophenyl)methanone O-(4-(tert-butyl)benzoyl) oxime.

PHOTOSTABILITY

The change in photosensitizer concentration Δc as a function of irradiation time was calculated according to eq. S14.

$$\Delta c = c_0 \cdot (I_0 - 1) \qquad \text{eq. S14}$$

In equation S14, I_0 is the normalized luminescence intensity of the intact photosensitizer prior to any photo-degradation and c_0 is the concentration of the photosensitizer at the beginning of the long-term irradiation experiment. c_0 was determined based on the photosensitizer's absorption spectrum and the molar extinction coefficient ε at 405 nm using the Lambert-Beer law (eq. S15).

$$c_0 = \frac{A_0}{\varepsilon \cdot d} \qquad \text{eq. S15}$$

In eq. S15, d is the path length of the cuvette. The initial photosensitizer concentrations were c_0 = 11.8 µM for [Zn(*m*-L)$_2$], c_0 = 3.00 µM for [Zn(*p*-L)$_2$], and c_0 = 1.26 µM for $[Ru(bpy)_3]^{2+}$. These concentrations were chosen such that A_0 is essentially identical for all three solutions (Table S4), to ensure that equal amounts of laser light are initially absorbed. To make the individual photostability data sets more directly comparable to one another, the representation used in Figure S33, with changes of concentrations (Δc) rather than absolute concentrations, seemed meaningful.[14]

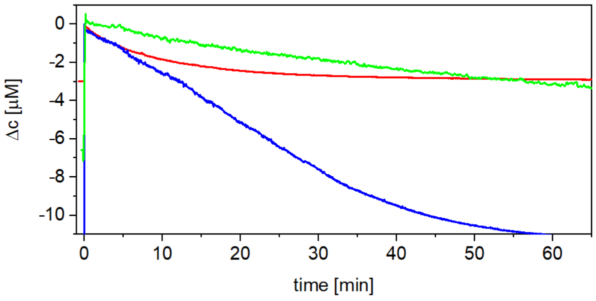

Figure S33: Photostability of [Zn(*m*-L)$_2$] (blue), [Zn(*p*-L)$_2$] (red), and $[Ru(bpy)_3]^{2+}$ (green) in deaerated solutions at 293 K. Luminescence intensity changes were monitored as a function of irradiation time and converted to concentration changes (Δc) as described above. The excitation source was a 405 nm continuous-wave laser with a power output of 0.526 W. The solvent was toluene for the zinc(II) complexes and water for $[Ru(bpy)_3]^{2+}$. Luminescence intensities were detected at 510 nm in the case of the zinc(II) complexes and at 550 nm in the case of the ruthenium(II) complex.

For our purposes here, we define the photodecomposition quantum ϕ_{decomp} yield as the number of decomposed photosensitizer molecules (n_{decomp}) divided by the number of absorbed photons (n_{abs})

as in eq. S16. We determined ϕ_{decomp} from the point at which I/I_0=0.9, i. e., at the point at the luminescence intensity (I) has decreased to 90% of its initial value (I_0).

$$\phi_{decomp} = \frac{n_{decomp}}{n_{abs}} \qquad \text{eq. S16}$$

The number of photons emitted by the (n_{laser}) was calculated according to eq. S17.

$$n_{laser} = \frac{power\ output\ of\ laser\ \cdot time}{energy\ of\ a\ photon\ at\ 405\ nm \cdot N_A} = \frac{0.526\,W \cdot time}{4.908 \cdot 10^{-19} J \cdot N_A} \qquad \text{eq. S17}$$

Because not all photons emitted by the laser are indeed absorbed by the sample, the transmittance of the latter needs to be taken into account. The transmittance is the percentage of photons passing through the solution at 405 nm and is defined in eq. S18, where A is the absorbance at the relevant wavelength.

$$T = 10^{-A} \qquad \text{eq. S18}$$

The number of photons absorbed by the solution (n_{abs}) is therefore in the following relationship with the number of photons emitted by the laser (n_{laser}).

$$n_{abs} = (1 - T) \cdot n_{laser} \qquad \text{eq. S18}$$

As ϕ_{decomp} was determined at I/I_0=0.9 (see above), the number of decomposed photosensitizer molecules at that point is equal to 90% of the initial concentration c_0 (eq. S19).

$$n_{decomp} = 0.9 \cdot V \cdot c_0 \qquad \text{eq. S19}$$

In eq. S19, the volume V is 0.003 L.

Table S4: Values needed to calculate the photo-decomposition quantum yield (ϕ_{decomp}).

ϕ_{decomp}.Photosensitizer	time	c_0	A	n_{abs}	n_{decomp}	ϕ_{decomp}
[Zn(*m*-L)$_2$]	284 s	11.8 μM	0.050	$5.46 \cdot 10^{-5}$	$3.17 \cdot 10^{-8}$	0.06%
[Zn(*p*-L)$_2$]	90 s	3.00 μM	0.052	$1.83 \cdot 10^{-5}$	$8.11 \cdot 10^{-9}$	0.04%
$[Ru(bpy)_3]^{2+}$	332 s	1.26 μM	0.050	$6.51 \cdot 10^{-5}$	$3.40 \cdot 10^{-9}$	0.005%

PHOTOINDUCED ELECTRON TRANSFER TO 1,2,4,5-TETRACYANOBENZENE

The [3]ILCT decays of the $[Zn(\textit{m}\text{-}L)_2]$ and $[Zn(\textit{p}\text{-}L)_2]$ complexes were monitored in the absence and in the presence of different concentrations of 1,2,4,5-tetracyanobenzene (TCB) in order to determine the rate constants for photoinduced electron transfer from the Zn^{II} complexes to TCB. In the case of the $Zn(\textit{m}\text{-}L)_2]$ complex, the decays of an excited-state absorption (ESA) signal at 350 nm (Figure 5a) was monitored (Figure S34. In the case of the $[Zn(\textit{p}\text{-}L)_2]$ complex, a ground-state bleach (GSB) recovery at 390 nm (Figure 5b) was followed (Figure S35). The respective data sets were normalized (and in the case of the GSB recovery data multiplied by a factor of -1 to obtain positive $\Delta OD/\Delta OD_0$ values), and then the data in Figure S35 were fitted with mono-exponential functions to obtain to the lifetimes used for the Stern-Volmer plots in the insets of Figure 7b and Figure S36b.

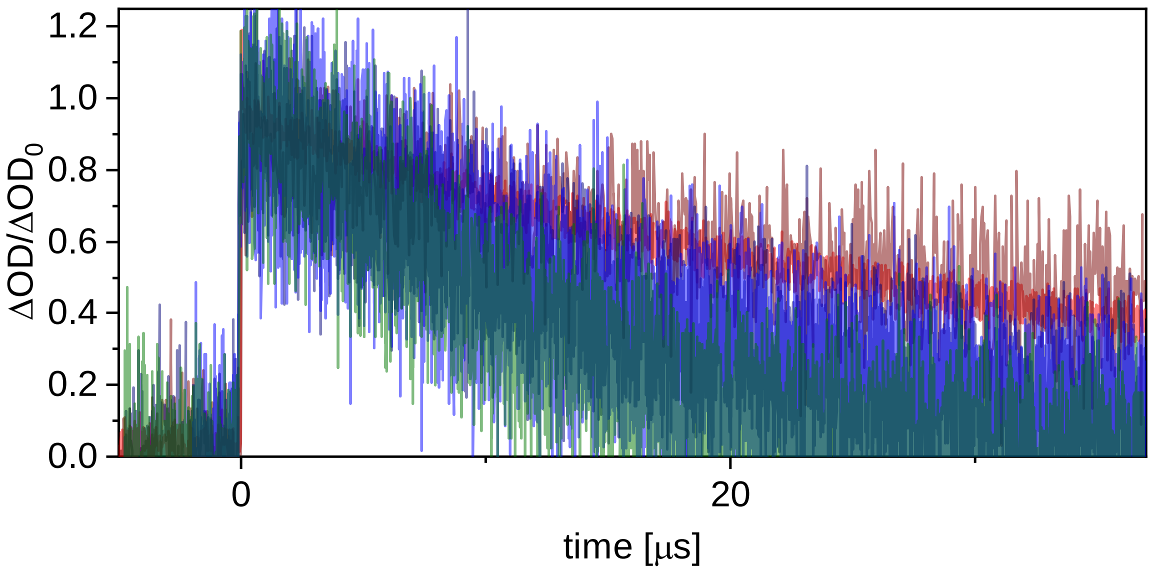

Figure S34: Decays of the excited state absorption signal of $[Zn(\textit{m}\text{-}L)_2]$ at 350 nm (Figure 5a) in the presence of different concentrations of TCB (red 0.5 µM, dark red 1.25 µM, dark blue 2.5 µM, blue 5 µM, dark green 10 µM) in deaerated toluene excited at 293 K. Excitation occurred at 420 nm with laser pulse energies of 12 mJ.

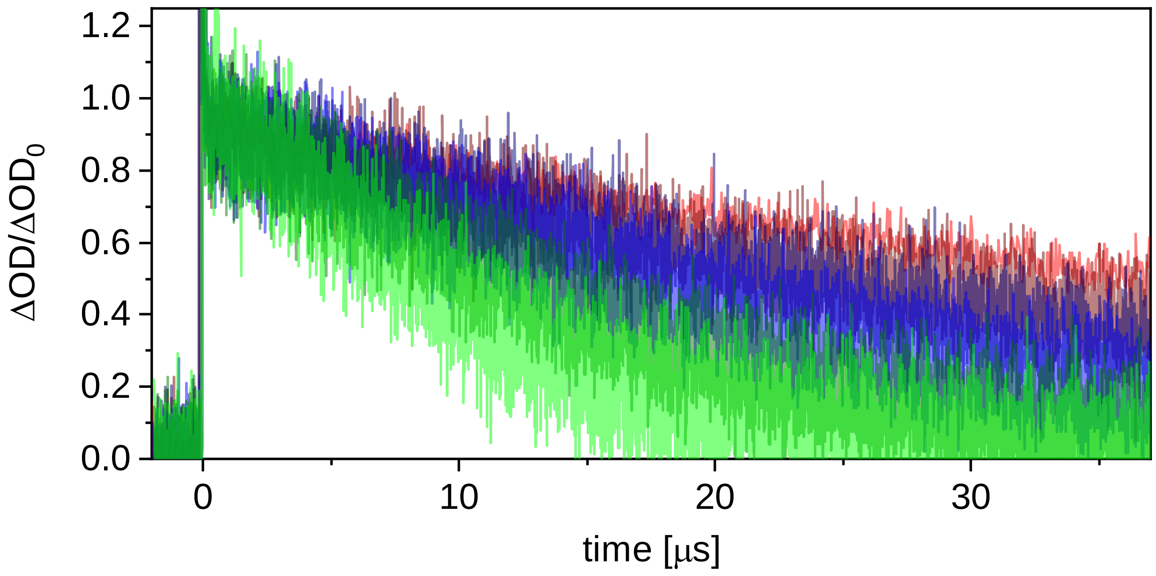

Figure S35: Decays of the [3]ILCT excited state of [Zn(*p*-L)$_2$] by monitoring the ground state bleach (GSB) recovery of this compound at 390 nm (Figure 5b) in the absence and in the presence of different concentrations of TCB (red 0 μM, dark red 1.25 μM, dark blue 2.5 μM, blue 5 μM, dark green 10 μM, green 15 μM) in deaerated toluene at 293 K. Excitation occurred at 420 nm with a laser energy of 12 mJ.

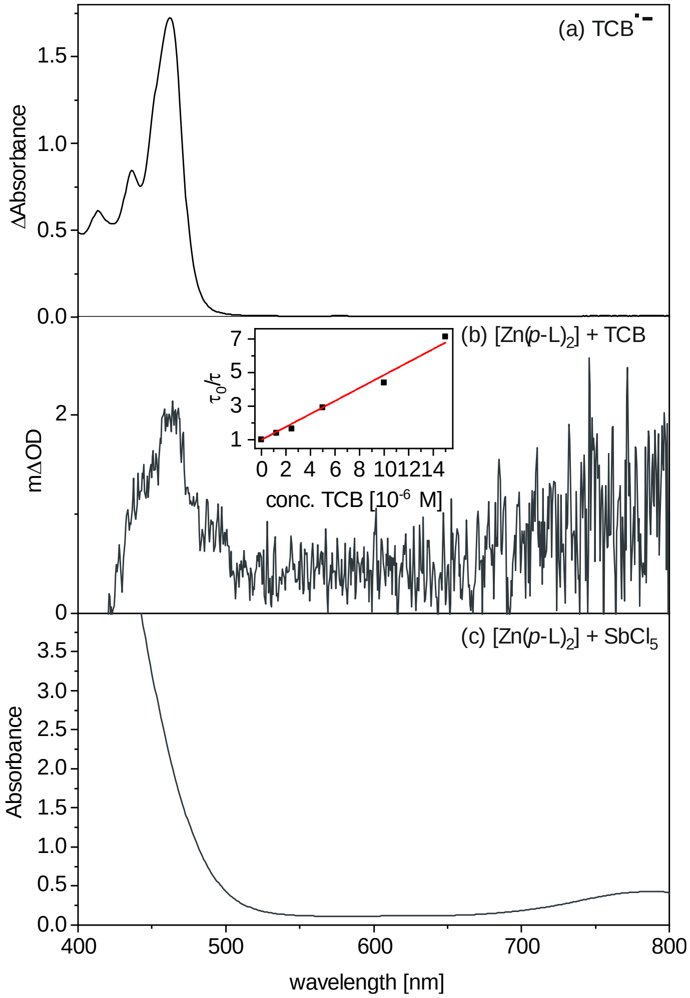

Figure S36: (a) UV-Vis difference spectrum obtained upon electrochemical reduction of 1,2,4,5-tetracyanobenzene (TCB, 2.5 mM) in deaerated MeCN in the presence of $N(nBu)_4PF_6$ (0.1 M) as supporting electrolyte, obtained by applying a voltage of -0.55 V *vs.* SCE (b) transient absorption of $[Zn(p\text{-}L)_2]$ (7.2 μM) and TCB (2.5 mM) in deaerated toluene at 293 K excited at 420 nm with a laser pulse energy of 12 mJ. Inset: Stern-Volmer-type plot for the oxidative excited-state quenching of $[Zn(p\text{-}L)_2]$ by TCB in deaerated toluene, based on the monitoring of a ground state bleach at 390 nm (k_q=6.29·10^9 M^{-1}s^{-1}), see Figure S35. (c) Absorption spectrum of $[Zn(p\text{-}L)_2]^{\cdot+}$ in deaerated CH_2Cl_2 at 293 K after chemical oxidation of a $[Zn(p\text{-}L)_2]$ solution (79 μM) with $SbCl_5$ (1 eq.).

UPCONVERSION

A deaerated toluene solution of the sensitizer [Zn(*m*-L)$_2$] (30 μM) and the annihilator 1,4-bis((trimethylsilyl)ethynyl)naphthalene (2 mM) was prepared. The prompt sensitizer ^{1}ILCT emission as well as the upconverted annihilator emission were measured simultaneously in a laser flash photolysis experiment with a long integration time (100 μs) and without a time delay (manifesting in some laser stray light, blue trace in Fig. 9b), using an excitation wavelength of 430 nm and a laser pulse energy of 16 mJ. These conditions allow us to detect the entire prompt as well as the entire upconverted emission in the same experiment. In a second measurement, a time delay (1 μs) was applied (red trace in Fig. 9b). Under these detection conditions, the prompt sensitizer ^{1}ILCT emission is undetectable, because the [Zn(*m*-L)$_2$] fluorescence has decayed completely after 1 μs. The stray laser light is also not visible anymore, because of the time delay. With a deaerated solution containing only the sensitizer [Zn(*m*-L)$_2$] but no annihilator, the prompt emission was measured using the exact same instrumental settings as for the first measurement (0 ns delay, 100 μs integration, black trace in Fig. 9b).

The quantum yield of the ^{1}ILCT fluorescence of [Zn(*m*-L)$_2$] (ϕ_{fluo}=50%) under the conditions relevant for the sTTA-UC experiment is known and is assumed to remain constant upon addition of the annihilator, because the annihilator only quenches the ^{3}ILCT state, but not the luminescent ^{1}ILCT state. The prompt emissions represented by the black and the blue traces in Figure 8b were both normalized. The upconverted emission represented by the red trace was scaled to the match the intensity of the upconverted emission (blue trace in Figure 8b) at 370 nm. This scaling has the advantage that the red and the black trace can be used directly to determine the integrated emission intensities of the upconverted (I_{UC}) and the prompt ^{1}ILCT emission (I_{fluo}). This procedure seemed meaningful, because in the blue trace, the signals of the prompt and the upconverted emission, as well as the stray laser light overlap, which would make it very tricky to use the blue trace for the determination of the relevant integrals. The emission spectra were then converted from wavelengths to the wavenumber scale prior to determining the actual integrated emission intensities I_{UC} and I_{fluo}. This procedure yielded integrals for the prompt emission (black trace in Figure 8b) of I_{fluo}=109 and I_{UC}=1.59 for the upconverted emission (red trace in Figure 8b). These values were then used to estimate the upconversion quantum yield ϕ_{UC} according to equation S20.

$$\phi_{UC} = \frac{I_{UC}}{I_{fluo}} \cdot \phi_{fluo} = \frac{1.59}{109} \cdot 50\% = 0.73\% \qquad \text{eq. S20}$$

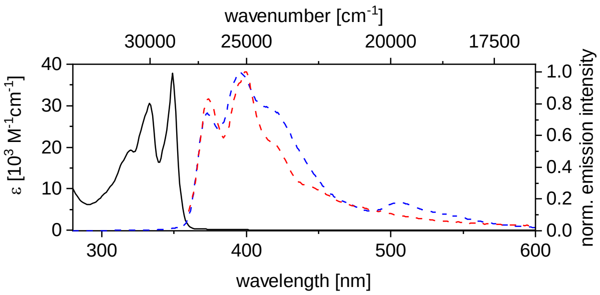

Figure S37: UV-Vis absorption (black solid trace, 50 μM) and normalized emission spectra (dashed traces, blue: 5.6 mM, red: 150 μM) of $(TMS)_2$napht (excited at 350 nm) in deaerated toluene at 293 K.

NMR SPECTRA

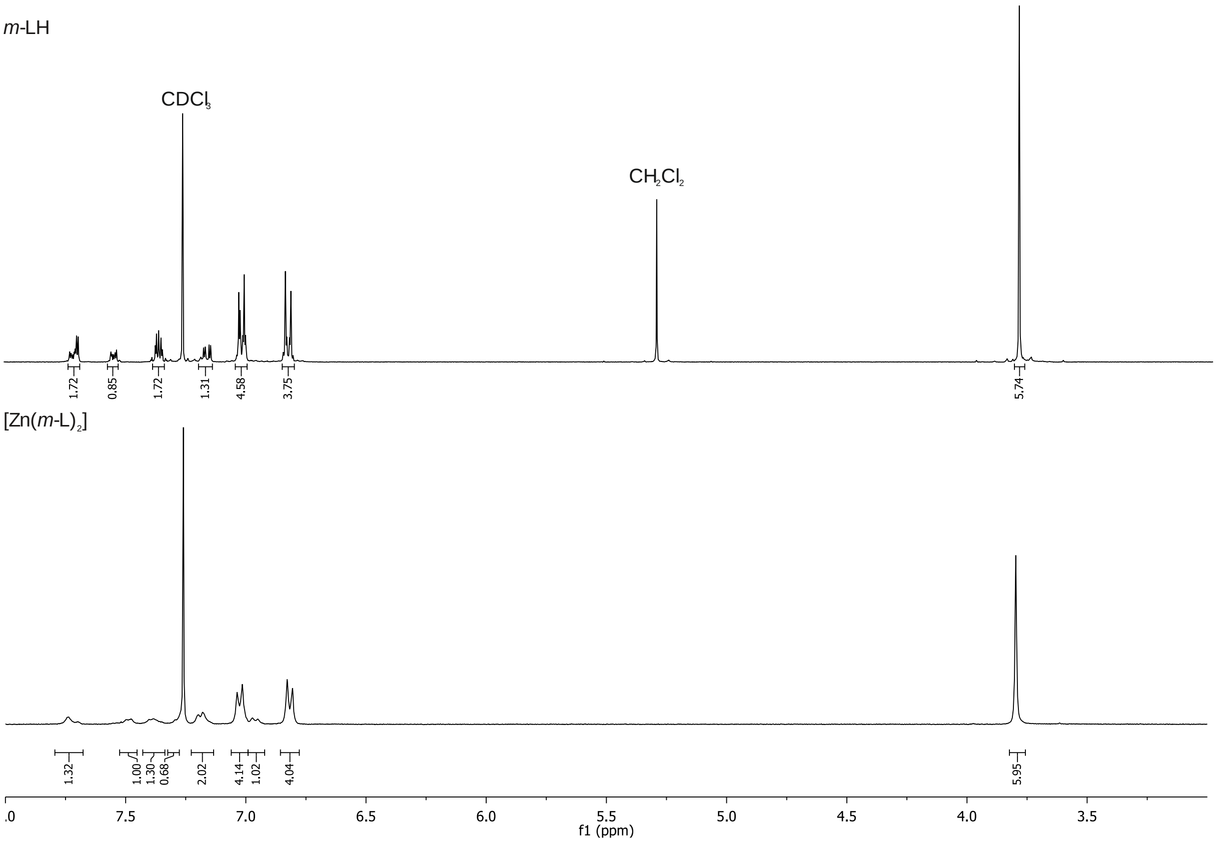

Figure S38: ^{1}H NMR spectra of *m*-LH and [Zn(*m*-L)$_2$] in $CDCl_3$.

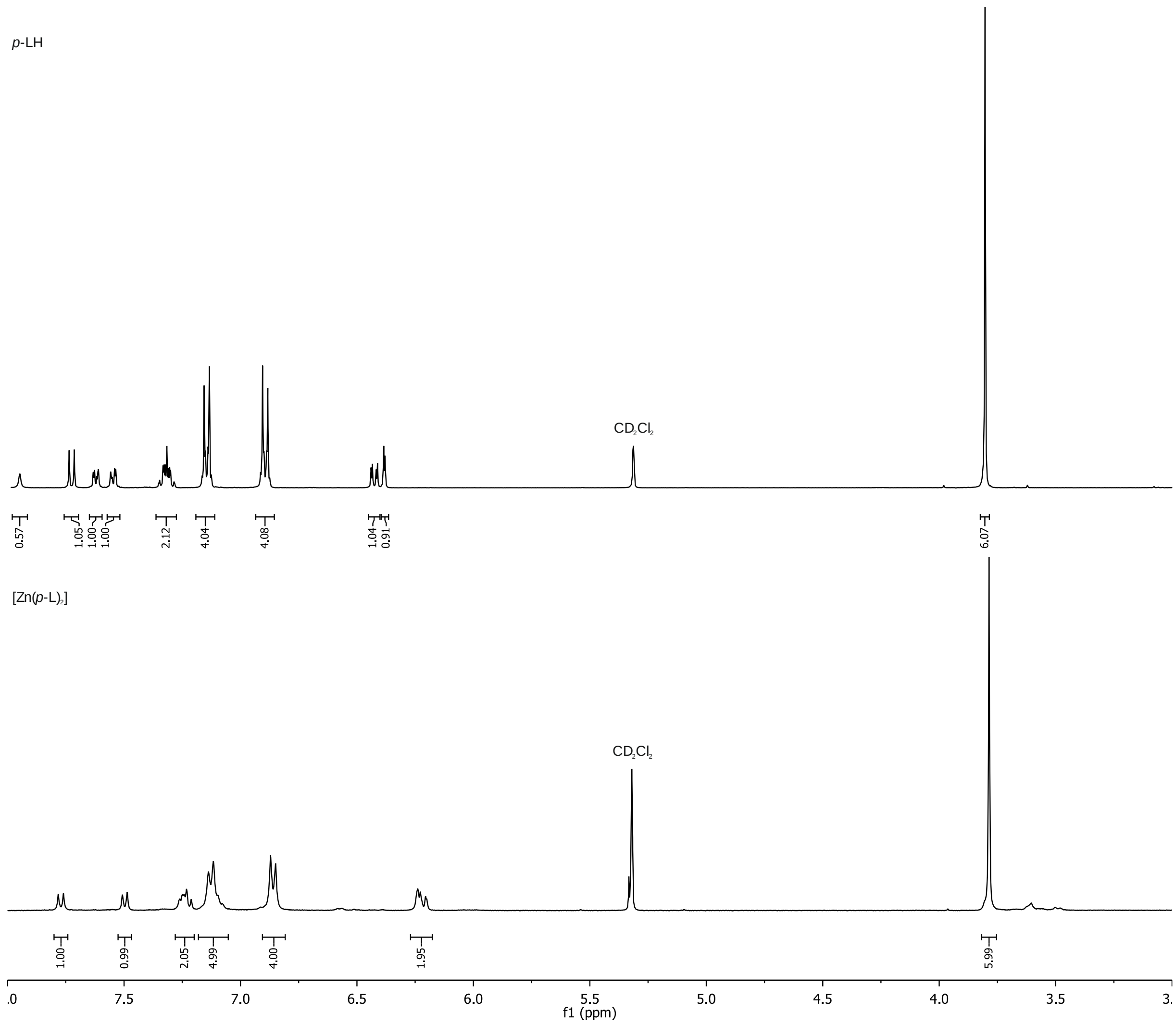

Figure S39: ^{1}H NMR spectra of *p*-LH and [Zn(*p*-L)$_2$] in CD_2Cl_2.

MASS SPECTRA

MALDI-TOF MS

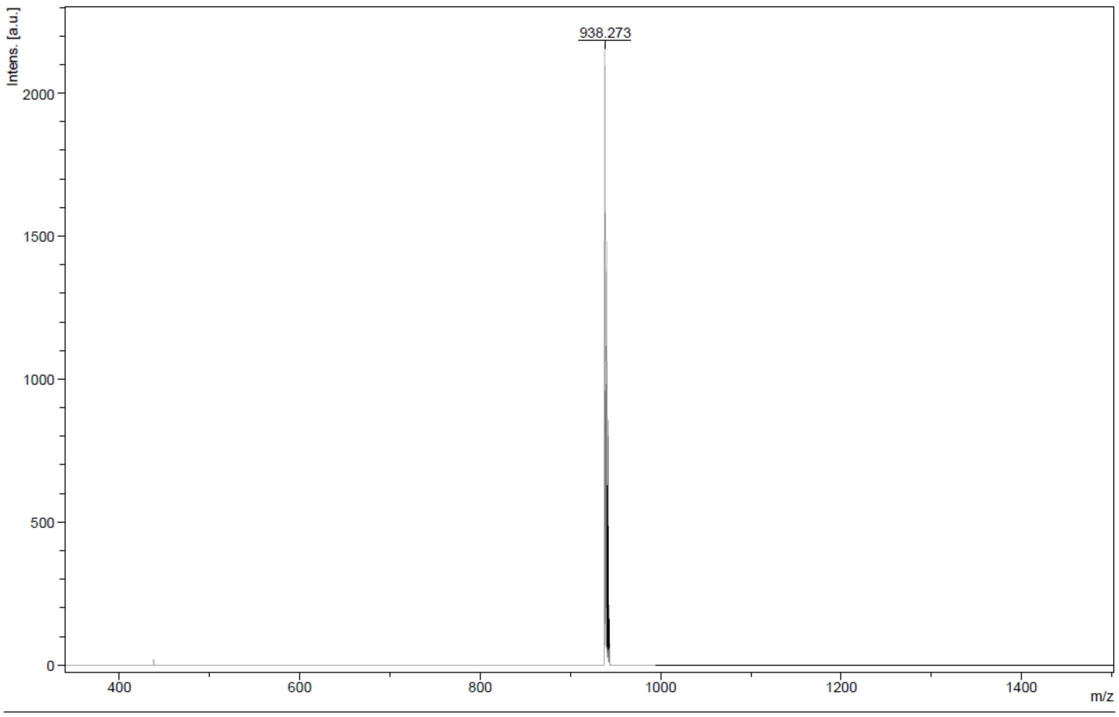

Figure S40: MALDI-TOF MS of [Zn(*m*-L)$_2$].

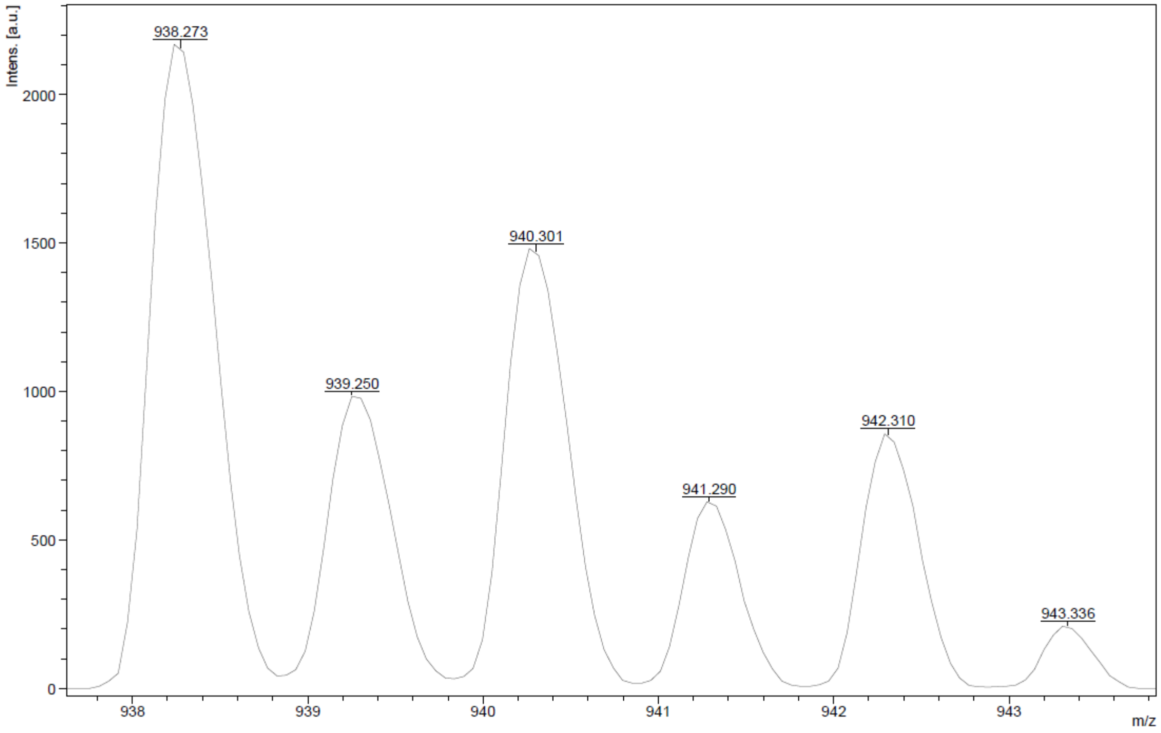

Figure S41: Zoom of the MALDI-TOF MS data of [Zn(*m*-L)$_2$] from Figure S40.

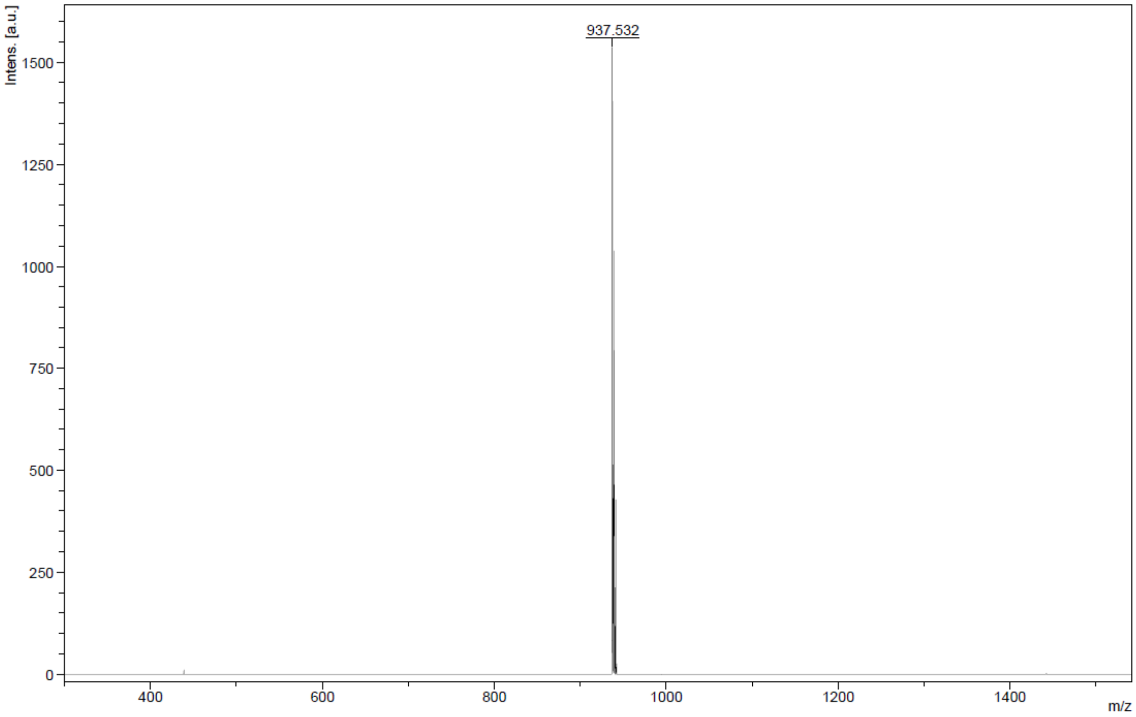

Figure S42: MALDI-TOF MS of [Zn(*p*-L)$_2$].

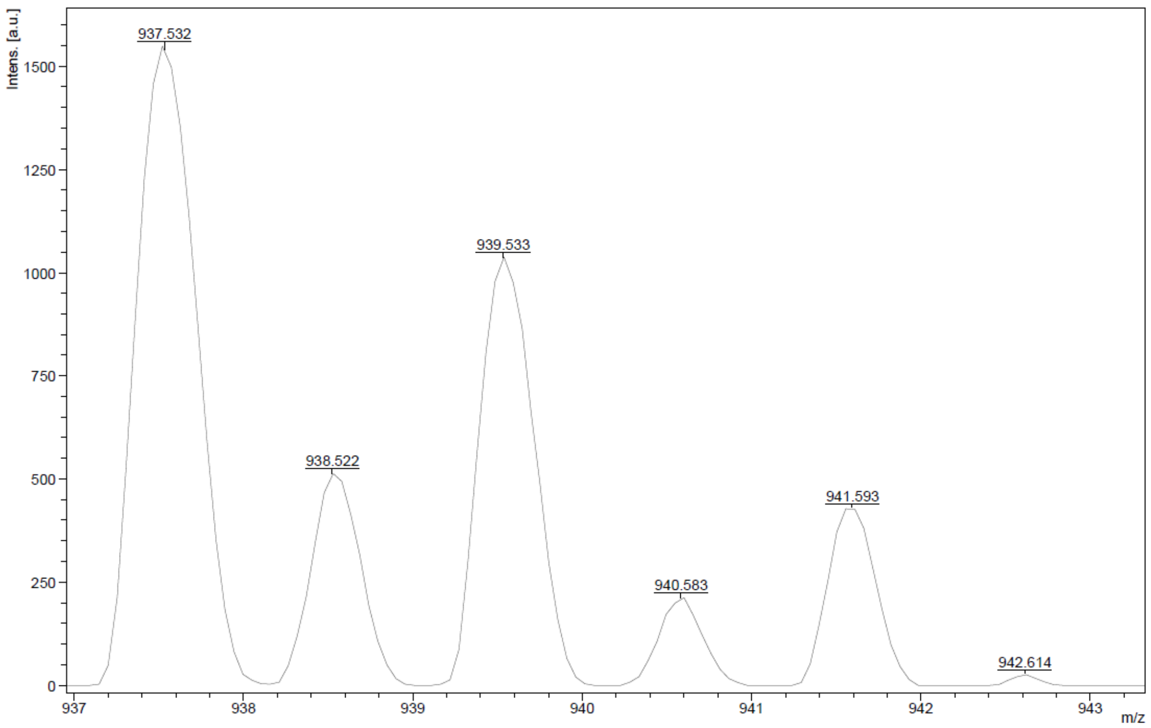

Figure S43: Zoom of the MALDI-TOF MS of [Zn(*p*-L)$_2$] from Figure S42.

TD-DFT CALCULATIONS

Quantum chemical calculations were performed to investigate the electronic structures of the two isomeric zinc(II) complexes using DFT. In a first step, the ground and lowest triplet excited state geometries were optimized using the Gaussian 09 software[15] with the PBE1PBE hybrid functional[16] and the def2SV(P) basis[17] set in vacuum to obtain full geometrical freedom. The optimized geometries were confirmed to be the local minima by vibrational frequency analysis (only positive eigenvalues). In a second step, the S_0 and sixty vertical energies (S_{1-60}) were computed by single point calculations on the vacuum ground-state geometry in CH_2Cl_2, employing the polarizable continuum model (PCM) and the same level of theory. In a third step, the energy of the lowest triplet state was calculated using the optimized triplet state geometry in CH_2Cl_2 (PCM) to estimate the energy differences between the lowest singlet and triplet excited states (ΔE_{ST}).

Natural transition orbitals (NTO) with a contribution greater than 5% were of main interest. Ultimately, only the first two transitions from S_0 to S_1 and from S_0 to S_2 were considered, as these are the only transitions to exhibit sizeable oscillator strengths (>0.005) and transition energies below 3.54 eV (350 nm). All other transitions were either calculated at higher transition energies (comprised mainly of π->π* transitions) or featured considerably weaker oscillator strengths.

The transitions $S_0 \rightarrow S_1$ and $S_0 \rightarrow S_2$ of [Zn(*m*-L)$_2$] both show one dominant transition (96%), located solely on the opposite ligand (Figure S44) yet are otherwise essentially identical. The energy difference between these two vertical transitions is only 0.0012 eV. Consequently, these two transitions are considered identical, in line with the expectation that both coordinated ligands are involved and are in principle indistinguishable from each other.

For the [Zn(*p*-L)$_2$] complex, qualitatively analogous but quantitatively different results are obtained. In this case, the $S_0 \rightarrow S_1$ transition involves NTOs with a 60% contribution corresponding to the HOMO-LUMO transition on one ligand and a 39% contribution corresponding to an electronic transition between HOMO – 1 and LUMO + 1 located on the other ligand (Figure S45). For the transition $S_0 \rightarrow S_2$, essentially the same results were obtained, but now with reversed ligand contributions (Figure S46).

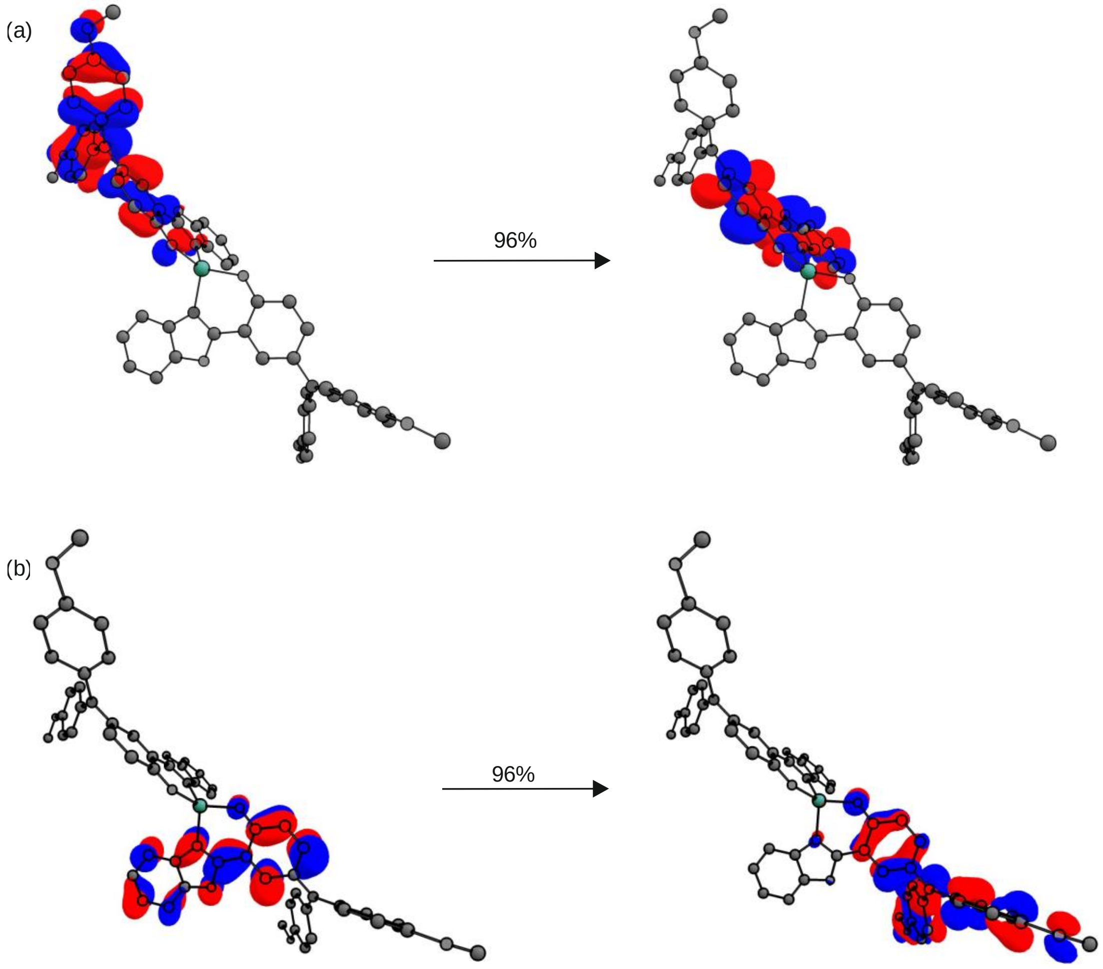

Figure S44: (a) NTOs for the transition $S_0 \rightarrow S_1$ from the HOMO to the LUMO of [Zn(*m*-L)$_2$] calculated by DFT with the probability indicated on the arrow. (b) NTOs for the transition $S_0 \rightarrow S_2$ of [Zn(*m*-L)$_2$] calculated by DFT with the probability indicated on the arrow. The zinc(II) cation is colored in green.

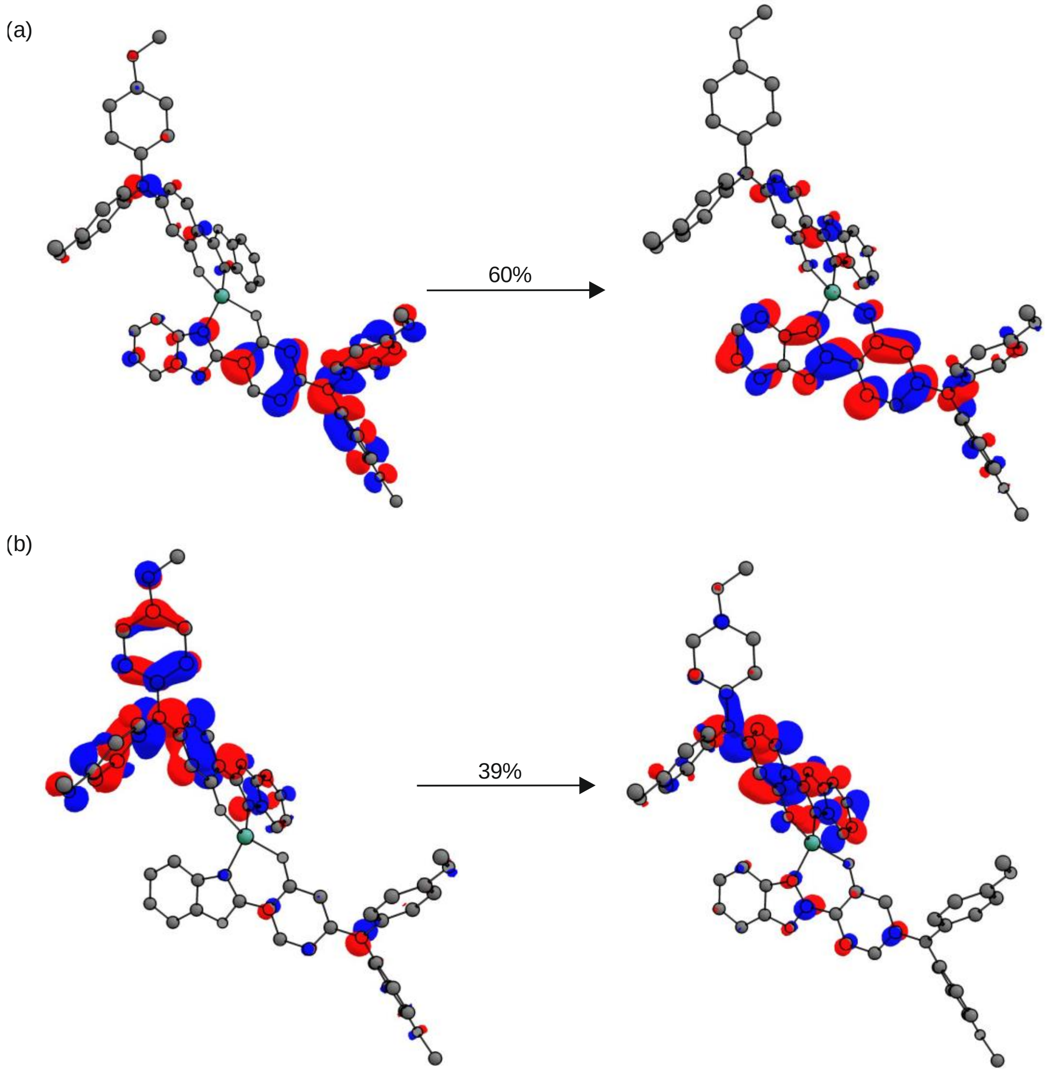

Figure S45: (a) NTOs for the transition $S_0 \rightarrow S_1$ from HOMO to LUMO of [Zn(*p*-L)$_2$] calculated by DFT with the probability indicated on the arrow. (b) NTOs for the transition $S_0 \rightarrow S_1$ from HOMO – 1 to LUMO + 1 of [Zn(*p*-L)$_2$] calculated by DFT with the probability indicated on the arrow. The zinc(II) cation is colored in green.

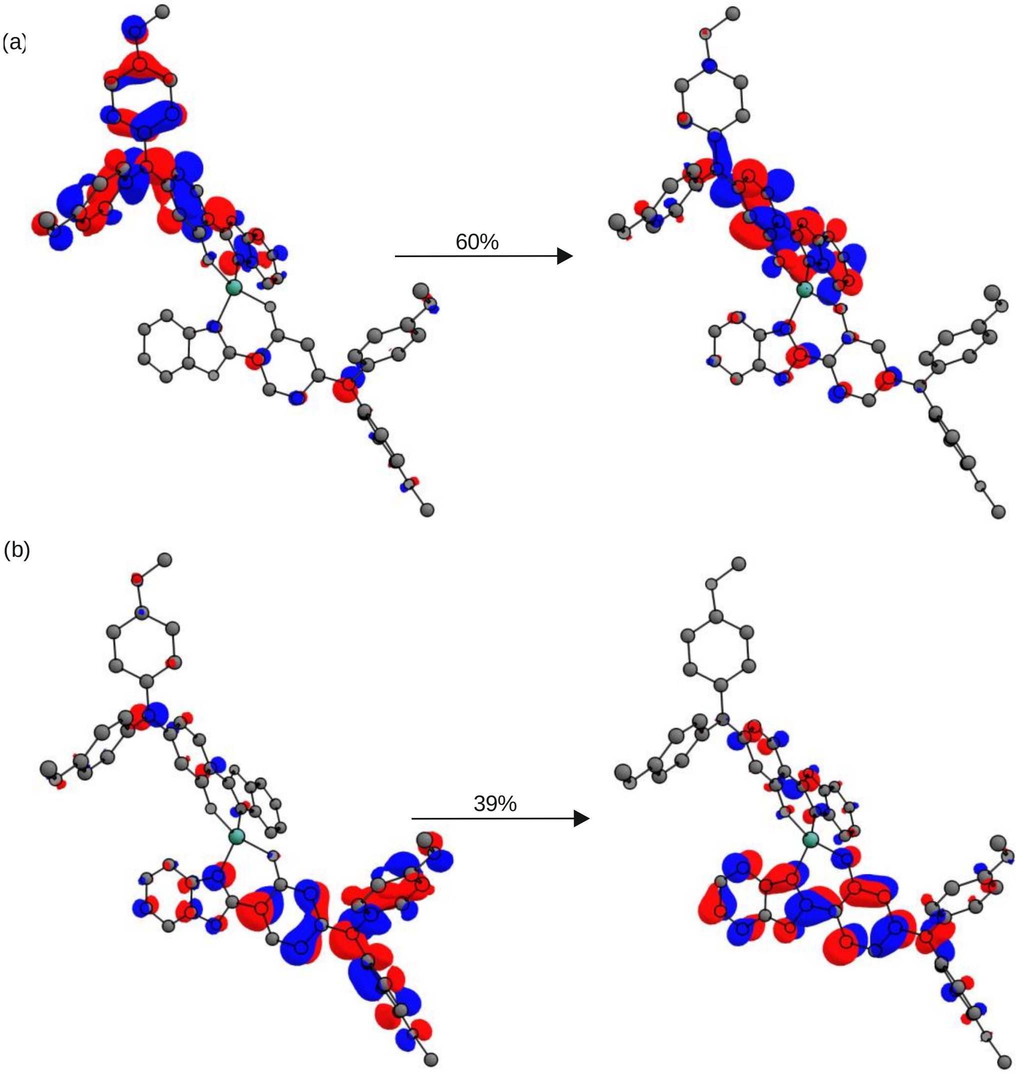

Figure S46: (a) NTOs for the transition $S_0 \rightarrow S_2$ from HOMO to LUMO of $[\mathrm{Zn}(p\text{-}\mathrm{L})_2]$ calculated by DFT with the probability indicated on the arrow. (b) NTOs for the transition $S_0 \rightarrow S_2$ from HOMO – 1 to LUMO + 1 of $[\mathrm{Zn}(p\text{-}\mathrm{L})_2]$ calculated by DFT with the probability indicated on the arrow. The zinc(II) cation is colored in green.

GROUND STATE GEOMETRY OF [ZN(*P*-L)2] IN VACUUM

C 5.38598900 -0.80855000 1.32845300
C 5.13847900 0.34494400 0.53182400
C 3.87703800 0.51887500 -0.02997600
C 2.81622000 -0.40189500 0.15312000
C 3.08994700 -1.56472900 0.96331700
C 4.38138200 -1.72227100 1.52562600
C 2.09448000 -2.54830200 1.23176500
N 0.83620300 -2.60395200 0.82212100
C 0.28890000 -3.74661700 1.37623400
C 1.29357200 -4.36708100 2.12298400
O 2.41584100 -3.60349400 2.02049900
C -0.98613700 -4.30615800 1.30735300
C -1.19375300 -5.49709000 2.00384900
C -0.16965000 -6.10733000 2.74426200
C 1.10909200 -5.54767600 2.82092400
O 1.68855700 -0.16199300 -0.42140000
N 6.15008500 1.28581500 0.34102200
C 5.84675000 2.63984300 0.04650600
C 7.51463400 0.90842800 0.38739000
C 7.97913600 -0.21661900 -0.31318000
C 9.31782700 -0.57513700 -0.27245900
C 10.24160100 0.19337100 0.45102700
C 9.79079700 1.32620500 1.13796900
C 8.43859000 1.66831500 1.10870100
C 6.47138700 3.29261100 -1.02701100
C 6.19954200 4.62393200 -1.30360800
C 5.28000300 5.34073000 -0.52453700
C 4.64866600 4.69579200 0.54545900
C 4.94278800 3.36223800 0.82880500
O 5.07112800 6.62734600 -0.87547800
C 4.15625000 7.38857400 -0.14154200
O 11.52394700 -0.22779200 0.42295400
C 12.48962600 0.49789700 1.12682400
C -5.33898200 -0.42084800 -1.70046000
C -5.17800000 0.08104900 -0.37800400
C -3.92317200 0.01897100 0.21980000
C -2.78558700 -0.52501100 -0.42718200
C -2.96969800 -1.01936500 -1.77052200
C -4.25722300 -0.94795000 -2.35948400
C -1.89318200 -1.56728400 -2.52656500
N -0.62409800 -1.73100400 -2.18497100
C 0.01245300 -2.28800600 -3.27905100
C -0.94993000 -2.45391600 -4.27813800
O -2.13565500 -1.99718800 -3.78956000
C 1.33624800 -2.66167000 -3.50593300
C 1.63549300 -3.20605300 -4.75515600
C 0.65283400 -3.36986500 -5.74356500
C -0.67472000 -2.99129400 -5.52332200
O -1.66550100 -0.53820300 0.20809000
N -6.27651700 0.60761000 0.30168500
C -6.32220700 0.62184700 1.71896600
C -7.34103400 1.22302600 -0.40160900
C -8.67396300 0.92399600 -0.07659200
C -9.72223400 1.53640500 -0.74513800
C -9.47259200 2.45792200 -1.77323900
C -8.14768000 2.75988200 -2.10636300
C -7.09780900 2.15447000 -1.41403600
C -6.08090600 -0.54740400 2.45819800
C -6.14530100 -0.53648600 3.84217900
C -6.47217900 0.64035500 4.53324000
C -6.72664100 1.80667300 3.80435200
C -6.64148500 1.79118700 2.41119800
O -6.52030900 0.54994500 5.87914800
C -6.82820400 1.69294400 6.62286100
O -10.55664200 2.99413300 -2.37321500

C -10.37007900 3.92064300 -3.40344000
Zn 0.05522400 -1.17306200 -0.36818800
H 6.36072000 -0.95157000 1.79746800
H 3.65686800 1.38628900 -0.65425700
H 4.57120600 -2.59729700 2.15221800
H -1.78366100 -3.82223900 0.73822900
H -2.18127100 -5.96663400 1.97506100
H -0.37592300 -7.04040900 3.27628000
H 1.91495000 -6.00855800 3.39606300
H 7.27232900 -0.81505500 -0.89433800
H 9.68369200 -1.45103900 -0.81459500
H 10.48018900 1.94920500 1.71140600
H 8.09361000 2.55034400 1.65502200
H 7.18256600 2.74118000 -1.64795600
H 6.68299700 5.13692800 -2.13905000
H 3.93026700 5.22233600 1.17691200
H 4.44939200 2.86922600 1.67063600
H 4.13952400 8.38743000 -0.60336400
H 3.13683500 6.95786000 -0.18175500
H 4.46089200 7.48948000 0.91848800
H 13.44794200 -0.01864400 0.96542800
H 12.57594000 1.53743300 0.75456400
H 12.27500900 0.52328400 2.21304300
H -6.32035900 -0.40222000 -2.17704500
H -3.76333800 0.40418300 1.22817400
H -4.38754600 -1.34154100 -3.37064500
H 2.10076000 -2.52197500 -2.73776000
H 2.66358800 -3.51018500 -4.97190100
H 0.93113000 -3.80023100 -6.70977700
H -1.44947800 -3.10849900 -6.28399400
H -8.88080700 0.20014500 0.71631500
H -10.76147100 1.30642800 -0.49607800
H -7.91401800 3.47821500 -2.89482700
H -6.06592400 2.40848100 -1.67146800
H -5.83396300 -1.47267800 1.93097000
H -5.95588200 -1.44301100 4.42282400
H -6.98277500 2.74149400 4.30699000
H -6.83420700 2.70933400 1.84969000
H -6.80647800 1.39237500 7.68136600
H -6.08596800 2.49939000 6.46412900
H -7.83681000 2.08191800 6.38161300
H -11.37413200 4.21719200 -3.74281400
H -9.82951000 4.82191500 -3.05401200
H -9.81850300 3.48116800 -4.25740800

TRIPLET STATE GEOMETRY OF [ZN(*P*-L)2] IN VACUUM

```
C 5.39308000 -0.39641000 1.46394500
C 5.10954500 0.56864000 0.45688200
C 3.82991400 0.58676400 -0.13829700
C 2.82400300 -0.31219800 0.20991900
C 3.13153100 -1.32141200 1.25524400
C 4.44025900 -1.31227100 1.84521300
C 2.19295600 -2.26863900 1.68206000
N 0.90184400 -2.46758700 1.30506400
C 0.42981400 -3.50565000 2.04567200
C 1.47262800 -3.94717900 2.88800600
O 2.55804600 -3.17669000 2.65700300
C -0.82251100 -4.13597000 2.09096800
C -0.97215000 -5.19605600 2.98369200
C 0.07953700 -5.62256600 3.80984100
C 1.33503100 -4.99675500 3.77662100
O 1.67907700 -0.27203000 -0.38431000
N 6.09227300 1.50492500 0.10833500
C 5.74394400 2.84154100 -0.15961100
C 7.42938800 1.09050000 -0.00098400
C 7.74234700 -0.16940300 -0.54835000
C 9.05644400 -0.59340800 -0.63782400
C 10.10593300 0.22571100 -0.19004900
C 9.80411800 1.48300300 0.35210000
C 8.48076400 1.90340800 0.44821300
C 6.37246800 3.56062700 -1.19391800
C 6.02187000 4.87338000 -1.45560100
C 5.02197300 5.51123600 -0.70286900
C 4.38538100 4.79945200 0.32264900
C 4.74720300 3.48233400 0.58914000
O 4.74477200 6.78535700 -1.03669300
C 3.73964400 7.46862500 -0.34112900
O 11.34955900 -0.27167200 -0.32126700
C 12.44054500 0.49598000 0.10334400
C -5.28827600 -0.78217700 -1.64713300
C -5.13848500 -0.03548400 -0.44450800
C -3.89268100 0.01013500 0.17204600
C -2.75186500 -0.65880500 -0.33988000
C -2.92516700 -1.40293600 -1.56451200
C -4.20371200 -1.43596200 -2.17542200
C -1.84597700 -2.10033700 -2.18084200
N -0.58514400 -2.20889000 -1.79218600
C 0.05788500 -2.97939900 -2.74297400
C -0.89189000 -3.32645600 -3.70714300
O -2.07711800 -2.76730600 -3.33928500
C 1.37813200 -3.40967800 -2.86637500
C 1.68736300 -4.19401800 -3.97794000
C 0.71768200 -4.53559000 -4.93322400
C -0.60650700 -4.10276400 -4.81664300
O -1.64118000 -0.55653400 0.30118800
N -6.24133500 0.62068300 0.10529700
C -6.30949900 0.89937800 1.49367200
C -7.28562300 1.10326900 -0.72041700
C -8.62782800 0.88855400 -0.36753600
C -9.65669200 1.37401100 -1.15916800
C -9.37801100 2.07775200 -2.34044600
C -8.04394500 2.29359700 -2.70235200
C -7.01365700 1.81971500 -1.88862900
C -6.08329700 -0.11092600 2.44256200
C -6.16947100 0.15870700 3.79883900
C -6.50390800 1.44384000 4.25241500
C -6.74334800 2.45321300 3.31439100
C -6.63600000 2.17758100 1.95017900
O -6.57360500 1.60681500 5.59091900
C -6.88834400 2.86893300 6.10220600
O -10.44508000 2.50226700 -3.05099500
```

C -10.22872700 3.21148400 -4.23596400
Zn 0.08116400 -1.31071100 -0.10964900
H 6.36725800 -0.38467700 1.96007900
H 3.59116200 1.30998300 -0.92066100
H 4.66539000 -2.04125100 2.62515000
H -1.64271300 -3.79616800 1.45394100
H -1.93665700 -5.70898200 3.04475200
H -0.08222500 -6.45854100 4.49624100
H 2.16117400 -5.31562000 4.41573900
H 6.93290100 -0.80467700 -0.91671700
H 9.30618600 -1.56533900 -1.07082400
H 10.59371700 2.14097000 0.72040200
H 8.25535400 2.87674400 0.89145000
H 7.13554300 3.07052700 -1.80394200
H 6.49993500 5.43384400 -2.26301000
H 3.60543900 5.26284400 0.92989600
H 4.24870700 2.93582500 1.39309200
H 3.67962400 8.47232500 -0.78783400
H 2.75762900 6.96876000 -0.44562400
H 3.98103300 7.56835800 0.73467900
H 13.34082600 -0.10160900 -0.10369300
H 12.50890100 1.45223900 -0.45033100
H 12.39352500 0.70984400 1.18850400
H -6.26335400 -0.84858400 -2.13236900
H -3.74198600 0.58163400 1.08937300
H -4.32523100 -2.01754100 -3.09269200
H 2.13113800 -3.13366700 -2.12423400
H 2.71288000 -4.55083600 -4.11034400
H 1.00334900 -5.15254800 -5.78999600
H -1.37142500 -4.35783600 -5.55318100
H -8.85738400 0.33264900 0.54553700
H -10.70285100 1.20858400 -0.88869200
H -7.78766400 2.84380100 -3.61004100
H -5.97433600 2.00646600 -2.17225700
H -5.83021100 -1.11841100 2.10209200
H -5.99126600 -0.62334100 4.54144100
H -7.00468200 3.46555500 3.62914400
H -6.81692800 2.97473900 1.22406700
H -6.88392100 2.77225200 7.19855400
H -6.14075400 3.63081900 5.80678800
H -7.89165300 3.20703700 5.77662400
H -11.22281700 3.45042300 -4.64342300
H -9.67893700 4.15604400 -4.05634200
H -9.67170800 2.60956900 -4.98038700

GROUND STATE GEOMETRY OF [ZN(*M*-L)2] IN VACUUM

```
N -1.26040500 -0.77323600 -1.21567400
C -2.57513100 -0.66047500 -1.13818600
C -4.77672900 0.08385900 -0.37623500
C -3.36731200 0.11787100 -0.23256400
C -2.75474000 0.93939900 0.77577100
C -3.65033100 1.67668100 1.60425600
C -5.01649800 1.60377100 1.46332600
C -5.61219800 0.80226300 0.45847700
C -0.99857000 -1.62519200 -2.27445300
C -2.23040700 -2.01487300 -2.80463000
C 0.19077900 -2.09395600 -2.83118000
C 0.08031700 -2.96143600 -3.91723900
C -1.16846600 -3.34591700 -4.43173600
C -2.36291700 -2.87458500 -3.88159000
N -7.01905400 0.74193500 0.33300700
C -7.65431700 -0.50196300 0.13660200
C -7.76597200 1.93685800 0.31972300
C -9.03244000 2.00437100 0.92902800
C -9.76590000 3.18012600 0.92017300
C -9.25419900 4.34127300 0.32239500
C -7.99138300 4.28964900 -0.27504200
C -7.26667800 3.09634300 -0.28342700
C -8.69257500 -0.64793200 -0.79994100
C -9.32014300 -1.87081300 -0.98484600
C -8.91917800 -2.99955000 -0.25701900
C -7.87782200 -2.86948800 0.66822400
C -7.26556500 -1.63145400 0.86523300
C -9.23271100 -5.30133300 0.19458500
C -9.57841300 6.62400800 -0.20865700
C 5.55033100 0.82816800 -0.35405900
C 4.95318400 1.68424700 -1.31174000
C 3.58661300 1.76469200 -1.44652200
C 2.69228200 0.98200400 -0.65984600
C 3.30641300 0.10633000 0.30104800
C 4.71571200 0.06457500 0.44082400
C 2.51555500 -0.72037200 1.16429700
N 1.20105900 -0.83803400 1.23680600
C 0.94075300 -1.74572600 2.24864800
C 2.17334600 -2.16250200 2.75590600
C -0.24779000 -2.24419300 2.78075100
C -0.13572200 -3.16843400 3.81873200
C 1.11382900 -3.57920700 4.31060000
C 2.30745600 -3.07850100 3.78522600
N 6.95703300 0.75973000 -0.23267700
C 7.59280200 -0.49392200 -0.11555400
C 7.70842900 1.95098500 -0.18521200
C 7.21790800 3.08764500 0.48191000
C 7.94778000 4.26637900 0.51477800
C 9.20517900 4.34860400 -0.09883200
C 9.70807100 3.22089100 -0.75664200
C 8.95933100 2.04475000 -0.80635900
C 8.63918200 -0.69353700 0.80210100
C 9.26658200 -1.92521300 0.91214300
C 8.85795200 -3.01080300 0.12485200
C 7.80913100 -2.82777200 -0.78246400
C 7.19663400 -1.57982300 -0.90367200
C 9.16308500 -5.28436200 -0.45658300
C 11.09923700 5.67362500 -0.60563700
H 11.04983600 5.51988000 -1.70163900
O -3.20416200 -1.40020700 -2.07918500
O -1.48797900 1.04419400 0.98450300
O -9.58285000 -4.14967900 -0.51394600
O -10.03868700 5.44146300 0.37560500
O 3.14599100 -1.50918600 2.06345900
```

O 1.42513800 1.09704900 -0.86130900
O 9.52247000 -4.17404000 0.31087300
O 9.84649000 5.53542200 -0.00344100
Zn -0.03052000 0.22596500 0.03861400
H -5.66455400 2.17552500 2.13437500
H -3.19612500 2.29840200 2.38018200
H -5.21106500 -0.53868000 -1.16110200
H 1.16048300 -1.78262700 -2.43530800
H 0.98972900 -3.35013700 -4.38433800
H -1.20810700 -4.02759700 -5.28607200
H -3.34112500 -3.16060500 -4.27364200
H -9.44198000 1.11459300 1.41466400
H -10.75030900 3.23123500 1.39306500
H -7.55761600 5.17011100 -0.75379200
H -6.28699600 3.06973700 -0.76793400
H -9.01030900 0.21778300 -1.38717500
H -10.12907500 -1.98269500 -1.71175700
H -7.53861700 -3.72360200 1.25822500
H -6.46142800 -1.54175800 1.60056500
H -9.89312100 -6.10458100 -0.16638200
H -8.18008700 -5.59483500 0.01183100
H -9.38378100 -5.17724100 1.28501900
H -10.36379100 7.37867400 -0.04972600
H -8.64053100 6.97908600 0.26208300
H -9.40899800 6.50779000 -1.29724400
H 5.59977500 2.29310300 -1.95080100
H 3.13152000 2.42929700 -2.18548500
H 5.15107200 -0.60007000 1.18976900
H -1.21807100 -1.91249900 2.40327800
H -1.04443200 -3.58195400 4.26542800
H 1.15469300 -4.30547500 5.12730600
H 3.28620800 -3.38440700 4.16054400
H 6.24586200 3.03952500 0.97998600
H 7.56403100 5.14972700 1.03212300
H 10.68095300 3.24491100 -1.25219900
H 9.36037200 1.17756400 -1.33782300
H 8.96266100 0.13760800 1.43442000
H 10.08096300 -2.07912400 1.62517800
H 7.46360300 -3.64689600 -1.41675400
H 6.38593700 -1.44830300 -1.62537200
H 9.82486100 -6.10749100 -0.14649800
H 8.11149200 -5.58541500 -0.28041700
H 9.30437400 -5.10077300 -1.53992100
H 11.43107700 6.70400600 -0.40610600
H 11.84188000 4.97015500 -0.18013800

TRIPLET STATE GEOMETRY OF [ZN(*M*-L)2] IN VACUUM

```
N -1.25987400 -0.91500400 -1.19621900
C -2.58677900 -0.78460300 -1.08919900
C -4.76006600 0.05162100 -0.35804800
C -3.35510400 0.04819000 -0.22866900
C -2.72810300 0.91928900 0.75526500
C -3.59416600 1.73828400 1.52704000
C -4.96998800 1.71759800 1.39022300
C -5.57398900 0.86435000 0.43640100
C -1.02987800 -1.82556000 -2.20036100
C -2.27834100 -2.23941700 -2.68702100
C 0.14454500 -2.34187900 -2.75415500
C 0.00710400 -3.27232700 -3.78690700
C -1.25007000 -3.67568400 -4.25608800
C -2.43279400 -3.15875800 -3.70789500
N -6.96899300 0.83521700 0.29104600
C -7.63148200 -0.37554600 0.00556800
C -7.71679200 2.02350300 0.41170600
C -8.94775000 2.03393100 1.09202200
C -9.68219600 3.20229400 1.20791100
C -9.20668900 4.40429600 0.66019900
C -7.97964900 4.40404700 -0.01475200
C -7.25156500 3.22254600 -0.14133200
C -8.68288000 -0.41910500 -0.92725300
C -9.33751600 -1.60860300 -1.20096500
C -8.95593700 -2.79908300 -0.56264500
C -7.90630500 -2.76593400 0.36407100
C -7.26083000 -1.56400300 0.64639100
C -9.30786600 -5.12583600 -0.30256200
C -9.56529000 6.71860100 0.31263600
C 5.51350100 0.89285200 -0.35066400
C 4.90641200 1.78984200 -1.26113200
C 3.53006800 1.81511300 -1.39375100
C 2.66690700 0.95852600 -0.66067900
C 3.29706200 0.04302900 0.28010900
C 4.70219900 0.04242500 0.40661600
C 2.53166600 -0.82982800 1.10262900
N 1.20505400 -0.96734600 1.20606500
C 0.97852100 -1.92302100 2.16819200
C 2.22862000 -2.35652800 2.63306700
C -0.19393400 -2.46585900 2.70035300
C -0.05295700 -3.44198400 3.68955800
C 1.20578500 -3.86417400 4.13743400
C 2.38656600 -3.32097400 3.61086300
N 6.90850600 0.85859500 -0.20956800
C 7.57262700 -0.36342900 0.02007600
C 7.65657800 2.05017400 -0.29148800
C 7.19421100 3.23068700 0.31835000
C 7.92685600 4.40275700 0.23516500
C 9.15285300 4.43470500 -0.44811000
C 9.62507000 3.26200800 -1.05039100
C 8.87677000 2.08835600 -0.97587100
C 8.62303400 -0.44833800 0.95118500
C 9.27794600 -1.64856300 1.17195300
C 8.89781500 -2.80938600 0.48014100
C 7.84942500 -2.73514600 -0.44537300
C 7.20350800 -1.52185200 -0.67399800
C 9.24892700 -5.12269900 0.11901300
C 11.02095600 5.71606600 -1.13618400
H 10.91981000 5.48784900 -2.21492900
O -3.23878300 -1.58291000 -1.98437000
O -1.46262700 0.97261100 0.96906300
O -9.64580400 -3.90637600 -0.90001100
O -9.98725500 5.48924900 0.83135300
O 3.18665100 -1.66716200 1.95917200
```

O 1.40099600 1.01896400 -0.87018400
O 9.58797000 -3.93041300 0.76838700
O 9.79549000 5.61905900 -0.46831800
Zn -0.02902700 0.07380900 0.02906100
H -5.59716500 2.34324600 2.02982200
H -3.11476600 2.37930600 2.27149200
H -5.21817800 -0.58748100 -1.11477000
H 1.12455600 -2.02030800 -2.39323300
H 0.90627500 -3.69584700 -4.24436500
H -1.31248300 -4.40584900 -5.06801000
H -3.42063300 -3.45925000 -4.06377400
H -9.32264500 1.10903800 1.53806500
H -10.63691100 3.21703600 1.73989800
H -7.58201000 5.31732100 -0.46175200
H -6.30248700 3.22899700 -0.68350600
H -8.97997600 0.49685900 -1.44456900
H -10.15175300 -1.64866600 -1.92904600
H -7.58689400 -3.66991600 0.88634300
H -6.45227700 -1.54520700 1.38152400
H -9.98718400 -5.87993600 -0.72759600
H -8.26324400 -5.41667700 -0.52619000
H -9.44601200 -5.09718100 0.79563900
H -10.34388900 7.45086900 0.57405400
H -8.60397800 7.04272000 0.75608600
H -9.45998300 6.68492900 -0.78903000
H 5.53099100 2.44640200 -1.87164000
H 3.04825300 2.49066800 -2.10536300
H 5.16270900 -0.63065100 1.13174200
H -1.17520300 -2.12971000 2.35654000
H -0.95054600 -3.88714500 4.12922700
H 1.27099300 -4.63018700 4.91539300
H 3.37560100 -3.63552300 3.95092400
H 6.24883500 3.21537600 0.86674800
H 7.57425100 5.32151200 0.71080400
H 10.57086100 3.24999000 -1.59572700
H 9.24706900 1.18254800 -1.46310900
H 8.91848700 0.44371400 1.50959500
H 10.09075100 -1.72124200 1.89913500
H 7.53084800 -3.61496600 -1.00788300
H 6.39520200 -1.47047900 -1.40786800
H 9.92745900 -5.89508300 0.51121700
H 8.20399800 -5.42182500 0.32987000
H 9.38710600 -5.04701500 -0.97697000
H 11.35658700 6.75778300 -1.02224500
H 11.78272200 5.04350300 -0.69624400

References

(1) Fulmer, G. R.; Miller, A. J. M.; Sherden, N. H.; Gottlieb, H. E.; Nudelman, A.; Stoltz, B. M.; Bercaw, J. E.; Goldberg, K. I. NMR Chemical Shifts of Trace Impurities: Common Laboratory Solvents, Organics, and Gases in Deuterated Solvents Relevant to the Organometallic Chemist. *Organometallics* **2010**, *29*, 2176–2179, 10.1021/om100106e.

(2) Affeldt, R. F.; De Amorim Borges, A. C.; Russowsky, D.; Severo Rodembusch, F. Synthesis and Fluorescence Properties of Benzoxazole-1,4-Dihydropyridine Dyads Achieved by a Multicomponent Reaction. *New J. Chem.* **2014**, *38*, 4607–4614, 10.1039/c4nj00777h.

(3) Sakai, Y.; Sagara, Y.; Nomura, H.; Nakamura, N.; Suzuki, Y.; Miyazaki, H.; Adachi, C. Zinc Complexes Exhibiting Highly Efficient Thermally Activated Delayed Fluorescence and Their Application to Organic Light-Emitting Diodes. *Chem. Commun.* **2015**, *51*, 3181–3184, 10.1039/c4cc09403d.

(4) Ou, Y. P.; Zhang, J.; Hu, Y.; Yin, J.; Chi, C.; Liu, S. H. Oxidized Divinyl Oligoacene-Bridged Diruthenium Complexes: Bridged Localized Radical Characters and Reduced Aromaticity in Bridge Cores. *Dalton Trans.* **2020**, *49*, 16877–16886, 10.1039/d0dt02883e.

(5) Montalti, M.; Credi, A.; Prodi, L.; Gandolfi, M. T. *Handbook of Photochemistry*, 3rd Ed.; CRC/Taylor & Francis: Boca Raton, 2006.

(6) Suzuki, K.; Kobayashi, A.; Kaneko, S.; Takehira, K.; Yoshihara, T.; Ishida, H.; Shiina, Y.; Oishi, S.; Tobita, S. Reevaluation of Absolute Luminescence Quantum Yields of Standard Solutions Using a Spectrometer with an Integrating Sphere and a Back-Thinned CCD Detector. *Phys. Chem. Chem. Phys.* **2009**, *11*, 9850–9860, 10.1039/b912178a.

(7) Brannon, J. H.; Magde, D. Absolute Quantum Yield Determination by Thermal Blooming. Fluorescein. *J. Phys. Chem.* **1978**, *82*, 705–709, 10.1021/j100495a018.

(8) Demas, J. N.; Crosby, G. A. Quantum Efficiencies on Transition Metal Complexes. II. Charge-Transfer Luminescence. *J. Am. Chem. Soc.* **1971**, *93*, 2841–2847.

(9) Bolletta, F.; Maestri, M.; Balzani, V. Efficiency of the Intersystem Crossing from the Lowest Spin-Allowed to the Lowest Spin-Forbidden Excited State of Some Chromium(III) and Ruthenium(II) Complexes. *J. Phys. Chem.* **1976**, *80*, 2499–2503, 10.1021/j100563a014.

(10) Müller, P.; Brettel, K. $[Ru(Bpy)_3]^{2+}$ as a Reference in Transient Absorption Spectroscopy: Differential Absorption Coefficients for Formation of the Long-Lived ^{3}MLCT Excited State. *Photochem. Photobiol. Sci.* **2012**, *11*, 632–636, 10.1039/c2pp05333k.

(11) Ledger, M. B.; Salmon, G. A. Triplet-Triplet Extinction Coefficients of Anthracene and 9-Bromoanthracene Determined by a Ground State Depletion Method. *J. Chem. Soc., Faraday Trans. 2* **1976**, *72*, 883–886, 10.1039/F29767200883.

(12) Heckmann, A.; Lambert, C. Organic Mixed-Valence Compounds: A Playground for Electrons and Holes. *Angew. Chem. Int. Ed.* **2012**, *51*, 326–392, 10.1002/anie.201100944.

(13) Aranzaes, J. R.; Daniel, M. C.; Astruc, D. Metallocenes as References for the Determination of Redox Potentials by Cyclic Voltammetry - Permethylated Iron and Cobalt Sandwich Complexes, Inhibition by Polyamine Dendrimers, and the Role of Hydroxy-Containing Ferrocenes. *Can. J. Chem.* **2006**, *84*, 288–299, 10.1139/V05-262.

(14) Schmid, L.; Kerzig, C.; Prescimone, A.; Wenger, O. S. Photostable Ruthenium(II) Isocyanoborato Luminophores and Their Use in Energy Transfer and Photoredox Catalysis. *JACS Au* **2021**, *1*, 819–832, 10.1021/jacsau.1c00137.

(15) Frisch, M. J.; Trucks, G. W.; Schlegel, H. B.; Scuseria, G. E.; Robb, M. A.; Cheeseman, J. R.; Scalmani, G.; Barone, V.; Mennucci, B.; Petersson, G. A.; Nakatsuji, H.; Caricato, M.; Li, X.; Hratchian, H. P.; Izmaylov, A. F.; Bloino, J.; Zheng, G.; Sonnenberg, J. L.; Hada, M.; Ehara, M.; Toyota, K.; Fukuda, R.; Hasegawa, J.; Ishida, M.; Nakajima, T.; Honda, Y.; Kiato, O.; Nakai, H.; Vreven, T.; Montgomery, J. A.; Peralta, J. E.; Ogliaro, F.; Kobayashi, R.; Normand, J.; Raghavachari, K.; Rendell, A.; Burant, J. C.; Iyengar, S. S.; Tomasi, J.; Cossi, M.; Rega, N.; Millam, J. M.; Klene, M.; Knox, J. E.; Cross, J. B.; Bakken, V.; Adamo, C.; Jaramillo, J.; Gomperts, R.; Stratmann, R. E.; Yazyev, O.; Austin, A. J.; Cammi, R.; Pomelli, C.; Ochterski, J. W.; Martin, R. L.; Morokuma, K.; Zakrzewski, V. G.; Voth, G. A.; Salvador, P.; Dannenberg, J. J.; Dapprich, S.; Daniels, A. D.; Farkas, Ö.; Foresman, J. B.; Ortiz, J. V.; Cioslowski, J.; Fox, D. J. Gaussian 09. *Gaussian 09, Rev. A. 02* **2009**, No. Gaussian, Inc, Wallingford, CT.

(16) Adamo, C.; Barone, V. Toward Reliable Density Functional Methods without Adjustable Parameters: The PBE0 Model. *J. Chem. Phys.* **1999**, *110*, 6158–6170, 10.1063/1.478522.

(17) Weigend, F.; Ahlrichs, R. Balanced Basis Sets of Split Valence, Triple Zeta Valence and Quadruple Zeta Valence Quality for H to Rn: Design and Assessment of Accuracy. *Phys.*

Chem. Chem. Phys. **2005**, *7*, 3297–3305, 10.1039/b508541a.
